# Paclitaxel Nanomedicines: Molecular Mechanisms of Drug Resistance, Tumor Microenvironment-Responsive Delivery, and Translational Challenges

**DOI:** 10.3390/ijms27177690

**Published:** 2026-08-27

**Authors:** Dejun Cheng, Guowei Yang, Ruibin Kong, Qin Liu, Li Li, Yunpeng Luan, Qijiang Shu

**Affiliations:** 1 Institute of Information, Yunnan University of Chinese Medicine, Kunming 650500, China; 17692399093@163.com (D.C.); yangguowei1213@163.com (G.Y.); k0102030405062026@163.com (R.K.); 2 Key Laboratory of Traditional Chinese Medicine for the Prevention and Treatment of Gastrointestinal Diseases, Yunnan Provincial Department of Education, Kunming 650500, China; lylee1116@126.com (L.L.); luanyunpeng@ynucm.edu.cn (Y.L.); 3 College of Nursing, Yunnan University of Chinese Medicine, Kunming 650500, China; celeryzhaxi@163.com; 4 Yunnan Engineering Research Centre of Preventive Treatment of Chinese Medicine, Kunming 650500, China

**Keywords:** paclitaxel, nanomedicine, pharmaceutical formulation, drug delivery, tumor microenvironment, multidrug resistance, clinical translation, machine learning

## Abstract

Paclitaxel (PTX) remains a major component of treatment for solid tumors, but its clinical performance is limited by poor aqueous solubility, solvent-associated toxicity, heterogeneous tumor exposure, and multifactorial drug resistance. This narrative review examines PTX nanomedicines from a molecular pharmacology perspective, focusing on how carrier design interacts with resistance pathways, tumor microenvironment signals, and intracellular drug trafficking. We outline resistance mechanisms involving ATP-binding cassette subfamily B member 1 (*ABCB1*)/*P-glycoprotein* (*P-gp*)-mediated efflux, microtubule remodeling, apoptosis-related signaling, epigenetic regulation, extracellular matrix deposition, hypoxia, and redox imbalance. We evaluate albumin-bound formulations, liposomes, polymeric micelles, stimuli-responsive carriers, biomimetic systems, carrier-free prodrug assemblies, and multidrug co-delivery platforms according to the molecular and biological barriers they address. Particular attention is given to pH-, redox-, enzyme-, and hypoxia-responsive release; tissue penetration and subcellular localization; and co-delivery of PTX with chemosensitizers, nucleic acids, or pathway-directed agents. Molecular simulation and machine learning are considered as tools for formulation optimization and biomarker-guided patient stratification. These approaches can coordinate drug exposure and resistance modulation in preclinical models, but clinical benefits remain inconsistent. Translation will require reproducible formulations, clinically predictive models, direct measurement of intratumoral drug levels, and validated biomarkers linking molecular delivery mechanisms to patient outcomes.

## 1. Introduction

Malignant tumors remain a leading source of global disease burden and premature mortality. According to the most recent cancer epidemiological data, approximately 20 million new cancer cases and nearly 9.7 million cancer-related deaths are recorded annually worldwide [1,2]. Against this substantial clinical burden, systemic chemotherapy remains an indispensable cornerstone of treatment for the vast majority of solid tumors [3]. Among the diverse arsenal of chemotherapeutic agents, paclitaxel (PTX), a natural diterpenoid initially isolated from the bark of the Pacific yew (Taxus brevifolia), has been one of the most consequential antimitotic drugs since its clinical introduction in 1992 [4,5]. PTX binds to beta-tubulin, promotes microtubule polymerization, and suppresses dynamic depolymerization, thereby disrupting mitotic spindle function and triggering tumor cell death. Owing to its well-characterized cytotoxic activity and broad compatibility with combination regimens, PTX and related taxanes continue to occupy a central position in the treatment of solid tumors, including breast, ovarian, non-small-cell lung, and pancreatic cancers.

However, the clinical application of PTX has long been constrained by the intertwined challenges of unfavorable physicochemical properties, systemic toxicity, and drug resistance. First, PTX exhibits extremely low aqueous solubility; the conventional Taxol formulation relies on Cremophor EL (CrEL) and dehydrated ethanol as a co-solvent system, yet CrEL can provoke hypersensitivity reactions, alter pharmacokinetic behavior, and add to the burden of premedication and infusion management. Second, nonspecific tissue distribution of PTX causes dose-limiting toxicities such as myelosuppression and peripheral neuropathy, which in turn compromise treatment intensity and patient compliance [6,7]. Third, under sustained therapeutic pressure, tumor cells attenuate PTX sensitivity through multiple mechanisms, including drug efflux mediated by *P-glycoprotein* (*P-gp*, encoded by ATP-binding cassette subfamily B member 1 (*ABCB1*)), microtubule-associated alterations, enhanced anti-apoptotic signaling, and physical shielding by the tumor microenvironment (TME) [8]. Thus, the clinical bottleneck for PTX is not simply a solubility problem; it represents a systemic challenge arising from formulation deficiencies, inadequate in vivo delivery, and a complex resistance network.

The advent of nanodrug delivery systems (NDDS) has provided an important path toward improving PTX formulation performance. Early-generation nanoformulations, exemplified by nab-paclitaxel, liposomes, and polymeric micelles, have enhanced clinical usability and tolerability to some extent by eliminating or reducing CrEL, improving drug dispersion, and streamlining administration procedures [9]. Nevertheless, PTX nanoformulations that have reached approval or clinical evaluation exhibit clear limitations: their clinical advantages are concentrated largely in solubilization, toxicity mitigation, and dosing convenience, without consistently achieving genuine tumor-targeted delivery [10]. The marked heterogeneity of the enhanced permeability and retention (EPR) effect in human tumors, together with mononuclear phagocyte system (MPS) clearance, protein corona formation, dense extracellular matrix (ECM), and elevated interstitial fluid pressure (IFP), can all compromise effective nanoparticle accumulation and deep tumor penetration [11]. At the same time, purely physical encapsulation offers little leverage over intracellular resistance nodes such as drug efflux, metabolic adaptation, and anti-apoptotic signaling. Consequently, the development of PTX delivery systems is shifting from a paradigm of “solubilization and toxicity reduction” toward “mechanism-driven precision delivery”.

In recent years, stimuli-responsive materials, biomimetic delivery platforms, active targeting systems, carrier-free prodrug self-assembly, and multi-drug co-delivery strategies have opened new research directions for PTX delivery [12]. TME-responsive carriers attempt to exploit features such as pH, glutathione (GSH), enzymatic activity, hypoxia, and matrix abnormalities to achieve stage-specific regulation of drug release, tissue penetration, and cellular uptake [13]. Multi-drug co-delivery systems further integrate PTX with chemosensitizers, nucleic acid therapeutics, immunomodulatory agents, or physical therapeutic modalities, aiming to intervene in resistance networks through ratio synchronization, subcellular localization, and sequential release [14]. In parallel, molecular dynamics (MD) simulations, machine learning (ML), and artificial intelligence (AI) are being explored as auxiliary tools to resolve carrier–drug interactions, predict formulation parameters, screen candidate systems, and support patient stratification at the translational stage [15]. Although these strategies have shown considerable promise in preclinical models, their clinical translation remains constrained by limitations in model extrapolation, formulation complexity, quality control, and patient heterogeneity.

**Figure 1:**
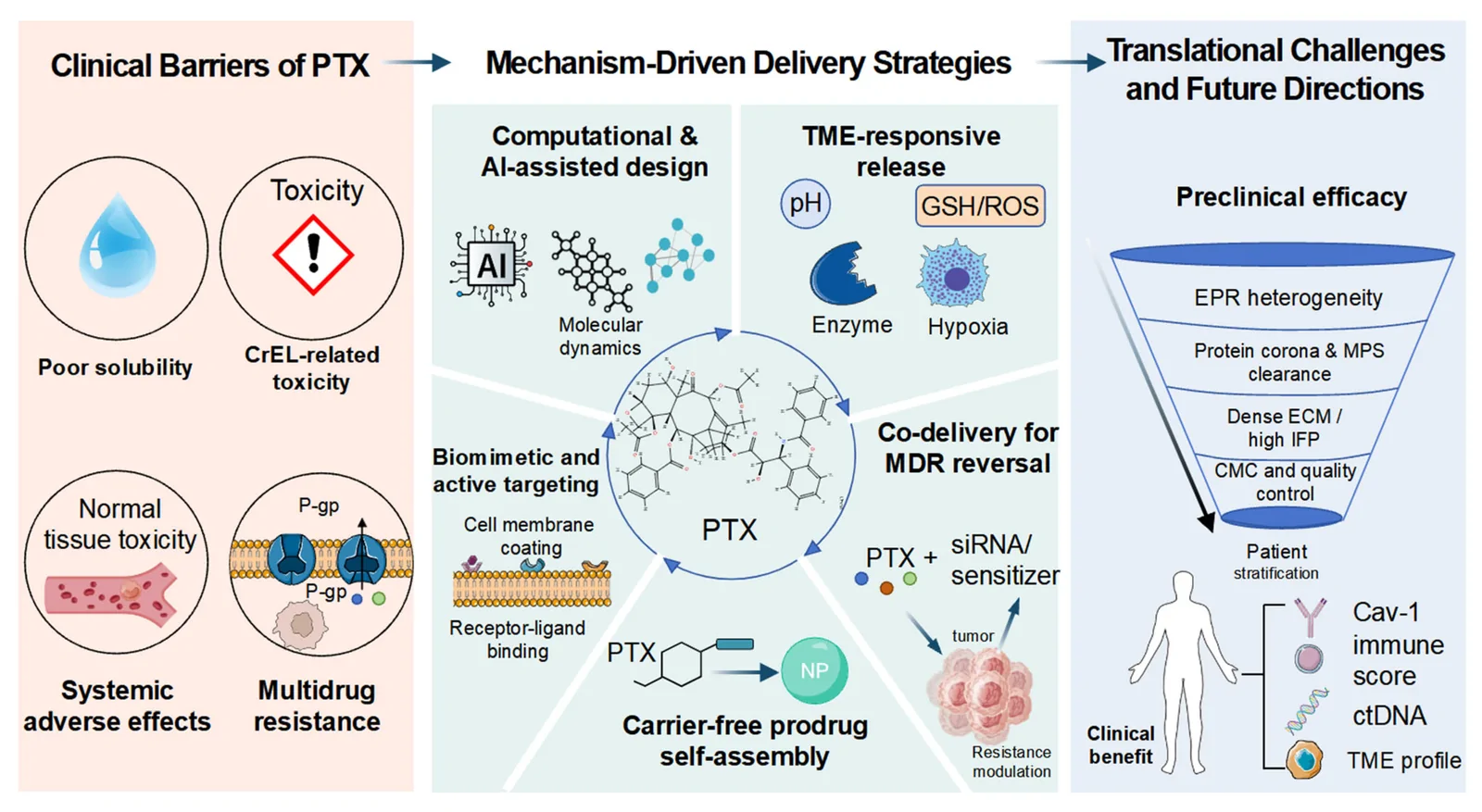
Developmental framework of paclitaxel (PTX) nanodelivery systems, from formulation improvement to precision nanomedicine. The left panel summarizes the core barriers limiting the clinical performance of conventional PTX formulations: extremely low aqueous solubility, hypersensitivity reactions and toxicity induced by the Cremophor EL (CrEL) co-solvent, systemic adverse effects, and multidrug resistance (MDR) mechanisms typified by *P-glycoprotein* (*P-gp*)-mediated drug efflux. The middle panel presents mechanism-driven nanodelivery strategies developed to overcome these barriers, including: tumor microenvironment (TME)-responsive release exploiting endogenous signals such as aberrant pH, glutathione (GSH)/reactive oxygen species (ROS) imbalance, enzyme overexpression, and hypoxia; biomimetic membrane coating and active targeting; carrier-free prodrug self-assembly; and multi-drug co-delivery systems that integrate small interfering RNA (siRNA) or chemosensitizers to synergistically reverse MDR. Computational simulation and artificial intelligence (AI)-assisted development span the entire process from formulation design to translational evaluation. The right panel highlights the principal barriers to clinical translation, including heterogeneity of the enhanced permeability and retention (EPR) effect, protein corona formation, mononuclear phagocyte system (MPS) clearance, dense extracellular matrix (ECM) and elevated interstitial fluid pressure (IFP), chemistry, manufacturing, and controls (CMC) bottlenecks, and insufficient patient stratification and validation of candidate biomarkers, including Caveolin-1 (Cav-1) and circulating tumor DNA (ctDNA). Future development of PTX nanodelivery systems requires establishing a more rigorous evidentiary chain among delivery mechanisms, formulation controllability, and biomarker-guided precision stratification. (This figure was created using Microsoft PowerPoint 2024. Selected graphical elements were sourced from BioIcons (CC BY 3.0/4.0), Noun Project (“molecular” by Anwar Hossain, CC BY 3.0), and BioGDP-licensed materials; BioGDP materials are credited as required: “Created with biogdp.com”).

Against this backdrop, the present review provides a systematic account of the developmental logic and translational challenges of novel PTX delivery systems. We begin by outlining the sources, physicochemical limitations, resistance mechanisms, and systemic toxicities of PTX. We then summarize advances in TME-responsive delivery platforms, biomimetic and active targeting strategies, matrix remodeling approaches, and carrier-free prodrug self-assembly. Building on this foundation, we analyze the rationale for applying multi-drug co-delivery strategies to reverse PTX resistance. Finally, we evaluate the key issues confronting the translation of PTX nanodelivery systems from preclinical design to meaningful patient benefit, drawing on evidence from approved formulations, clinical investigations, translational bottlenecks, and computational and AI-assisted development. The overall logic of this review is illustrated in Figure 1. We aim to emphasize that the core objective for future PTX delivery systems should not be the construction of increasingly complex nanostructures, but rather the establishment of a more verifiable evidentiary chain linking carrier design, in vivo exposure, resistance modulation, and clinical benefit.

To define the scope of this narrative review, PubMed, Web of Science, ScienceDirect, and Google Scholar were searched through June 2026. The principal publication window was January 2016 to June 2026, with particular emphasis on studies published from 2022 onwards. Search terms combined paclitaxel with terms related to nanoformulations, tumor-microenvironment-responsive delivery, multidrug resistance, prodrug self-assembly, multidrug co-delivery, molecular simulation, machine learning, and clinical translation. Peer-reviewed original studies, clinical trials, and reviews directly relevant to PTX pharmacology, delivery-system design, resistance modulation, or clinical translation were prioritized. Earlier landmark studies were additionally retained when necessary to document the discovery of paclitaxel or foundational concepts in drug resistance, tumor penetration, nanocarrier engineering, biomimetic delivery, and computational analysis. Reference lists of relevant articles were also screened to identify additional studies. The final narrative synthesis was supported by approximately 161 cited sources; no quantitative meta-analysis was performed.

## 2. Fundamental Challenges in the Development and Clinical Application of PTX

### 2.1. Biosynthesis Strategies for PTX

PTX was initially obtained primarily through extraction from the bark of the Pacific yew (*Taxus brevifolia*); however, this approach is constrained by the tree’s long growth cycle, the extremely low natural abundance of the active compound, and the attendant resource consumption [16]. Total chemical synthesis, although achievable in the laboratory, involves synthetic routes of extraordinary length and complexity, suffers from low overall yield, and incurs prohibitive cost, rendering it incapable of meeting growing global clinical demand. Consequently, synthetic biology and heterologous metabolic engineering strategies have been progressively developed to achieve efficient biosynthesis of PTX and its precursors in microbial chassis. In particular, the post-genomic elucidation of the PTX biosynthetic pathway, together with the successive discovery of key genes such as *FoTO1* and other cytochrome P450 enzymes, has made the heterologous production of the PTX core scaffold, baccatin III, feasible in microbial chassis including Saccharomyces cerevisiae and Escherichia coli [17]. The construction of such microbial cell factories has shortened the production cycle to a meaningful degree and has laid a foundation for the future large-scale biocatalytic production of PTX and its derivatives [18]. Nevertheless, advances in production methods have not altered the intrinsic pharmacokinetic (PK) profile of PTX in vivo; its delivery efficiency remains the critical factor limiting clinical efficacy.

### 2.2. Mechanisms Underlying the Poor Solubility of PTX

PTX possesses a complex diterpenoid ring structure that confers pronounced hydrophobicity. Its aqueous solubility is extremely low (<0.1 µg/mL), which precludes direct intravenous administration [19]. To overcome this limitation, the first-generation commercial formulation, Taxol, relies on a high-dose co-solvent system composed of Cremophor EL (CrEL) and dehydrated ethanol. However, a substantial body of clinical and pharmacological evidence demonstrates that CrEL is not an inert pharmaceutical excipient. Upon entering the systemic circulation, CrEL readily triggers histamine release from mast cells and basophils, thereby inducing severe immediate-type hypersensitivity reactions. This toxicological profile makes prophylactic high-dose corticosteroids and antihistamines necessary before infusion, which increases the treatment burden and limits further dose escalation.

Beyond inducing acute hypersensitivity, CrEL also distorts the pharmacokinetic behavior of PTX in vivo. Following intravenous infusion, the amphiphilic CrEL molecules spontaneously assemble into thermodynamically stable micellar structures in the blood, entrapping highly hydrophobic PTX within their hydrophobic cores. This micellar sequestration effect reduces the proportion of free, pharmacologically active PTX in plasma, giving rise to the markedly non-linear PK characteristic of this formulation. The process impairs drug distribution to tumor tissue and, because of reduced systemic clearance and a decreased apparent volume of distribution, non-specifically prolongs PTX exposure in the blood and normal tissues, which worsens systemic toxicity. In addition, CrEL can leach di(2-ethylhexyl) phthalate (DEHP) from polyvinyl chloride (PVC) infusion sets during administration, introducing potential risks of hepatotoxicity and reproductive toxicity.

Taken together, the solvent-dependent formulation problems arising from PTX hydrophobicity compromise both safety and pharmacokinetic behavior, representing a major limitation of the conventional PTX formulation. Developing delivery systems that do not depend on CrEL has therefore become a key direction for optimizing the clinical application of PTX.

### 2.3. Mechanisms of PTX Resistance

PTX resistance has long been attributed primarily to drug efflux mediated by *P-glycoprotein* (*P-gp*) [20]. Recent genomic evidence has further revealed that overexpression of this resistance-conferring transporter frequently results from local amplification at the *ABCB1* gene locus [21]. However, as research into tumor microenvironment (TME) heterogeneity has deepened, it has become clear that resistance is far more complex than altered expression of a single protein. Rather, it constitutes a dynamic, evolving process that involves both physical remodeling of the tumor microenvironment and deep intracellular gene-network crosstalk.

At the tissue level, structural changes in the TME can restrict drug penetration. As tumors progress under sustained therapeutic pressure, excessive deposition and collagen crosslinking within the extracellular matrix (ECM) elevate interstitial fluid pressure (IFP) and increase tissue stiffness, thereby impairing drug distribution within the tumor mass. Further biomechanical investigations have proposed a “mechanical assimilation” mechanism: a minority population of cells harboring a resistance phenotype undergoes altered biomechanical properties and, through Merlin-mediated mechanical signal transduction, propagates mechanical stress to neighboring cells [22]. This process remodels the cytoskeletal tension and intercellular interactions of adjacent cells, promotes tissue densification, further restricts drug penetration into deep tumor regions, and correlates with the development of acquired resistance.

At the cellular level, non-coding RNA (ncRNA) networks and epigenetic regulation contribute to the emergence of resistance and confer a degree of adaptability on tumor cells [23]. Beyond gene mutations, tumor cells can attenuate PTX-induced cell-cycle arrest and apoptosis through dynamic epigenetic reprogramming. Multiple studies have demonstrated aberrant expression of specific ncRNAs, including the long non-coding RNA (lncRNA) metastasis-associated lung adenocarcinoma transcript 1 (*MALAT1*) and microRNA (miR-522), in PTX-resistant cell lines [24]. These molecules can modulate miRNA activity through a “molecular sponge” mechanism or influence epigenetic modifications (such as regulation of the methyltransferase euchromatic histone lysine methyltransferase 2 (EHMT2)). Collectively, these ncRNA-driven and epigenetic alterations ultimately drive broad activation of pro-survival signaling pathways, including phosphoinositide 3-kinase/protein kinase B (PI3K/AKT), nuclear factor kappa B (NF-κB), and mitogen-activated protein kinase (MAPK), thereby equipping resistant cells with the capacity to withstand chemotherapeutic stress.

In parallel, post-translational modifications of tubulin also participate in the resistance process. For instance, the kinesin family member 2C (KIF2C) can promote the depolymerization of specific microtubule structures, such as polyglutamylated microtubules, thereby attenuating the microtubule-stabilizing effect of PTX and reducing its pharmacological efficacy [25].

**Figure 2:**
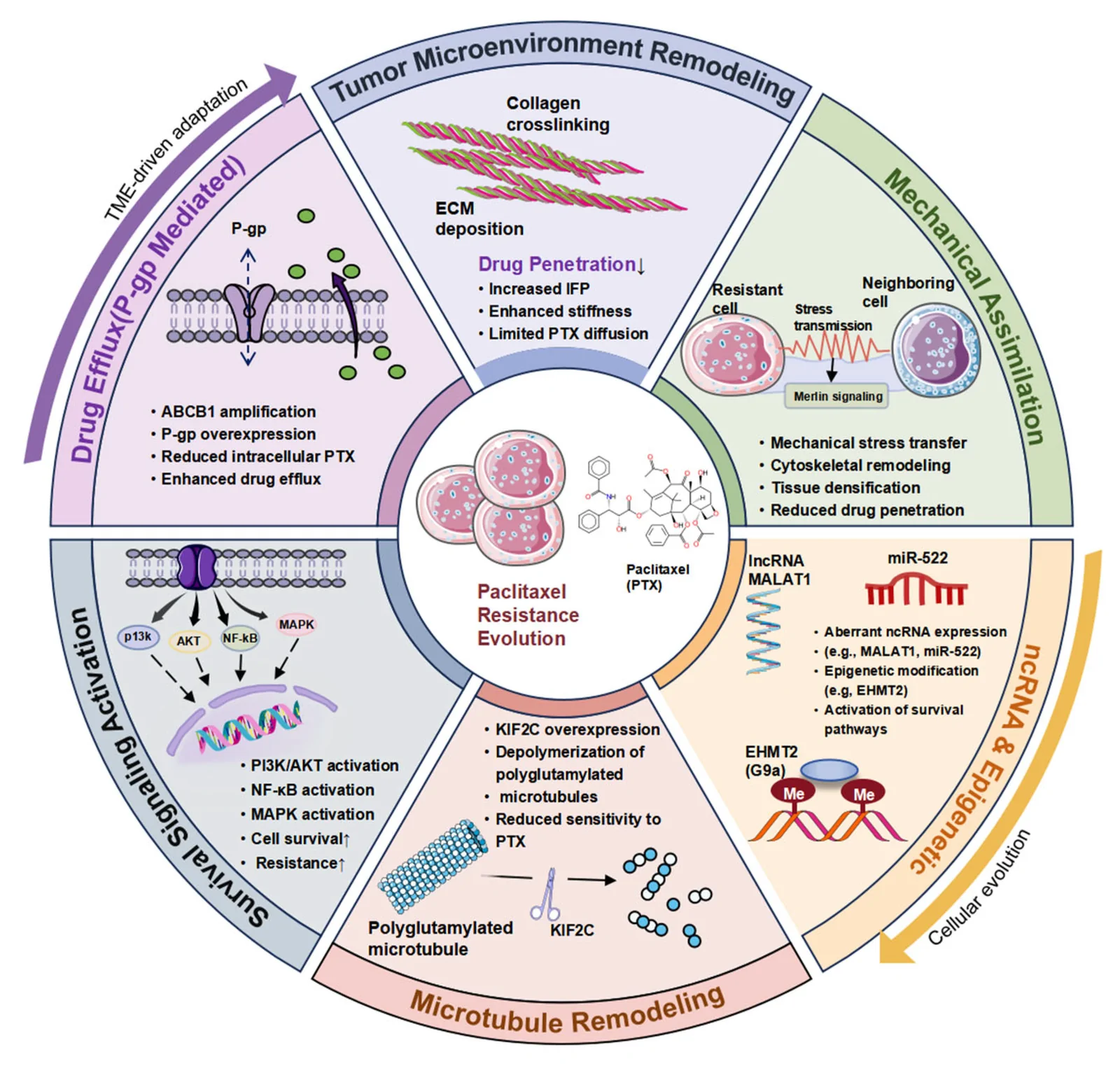
Multi-scale schematic of the mechanisms underlying paclitaxel (PTX) resistance. Clockwise from the upper left, the schematic depicts six interconnected processes that restrict PTX delivery or reduce cellular drug sensitivity. Amplification of *ABCB1* and the resulting overexpression of *P-glycoprotein* (*P-gp*) increase PTX efflux and lower intracellular drug accumulation. Within the tumor microenvironment (TME), extracellular matrix (ECM) deposition and collagen crosslinking increase interstitial fluid pressure (IFP) and tissue stiffness, thereby limiting PTX diffusion. Resistant cells can also transmit mechanical stress to neighboring cells through Merlin signaling, promoting cytoskeletal remodeling and tissue densification. In parallel, ncRNA and epigenetic regulators, including MALAT1, miR-522, and EHMT2, reinforce resistance-associated gene expression and survival signaling. KIF2C-mediated depolymerization of polyglutamylated microtubules weakens the microtubule-stabilizing activity of PTX, whereas activation of the PI3K/AKT, NF-κB, and MAPK pathways supports cell survival during treatment. The outer arrows indicate the interaction between TME-driven adaptation and cellular evolution, which together sustain the resistant phenotype. Upward arrows (↑) indicate increased expression or activity, whereas downward arrows (↓) indicate decreased expression or activity. (Figure drawn by the authors using Microsoft PowerPoint 2024. Selected vector elements were obtained from Servier Medical Art [CC BY 4.0] and the BioIcons repository and used under their respective licenses; all other elements and the overall layout were created by the authors).

In summary, as illustrated in Figure 2, PTX resistance is not driven by a single mechanism but instead involves a multi-layered, dynamically evolving process encompassing enhanced drug efflux, TME remodeling, mechanical assimilation, ncRNA and epigenetic dysregulation, activation of pro-survival signaling, and altered microtubule dynamics. These mechanisms interact with one another and collectively reduce the effective exposure of drug within tumor tissue, ultimately promoting the formation and maintenance of the resistance phenotype. A deeper mechanistic understanding of these processes not only helps to clarify the biological basis of PTX resistance, but also provides an important conceptual foundation for the design of multi-drug co-delivery strategies and intelligent nanodelivery systems, as discussed in subsequent sections.

### 2.4. Mechanisms of PTX-Induced Systemic Toxicity

In clinical practice, PTX-associated chemotherapy-induced peripheral neuropathy (CIPN) represents one of the foremost dose-limiting toxicities, frequently compelling dose reduction or treatment discontinuation. Pharmacokinetic–pharmacodynamic (PK-PD) modeling indicates that both the maximum plasma concentration (C~max~) and the overall systemic exposure to PTX correlate with the risk of CIPN onset [26]. Early investigations attributed CIPN pathogenesis solely to excessive microtubule stabilization by PTX within sensory neurons, which was thought to impair axonal transport and intracellular trafficking [27]. However, advances in neuroimmunology have established that neuro-immune interactions are a key driver of chronic hyperalgesia.

At the molecular level, PTX can interact with multiple receptors, including C5a receptor 1 (C5aR1) [28] and Toll-like receptor 4 (TLR4). Furthermore, multi-omics and machine learning analyses have identified sphingosine-1-phosphate receptor 1 (S1PR1) as another key candidate mediator of PTX-induced peripheral neuropathy [29].

At the cellular level, PTX can induce activation of both peripheral and central immune cells. For example, macrophages surrounding the dorsal root ganglion (DRG) are extensively recruited and polarized toward a pro-inflammatory phenotype. At the same time, PTX can penetrate the relatively permeable blood–nerve barrier (BNB), enter the central nervous system, and activate astrocytes in the spinal dorsal horn [30]. These activated glial cells release abundant pro-inflammatory cytokines, such as tumor necrosis factor-α (TNF-α), and chemokines, such as C-X-C motif chemokine ligand 12/stromal cell-derived factor-1 (CXCL12/SDF-1). These inflammatory mediators act on neuronal receptors, including C-X-C motif chemokine receptor 4 (CXCR4), to modulate neural signaling and have been linked to pain sensitization [31].

At the pathway level, systemic metabolic dysregulation and aberrant activation of specific signaling cascades further amplify neurotoxicity. For instance, Xie et al. demonstrated in a recent breast cancer model that PTX induces aberrant expression of Dickkopf-1 (DKK1), and that targeted antagonism of this signal not only blocks neurodegenerative changes but also synergistically enhances the antitumor activity of PTX [32]. In parallel, work by Zhang et al. revealed that pharmacological activation of neuronal protective autophagy can effectively clear mitochondria damaged by PTX and accumulating within axons [33]. PTX-induced CIPN is therefore tied to a multi-level biological cascade involving neuronal injury, immune cell activation, and amplification of inflammatory signaling, and it is a major factor constraining the clinical utility of PTX.

### 2.5. From Barriers to Design Principles

The challenges described above, which include TME physical barriers, intracellular resistance networks, and neuro-immune interactions, together exceed the intervention capacity of traditional nanoformulations that rely on passive targeting (such as the enhanced permeability and retention (EPR) effect) or simple physical encapsulation. Consequently, the design of PTX delivery systems has progressively shifted from merely improving solubility and circulatory stability toward staged regulation that exploits TME characteristics. By responding to endogenous signals such as local tumor pH, redox status, enzyme overexpression, hypoxia, and matrix abnormalities, next-generation nanocarriers may achieve more selective drug release, tissue penetration, and cellular uptake within the tumor region. Building on this rationale, the following sections focus on responsive delivery platforms designed to address the complex barriers imposed by the TME.

## 3. Responsive Delivery Platforms Designed to Overcome Complex TME Barriers

### 3.1. Stimuli-Responsive Nanodelivery Systems

**Figure 3:**
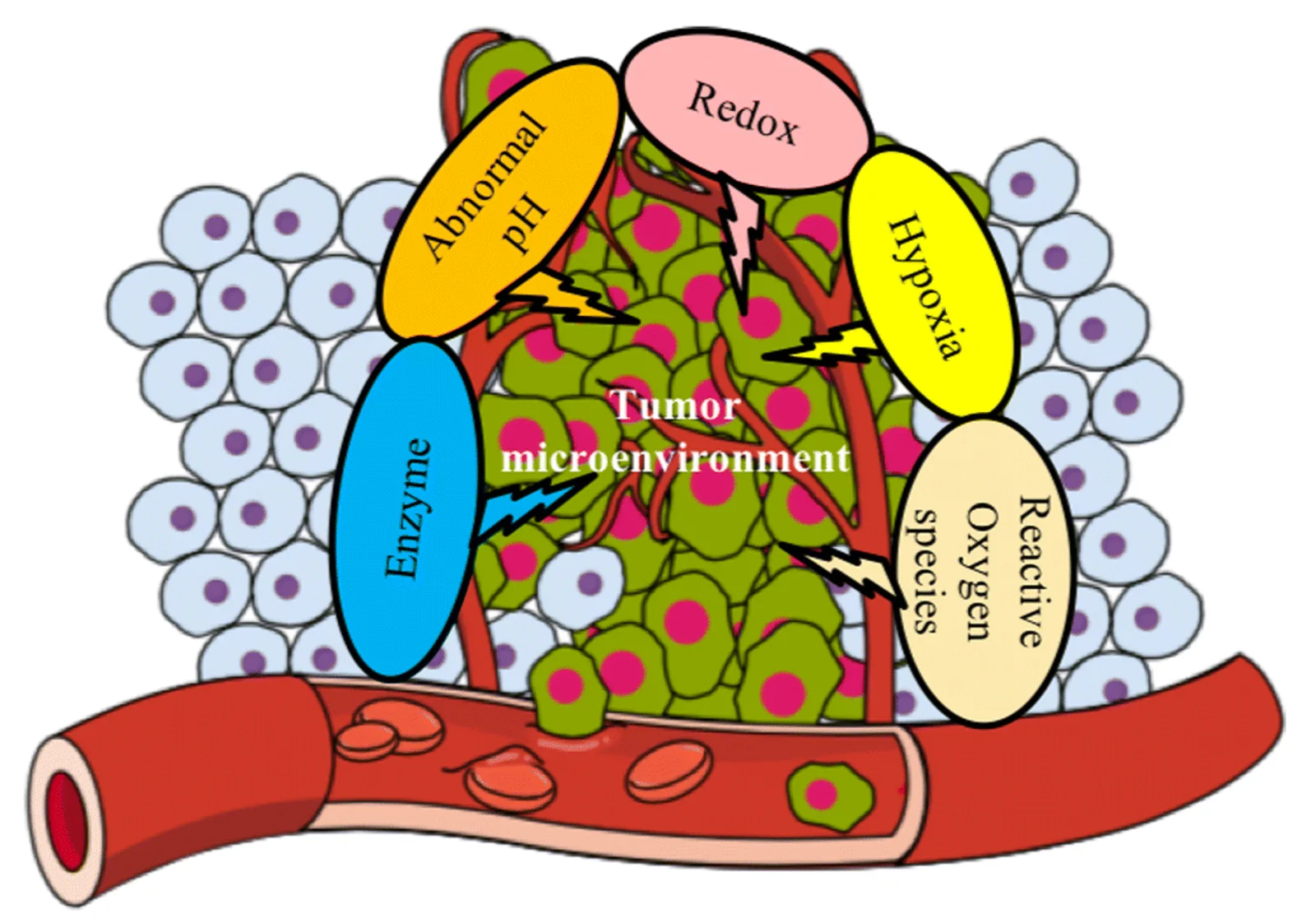
Schematic of key endogenous stimulatory signals in the tumor microenvironment (TME) and their triggering mechanisms for responsive nanodelivery systems. The green cell cluster at the center represents the solid tumor parenchyma; the surrounding light-blue cells represent non-tumor components in the TME, such as stromal cells or immune cells; and the red vessels at the bottom represent aberrant tumor neovasculature, which serves as the major conduit for nanocarrier entry into tumor tissue following intravenous administration. The five ellipses summarize the principal endogenous stimulatory signals within the TME that can be harnessed to trigger structural transformations or drug release from nanodelivery systems, including mildly acidic pH (aberrant pH), redox imbalance, enzyme overexpression, hypoxia, and reactive oxygen species (ROS) accumulation; the lightning-bolt symbols indicate that these aberrant signals can function as trigger switches for nanocarrier responsiveness and drug release. Specifically, extracellular acidic pH (pHe) and enzyme signals such as matrix metalloproteinase-2/9 (MMP-2/9) are predominantly distributed in the extracellular interstitium and can induce acid–labile bond cleavage, surface charge reversal, carrier de-shielding, or size shrinkage, thereby promoting tumor tissue penetration and cellular uptake. Intracellularly, high concentrations of glutathione (GSH) and ROS can trigger the cleavage of redox-sensitive linkages, promoting prodrug or carrier disassembly and drug release. Hypoxic signals, which are mainly present in deep tumor regions distant from blood vessels, can activate hypoxia-sensitive structures such as azobenzene to achieve selective drug release in deep regions. Although the spatial distribution of these signals within the TME is heterogeneous, together they constitute the biochemical basis for responsive paclitaxel (PTX) nanodelivery systems to achieve staged on-demand drug release, deep tumor penetration, and intracellular drug liberation. Reproduced from Ref. [34] under a CC BY 4.0 License.

The tumor microenvironment (TME) contains several endogenous signals that can serve as triggers for tumor-targeted paclitaxel (PTX) release: a highly reducing intracellular milieu (intracellular glutathione (GSH) concentrations can reach 100- to 500-fold those of normal extracellular compartments), overexpression of proteases such as matrix metalloproteinases (MMPs, prominently MMP-2 and MMP-9), and regional hypoxia. As illustrated in Figure 3, the spatial heterogeneity of the TME (encompassing hypoxia, redox imbalance, reactive oxygen species (ROS) accumulation, and protease overexpression) provides a critical endogenous trigger environment for nanocarriers. Through rational design, these stimulatory signals can induce structural transformations and on-demand drug release from PTX nanocarriers at different stages of the delivery process, thereby overcoming the limitations inherent in conventional passive targeting and enabling selective drug accumulation and precision delivery within the tumor region [34]. Stimuli-responsive delivery strategies built on these signals enable selective PTX release at the tumor site while minimizing non-specific exposure in the systemic circulation. By the nature of the triggering signal, current strategies fall into three categories: redox-responsive, enzyme-responsive, and hypoxia-responsive. These three are spatially complementary with respect to their sites of drug release. Redox-responsive systems target the intracellular high-GSH environment, enzyme-responsive systems act within the extracellular tumor interstitium, and hypoxia-responsive systems are directed toward poorly perfused, deep tumor regions.

#### 3.1.1. Redox-Responsive Delivery Systems

Redox-responsive strategies exploit the differential in GSH concentration between the intracellular and extracellular compartments of tumor cells to regulate drug release. GSH concentrations in normal extracellular tissue are only 2–20 μM, whereas intracellular levels in tumor cells can reach 2–10 mM, a gradient that provides a basis for intracellular-specific drug release [13]. In the design of PTX prodrugs, disulfide bonds are frequently incorporated as linker arms between PTX and hydrophobic moieties, allowing the prodrug to undergo cleavage and release active drug under high-GSH conditions. For example, Zhang et al. covalently linked PTX to a branched fatty acid via a disulfide bond to construct PTX-ss-BAC18 prodrug nanoassemblies, which achieved superior tumor suppression compared with Taxol in vivo while exhibiting lower systemic toxicity [35]. Beyond direct modification of PTX, disulfide bonds can also be integrated into the carrier scaffold. Du et al. constructed redox-responsive liposomes (SS-LP) in which disulfide-bearing phosphatidylcholine replaced conventional phospholipids; these liposomes underwent structural disintegration and PTX release under reducing conditions, and their in vivo tumor-suppressive efficacy surpassed that of both non-responsive liposomes and Taxol [36].

Beyond sole GSH responsiveness, disulfide bonds have also been shown to possess dual reductive and oxidative sensitivity. Sun et al. demonstrated that disulfide bonds can be oxidatively cleaved under high-ROS conditions and that the position of the linkage affects the drug release rate, thereby providing a structural rationale for the design of GSH/ROS dual-responsive systems [37,38]. However, redox-responsive systems generally depend on endocytosis of the nanocarrier by tumor cells to trigger drug release; their delivery efficiency may therefore remain constrained in solid tumor regions with poor vascularization or dense extracellular matrix (ECM). Enzyme-responsive systems that rely on extracellular interstitial signals such as MMPs can initiate drug release or carrier structural transformation within the tumor interstitium before cellular uptake, and they therefore complement redox-responsive strategies.

#### 3.1.2. Enzyme-Responsive Delivery Systems

Enzyme-responsive strategies principally exploit the high expression of MMP-2 and MMP-9 in the tumor interstitium as triggering signals. Cleavage of specific peptide sequences enables drug release or carrier structural remodeling. The expression levels of MMP-2/9 in the interstitium of multiple solid tumor types are markedly higher than those in normal tissues, and these enzymes can efficiently cleave specific substrate sequences such as GPLGIAGQ or GPLGVRG under physiological conditions, which achieves tumor-specific triggering [39,40].

To enhance drug retention within the tumor locale, researchers have developed an MMP-triggered morphological transformation strategy. In this system, cleavable peptide-crosslinked polymeric micelles serve as the core; following MMP-mediated cleavage in the tumor interstitium, small nanoparticles form micron-scale aggregates that sustain the release of covalently conjugated PTX. This system achieved a maximum tolerated dose of 240 mg/kg (PTX equivalent), roughly 16-fold that of the clinical Taxol formulation, and produced pronounced tumor growth inhibition after intratumoral injection in a fibrosarcoma model [41]. In addition, other TME-associated enzymes, including hyaluronidase (such as PEGylated recombinant human hyaluronidase, PEGPH20) and cathepsin B, have been used to construct enzyme-responsive systems. These enable enhanced cellular uptake and intralysosomal drug release through degradation of the hyaluronic acid (HA) shell or peptide cleavage, respectively [34,42]. Compared with MMP-responsive strategies, such systems can additionally exploit cancer cell surface receptors to participate in the endocytic process; however, their delivery efficacy remains subject to variation in enzyme expression levels across different tumor types.

The main limitation of enzyme-responsive strategies is the marked heterogeneity in the expression of target enzymes such as MMPs across tumor types and individual patients, which affects both triggering efficiency and the feasibility of indication selection and efficacy prediction. For deep tumor regions characterized by poor blood supply and severe hypoxia, the coverage capacity of enzyme-triggered drug release is likewise constrained. For such regions, hypoxia-specific responsive strategies offer a complementary dimension to the solution space.

#### 3.1.3. Hypoxia-Responsive Delivery Systems

Hypoxia-responsive strategies are designed primarily for poorly perfused, deep tumor regions. The azobenzene group (–N=N–) is relatively stable under normoxic conditions but can undergo reductive cleavage in severely hypoxic environments, catalyzed by nitroreductases and related enzymes, to generate aniline derivatives. It has therefore been widely adopted for constructing hypoxia-responsive prodrug linkages.

In a prodrug self-assembly system, PAP nanoparticles link PTX to a polyethylene glycol (PEG) chain through an azobenzene group, enabling the release of active PTX via reductase-mediated cleavage in hypoxic regions. Optimization of the PEG chain length to 750 yielded a drug loading capacity of 44 wt%, with over 70% drug release within 24 h under hypoxic conditions; the in vivo tumor-suppressive effect was markedly superior to that observed under normoxic conditions [43].

To further improve hypoxia-triggering efficiency, a nanoparticle system co-encapsulating the photosensitizer chlorin e6 (Ce6) and an azobenzene-bearing PTX prodrug exploits the localized oxygen consumption that occurs during photodynamic therapy (PDT) to intensify the hypoxic state within the tumor region, thereby promoting azobenzene cleavage and PTX release and enabling combined chemotherapy and PDT at relatively low light doses. Hypoxia-responsive strategies have also been applied to improve the distribution of photosensitizers in low-oxygen regions. A human serum albumin-based nanodelivery system can trigger Ce6 release under hypoxic conditions and promote its diffusion within tumor tissue, yielding superior combined therapeutic efficacy in both tumor spheroid models and mouse xenograft models [44]. The effectiveness of hypoxia-responsive systems still depends, however, on the ability of the nanocarrier to reach the hypoxic deep tumor regions, a process that is susceptible to inadequate tumor vascularization and dense stromal barriers. When nanocarriers cannot effectively penetrate into deep tumor regions, the impact of the hypoxia-triggered mechanism will likewise be compromised.

The three classes of stimuli-responsive strategies, redox-responsive, enzyme-responsive, and hypoxia-responsive, correspond, respectively, to the intracellular high-GSH environment, the extracellular interstitial enzyme milieu, and the hypoxic deep tumor regions, and are thus spatially complementary with respect to their triggering sites. The abundance and spatial distribution of TME triggers are neither uniform nor static. Acidity, redox state, protease activity, and oxygenation can differ across tumor types and across regions within the same tumor [13,34,39,40,41]. Consequently, a carrier designed around a single activation threshold may undergo incomplete, asynchronous, or spatially restricted activation. Such heterogeneity may reduce the reliability and reproducibility of stimulus-responsive PTX release and limit extrapolation from simplified cell-culture and xenograft models. Clinical translation will therefore require activation thresholds to be evaluated across physiologically relevant ranges, delivery performance to be tested in heterogeneous or patient-derived models, and relevant TME signals to inform indication or patient selection where feasible.

A shared prerequisite for all three, however, is that nanocarriers must first reach and distribute within the tumor tissue. Clearance by the mononuclear phagocyte system (MPS) in the systemic circulation, together with non-specific distribution arising from physicochemical properties such as particle size and surface charge, remain critical factors limiting delivery efficiency. Against this backdrop, strategies that dynamically modulate nanocarrier size and surface charge, so as to separately optimize circulatory stability and tumor tissue penetration, have emerged as an important research direction for improving delivery efficiency, and are discussed in the following section.

### 3.2. Size- and Charge-Transitioning Delivery Systems

**Figure 4:**
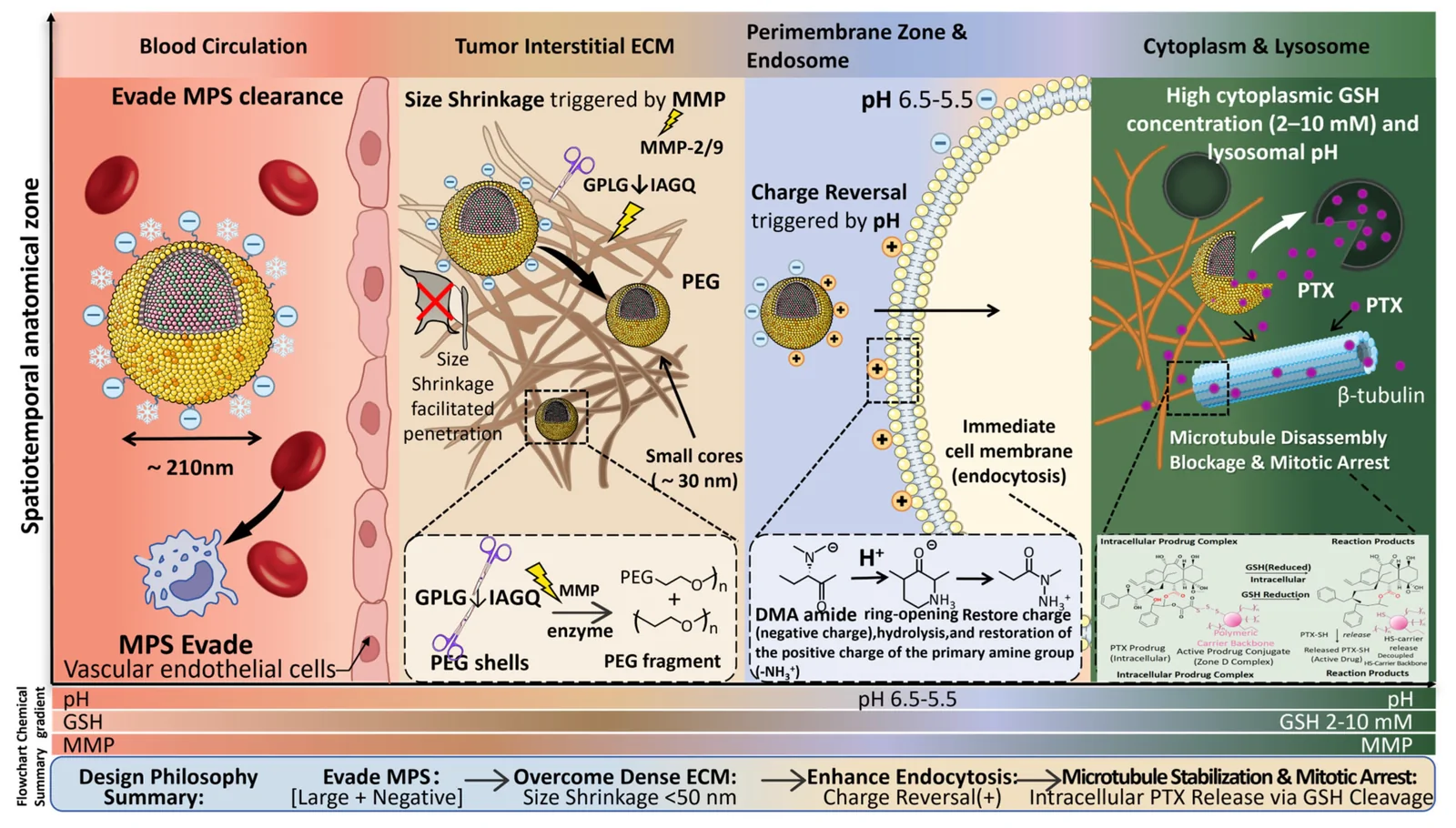
Schematic of size–charge dynamic transition and cascade delivery mechanisms of intelligent paclitaxel PTX nanocarriers. The schematic is read from left to right across four anatomical and cellular compartments: blood circulation, the tumor interstitial extracellular matrix (ECM), the perimembrane/endosomal region, and the cytoplasm/lysosome. During circulation, the polyethylene glycol (PEG)-modified nanocarrier has a relatively large diameter (approximately 210 nm) and a negative surface charge. The PEG corona shields the particle surface and reduces recognition and clearance by the mononuclear phagocyte system (MPS), thereby supporting systemic persistence. After passage through the tumor vascular endothelium, the carrier encounters matrix metalloproteinase-2/9 (MMP-2/9) in the tumor ECM. Cleavage of the MMP-responsive GPLGIAGQ peptide linker removes the PEG shell and promotes dissociation of the larger particle into small cores of approximately 30 nm, which can move more readily through the dense extracellular matrix. The red X in the ECM region indicates restricted penetration of intact larger nanoparticles through the dense stromal matrix before MMP-triggered size reduction. Near the cell membrane and during endosomal trafficking, the acidic environment (pH 6.5–5.5) hydrolyzes the 2,3-dimethylmaleic anhydride (DMA)-derived amide groups. This reaction restores protonated primary amines and changes the carrier surface from negative to positive, increasing membrane association and facilitating endocytic uptake. In the intracellular compartment, the high cytoplasmic glutathione concentration (GSH; approximately 2–10 mM), together with the acidic lysosomal environment, promotes cleavage of redox-sensitive linkers and PTX release. The released PTX binds to β-tubulin, prevents microtubule disassembly, and induces mitotic arrest. The chemical-gradient bars at the bottom relate these transformations to changes in MMP activity, pH, and GSH concentration across the delivery route. The accompanying design summary highlights the functional role of each state: the larger PEGylated particle favors circulation, the smaller cores improve ECM penetration, the positively charged surface promotes cellular uptake, and intracellular linker cleavage releases PTX at its site of action. (Figure drawn by the authors using Microsoft PowerPoint 2024. Selected graphic elements were obtained from BioIcons [CC BY 4.0] and the SciDraw open-access repository [https://doi.org/10.5281/zenodo.3926231, https://doi.org/10.5281/zenodo.3926081, and https://doi.org/10.5281/zenodo.10118509; CC BY 4.0]).

Nanocarrier size and surface charge influence circulation, extravasation, interstitial transport, and cellular uptake, but no universal optimum applies across carrier platforms or tumor types. In many experimental systems, relatively larger particles with neutral or weakly negative surfaces may favor systemic persistence, whereas smaller particles in the tens-of-nanometers range can facilitate interstitial penetration; cationic surfaces may enhance membrane association and cellular uptake [45,46,47]. These relationships should be regarded as context-dependent design heuristics rather than fixed requirements. Carrier composition, morphology, protein–corona formation, and tumor-specific vascular and extracellular-matrix properties can alter how nominal particle size or surface charge affects in vivo delivery [11,45,47]. Dynamic size- or charge-transitioning systems therefore seek to reconcile competing stage-specific requirements, but their performance must be validated in the relevant biological context [47,48]. Figure 4 illustrates this stage-dependent design logic as a spatiotemporal cascade spanning circulation, tumor penetration, cellular uptake, and intracellular PTX release.

#### 3.2.1. Size-Shrinking Delivery Systems

Size-shrinking strategies introduce TME-responsive structural elements that allow the carrier to maintain a relatively large particle diameter during circulation while undergoing triggered size reduction at the tumor site to enhance deep tissue penetration. Studies have demonstrated that nanosystems capable of size transitioning can achieve deeper tumor tissue penetration than carriers with a fixed particle size [46]. For instance, Sun et al. constructed MMP-2-responsive size-switchable nanoclusters that, upon MMP-2 triggering in the tumor interstitium, dissociate from approximately 210 nm to approximately 30 nm and release PTX-loaded small micelles, thereby increasing PTX distribution in the deep regions of solid tumors [49].

In enzyme-responsive size-shrinking strategies, MMPs highly expressed in the tumor interstitium are commonly used to cleave the carrier shell and trigger particle size reduction. By linking a hydrophilic shell such as PEG to the nanoparticle core via an MMP-cleavable peptide, the particle undergoes enzymatic de-shielding in the tumor interstitium, with its diameter contracting from the hundred-nanometer range to tens of nanometers. This alleviates ECM-imposed diffusion constraints and facilitates PTX penetration toward regions distant from blood vessels [47]. Building on this principle, the introduction of cascade-responsive mechanisms, such as the synergistic action of MMPs and ROS, can further induce multi-stage size reduction and improve drug distribution in deep tumor regions. pH-sensitive chemical bonds, including hydrazone and β-carboxylic amide linkages, can also induce PEG shedding under mildly acidic conditions, converting larger particles into smaller ones. In addition, redox-responsive systems exploit the high intracellular GSH concentrations in tumor cells to trigger prodrug disassembly. A dynamic prodrug system based on maleimide–albumin covalent conjugation can bind to albumin in the circulation to form nanoaggregates that prolong in vivo residence time; upon entry into tumor cells, disulfide bond cleavage releases the active drug [48].

#### 3.2.2. Charge-Reversal Delivery Systems

Charge-reversal strategies introduce TME-responsive functional groups that allow nanoparticles to maintain a neutral or weakly negative surface charge during circulation while converting to a positive charge at the tumor site to promote cellular internalization [45]. Among the available approaches, 2,3-dimethylmaleic anhydride (DMA) modification is one of the more common implementations. DMA can confer a negative surface charge on the carrier under physiological pH conditions; upon hydrolysis in the mildly acidic tumor environment, cationic groups are exposed, thereby inducing charge reversal and enhancing cellular uptake [50]. Studies have shown that pH-responsive charge-reversal micelles co-loaded with PTX and resistance-modulating agents can enhance cellular uptake and inhibit *P-gp*-mediated drug efflux, thereby effectively reversing multidrug resistance (MDR). Building on this, nucleic acid therapeutics can also be co-delivered with PTX, for example, through small interfering RNA (siRNA)-mediated modulation of apoptosis-related pathways, while simultaneously improving cellular uptake efficiency and drug sensitivity. Beyond pH triggering, charge reversal can also be achieved through enzyme- or redox-responsive mechanisms. Enzyme-responsive systems can exploit MMP-2-mediated degradation of the outer shell to trigger morphological transformation, thereby enhancing tumor retention; in redox-responsive systems, disulfide bond cleavage under the high GSH conditions within tumors leads to size reduction and exposure of cationic groups, thereby improving cellular uptake and deep penetration capacity [51,52]. It is worth noting that protein corona formation may shield nanoparticle surface charge in vivo, rendering the charge-reversal effect weaker than that observed in vitro; the actual delivery efficiency therefore requires further evaluation in the context of the in vivo environment.

#### 3.2.3. Combined Size–Charge Transition Systems

Size shrinking and charge reversal correspond to distinct stages of the delivery process: the former primarily improves tissue penetration, whereas the latter enhances cellular uptake. Combined transition strategies integrate both within a single system, enabling size reduction, charge reversal, and intracellular drug release to be triggered sequentially at different delivery stages.

Wang et al. constructed an enzyme/pH dual-responsive nanosystem in which MMP-mediated shell degradation achieves size reduction, and the acidic environment of intracellular organelles triggers drug release; this system exhibited superior in vivo antitumor efficacy compared with single-responsive systems [53]. Another class of strategy combines pH-responsive charge reversal with GSH-responsive drug release, sequentially triggering enhanced endocytosis and intracellular release at different stages, and has been applied to multi-drug synergistic action. The introduction of multiple responsive elements increases the structural complexity of the system and also raises the difficulty of predicting in vivo behavior and ensuring quality control. Under conditions in which TME signals exhibit heterogeneity, the actual efficiency of dual-trigger strategies still requires further validation in models more closely approximating the clinical setting.

The strategies described above primarily focus on optimizing PTX delivery efficiency during the circulatory, tumor penetration, and cellular uptake stages. However, the first challenge nanocarriers face upon entering the systemic circulation remains recognition and clearance by the immune system. Natural cell membrane coating strategies, by endowing carriers with biological interfacial camouflage, can reduce MPS recognition efficiency to a meaningful degree; these are discussed in the following section.

### 3.3. Biomimetic Membrane Coating and Active Targeting Strategies

#### 3.3.1. Natural Cell Membrane Coating Strategies

Nanocarriers entering the systemic circulation are susceptible to recognition and clearance by the MPS, and the passive targeting mediated by the conventional enhanced permeability and retention (EPR) effect varies considerably across tumor types and individual patients. Natural cell membrane coating technology, by directly transferring biological membrane structures onto the surface of nanocarriers, endows them with innate immune evasion capability [54]. The red blood cell (RBC) membrane was among the first coating materials to be systematically investigated. Cluster of differentiation 47 (CD47) protein on the RBC membrane surface can engage the signal regulatory protein alpha (SIRPα) receptor on macrophages, thereby inhibiting phagocytic recognition; simultaneously, the native glycocalyx structure reduces opsonin adsorption through steric hindrance. In vivo experiments have confirmed that RBC membrane coating can prolong the circulation time of PTX, increase drug accumulation at the tumor site, and reduce systemic toxic side effects [55].

**Figure 5:**
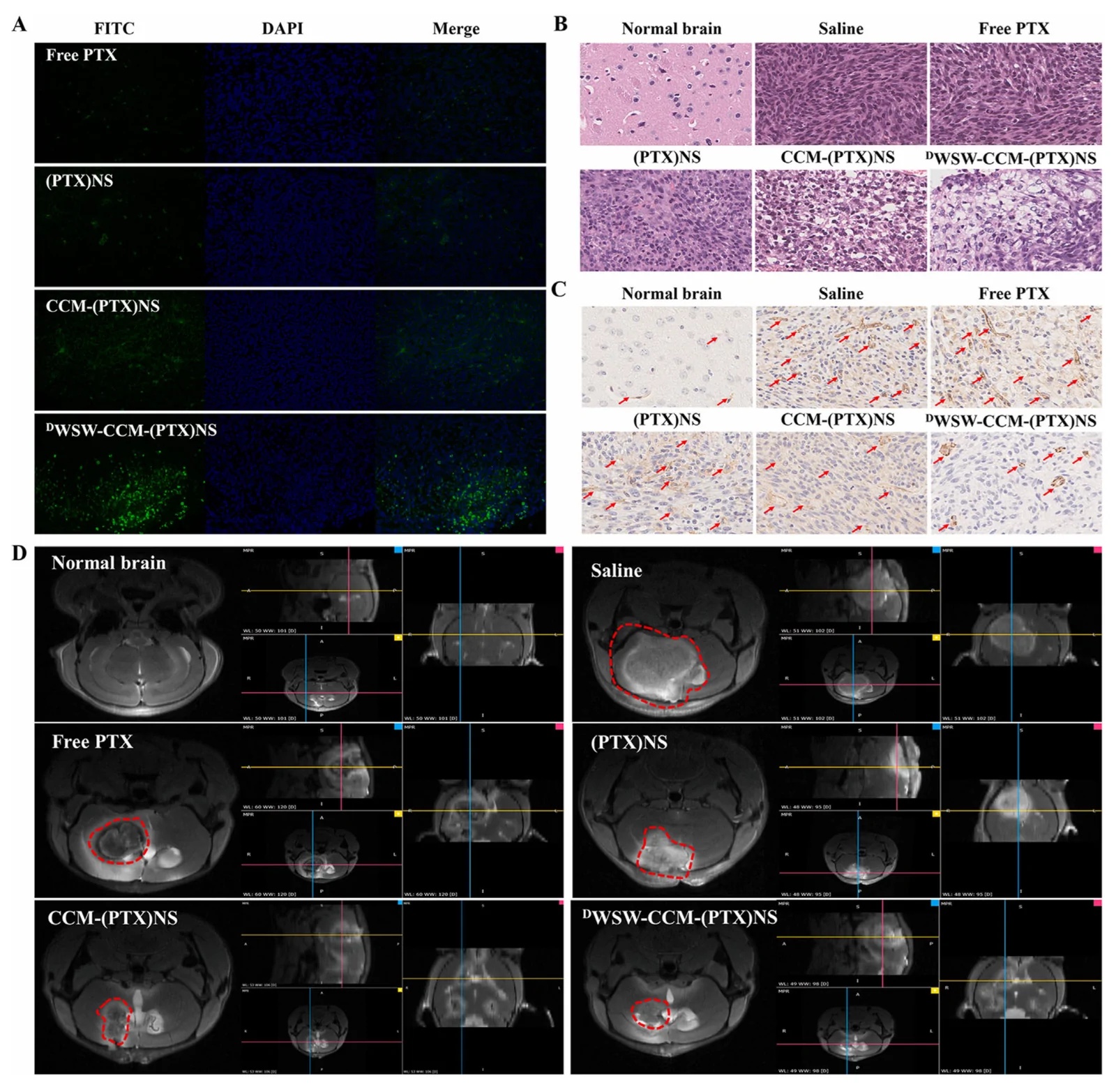
Comprehensive assessment of in vivo antitumor efficacy of DWSW penetrating peptide-modified cancer cell membrane biomimetic paclitaxel (PTX) nanosuspension in a C6 glioma-bearing mouse model. Treatment groups include normal brain tissue control (Normal brain), saline control (Saline), free paclitaxel (Free PTX), plain PTX nanosuspension ([PTX]NS), cancer cell membrane (CCM)-coated PTX nanosuspension (CCM-[PTX]NS), and DWSW-modified CCM-(PTX)NS (DWSW-CCM-(PTX)NS). (**A**) Tumor tissue terminal deoxynucleotidyl transferase-mediated dUTP nick-end labeling (TUNEL) staining: green fluorescence (fluorescein isothiocyanate (FITC) channel) represents apoptotic cells, blue fluorescence marks nuclei stained with 4′,6-diamidino-2-phenylindole (DAPI), and Merge shows the overlay; the DWSW-CCM-(PTX)NS group exhibited the strongest apoptotic signal. (**B**) Hematoxylin and eosin (H&E) staining: reflecting cellular morphology and the degree of necrosis in tumor tissue across groups; the DWSW-CCM-(PTX)NS group displayed the most pronounced tumor cell destruction. (**C**) CD31 immunohistochemical staining: brown signal and red arrows indicate CD31-expressing tumor neovascular endothelial cells; the density of CD31-positive areas reflects the level of tumor microvessel formation; the DWSW-CCM-(PTX)NS group showed the lowest microvascular marker expression. (**D**) Magnetic resonance imaging (MRI): the blue, pink, and yellow lines are MRI localizer lines showing corresponding slice positions across the axial, sagittal, and coronal views and do not represent biological signals; the red dashed contours delineate the tumor regions within the brain. The DWSW-CCM-(PTX)NS group exhibited the smallest tumor volume. These results, spanning tumor cell apoptosis, histopathological damage, microvessel formation, and MRI-based volumetric assessment, demonstrate that cancer cell membrane biomimetic coating and DWSW penetrating peptide functionalization can synergistically enhance the in vivo antitumor efficacy of PTX nanosuspension in a glioma model. Reproduced from Ref. [58] under a CC BY 4.0 License.

RBC membranes, however, lack intrinsic tumor-specific recognition capacity. To address this, researchers have introduced cancer cell membrane (CCM) coating strategies, which exploit homologous adhesion molecules on the tumor cell surface, such as epithelial cell adhesion molecule (EpCAM) and differentiation 44 (CD44), to promote active uptake by tumor cells [56]. Wu et al. developed a homologous CCM-coated metal–organic framework (MOF) nanoplatform, in which cancer cell membrane surface proteins mediate homotypic targeting to increase drug enrichment in homologous tumor cells [57]. To address the difficulty of drug delivery to the brain, Fan et al. constructed a DWSW penetrating peptide-modified C6 glioma membrane-coated PTX nanosuspension. As illustrated in Figure 5, compared with free PTX, PTX nanosuspension (NS), and CCM-coated PTX NS (CCM-PTX NS), the DWSW-modified CCM-PTX NS exhibited the strongest terminal deoxynucleotidyl transferase-mediated dUTP nick-end labeling (TUNEL) apoptotic signal in brain tissue; hematoxylin and eosin (H&E) staining, cluster of differentiation 31 (CD31) immunohistochemistry, and magnetic resonance imaging (MRI) consistently confirmed that this formulation achieved the most potent in vivo antitumor efficacy across multiple dimensional readouts. These results indicate that biomimetic membrane-mediated homotypic targeting and penetrating peptide-facilitated tissue penetration exert synergistic effects, effectively enhancing intratumoral enrichment and deep penetration of nanocarriers following traversal of the blood–brain barrier (BBB). Animal experiments demonstrated that this system enhanced BBB penetration and intratumoral drug accumulation in the brain, with no apparent pathological damage observed in major organs or brain tissue [58].

#### 3.3.2. Ligand-Functionalized Enhancement Strategies

The targeting capacity of cell membrane coating alone depends on the membrane protein composition and is difficult to fine-tune for specific receptors. The introduction of targeting ligands, such as peptides and nucleic acid aptamers, can further improve tumor recognition specificity while preserving the advantages conferred by membrane coating.

In the domain of peptide ligands, Liu et al. developed a cyclic arginine-glycine-aspartic acid (cRGD) peptide-modified RBC membrane co-delivery system (siPFKFB4/PTX@RBCM-cRGD), which enhanced NCI-H446 cell uptake through cRGD-mediated active targeting. In vitro experiments showed that phosphofructo-2-kinase/fructose-2,6-bisphosphatase 4 (PFKFB4) knockdown reduced the half-maximal inhibitory concentration of PTX by approximately 51.5% and synergistically induced ferroptosis and immunogenic cell death [59]. Yan et al., targeting triple-negative breast cancer (TNBC), constructed functionalized nanoliposomes that enhanced the combined therapeutic effect through co-delivery of a chemotherapeutic agent and a specific miRNA targeting the *Slug* gene [60].

In the application of aptamers, Sun et al. developed EA2 aptamer-modified pH-sensitive liposomes that promote endocytosis through specific binding to α1-catenin (CTNNA1); the co-encapsulated luteolin not only alleviated hepatotoxicity in mice but also synergistically promoted apoptosis in esophageal squamous cell carcinoma cells with PTX [61]. Compared with peptide ligands, aptamers offer higher binding specificity, although their recognition efficiency in complex physiological environments such as blood or cerebrospinal fluid may be compromised by protein corona coverage.

In addition, Hu et al. used arginine-glycine-aspartic acid (RGD) peptide-modified nanocarriers to provoke local vascular inflammation in the tumor, which in turn promoted the accumulation of platelet membrane-coated drug-loaded platforms at the lesion site, thereby realizing an indirect targeting strategy [62]. This design, which couples targeting recognition with microenvironment modulation, provides a new design dimension for ligand functionalization.

#### 3.3.3. Multifunctional Hybrid Membrane Platforms

Hybrid membrane platforms, by fusing cell membranes from different sources, aim to integrate functions such as prolonged circulation, immune evasion, and tumor recognition within a single carrier.

To address heat shock protein 70 (HSP70)-associated chemoresistance in cervical cancer, Aili et al. developed a nanodelivery system (CuS-PIA@HR) coated with a fused RBC–HeLa cell membrane. Under near-infrared (NIR) light triggering, this system generated nitric oxide, downregulated HSP70 expression, and attenuated the chemoprotective mechanisms of tumor cells, achieving a cell death rate of 99.6% in vitro [63].

For brain-targeted delivery, Zhou et al. developed a liposomal delivery system (PTX@C-MMCL) incorporating macrophage membrane and U87 glioma cell membrane fused at a 1:1 ratio. The macrophage membrane conferred responsiveness to inflammatory chemokines to facilitate BBB penetration, while the U87 cell membrane enhanced homologous tumor targeting within the brain. Animal experiments showed that this hybrid carrier prolonged the blood half-life of PTX to 26.6 h and markedly increased drug accumulation at the tumor site [64].

#### 3.3.4. Clinical Translation Challenges

Although cell membrane-coated and ligand-functionalized systems have shown certain advantages in animal studies, their clinical translation still confronts multiple pharmaceutics-related challenges. During membrane fusion and coating processes, membrane-associated proteins may undergo structural alteration due to physical or chemical treatment, leading to diminished targeting function or biological activity [65]. In addition, cell membrane source, membrane protein integrity, batch-to-batch consistency, sterilization methods, and storage stability can all affect formulation quality. Although RBC membranes have demonstrated favorable long-circulation performance in animal models [66], their clinical application still requires further resolution of issues pertaining to membrane integrity maintenance, immune compatibility, and scalable manufacturing. Overall, biomimetic membrane and active targeting strategies offer new design directions for improving the in vivo distribution of PTX, but their advantages still require further validation in models closer to the clinical setting and in standardized manufacturing systems.

### 3.4. TME Matrix Remodeling Strategies

The biomimetic membrane and active targeting strategies discussed above focus primarily on enhancing the specific recognition and uptake of nanocarriers by tumor cells. However, before a delivery system can actually contact and act upon its target cells, it must first traverse the complex physical defenses erected by the solid tumor microenvironment. Abnormally deposited ECM components in solid tumors, notably HA and collagen, constitute the core physical barrier impeding deep tumor penetration of PTX nanomedicines. The dense matrix not only compresses blood and lymphatic vessels, leading to elevated interstitial fluid pressure (IFP) that directly restricts convective and diffusive drug transport, but also enhances tissue stiffness, which can markedly reduce delivery efficiency through physical impedance. Researchers have therefore explored a variety of strategies for actively degrading or remodeling the tumor matrix to improve the tissue penetration of PTX.

The direct enzymatic degradation of highly accumulated HA in the tumor matrix represents one of the earlier directions to enter clinical investigation. A randomized phase II trial (HALO109-202) in metastatic pancreatic cancer evaluated the combination of PEGPH20 with nab-paclitaxel and gemcitabine. The results demonstrated significant survival benefit in the patient subgroup exhibiting high type III collagen turnover (C3M/PRO-C3 ratio) at baseline [67]. This finding suggests that enzymatic degradation of the physical barrier can effectively enhance the penetration of nanoparticulate PTX; however, the efficacy of this strategy is highly dependent on the dynamic metabolic state of the patient’s tumor matrix.

Beyond enzymatic degradation targeting specific ECM components, physical approaches have also been combined with nanodelivery to address particularly dense and extreme physical barriers, such as the BBB in central nervous system tumors. A first-in-human phase I clinical trial demonstrated that an implantable low-intensity pulsed ultrasound device can safely and repeatedly open the BBB in the lesional regions of patients with recurrent glioblastoma, leading to a significant 3.7-fold increase in nab-paclitaxel concentration within the brain parenchyma [68]. It is important to note, however, that enhanced permeability of the physical barrier can simultaneously amplify the potential toxicity of the drug. A preclinical comparative study found that, under equivalent ultrasound conditions, the conventional Cremophor EL-containing PTX formulation caused severe intracerebral hemorrhage and demyelinating lesions, whereas nab-paclitaxel, from which the toxic solvent has been removed, exhibited favorable neural tolerability [69]. This result clearly indicates that when physical methods are employed to modulate microenvironmental barriers, they must be coupled with nanodelivery systems possessing excellent biocompatibility, so as to buffer the systemic toxicity that accompanies a sudden increase in barrier permeability.

Beyond the strategies of directly degrading the matrix or physically disrupting the barrier, the induction of tumor vascular “normalization” and active remodeling of the microenvironment through nanodelivery technology represents a distinct dimension of synergistic enhancement. In a phase I clinical trial of the AB160 nano-immunoconjugate, investigators non-covalently coated nab-paclitaxel nanoparticles with bevacizumab, constructing a delivery platform that combines targeting with microenvironment-modulating functions. This system achieved an objective response rate of 36.8% in heavily pretreated patients with advanced gynecologic malignancies [70]. Bevacizumab-mediated anti-vascular endothelial growth factor (VEGF) therapy not only endowed the carrier with targeting capability but also promoted transient normalization of tumor vasculature, thereby further reducing IFP and indirectly opening channels for deep penetration and sustained accumulation of PTX. Strategies that use specific pharmacological agents, such as vitamin D analogs, to revert activated pancreatic stellate cells to a quiescent state, thereby reducing collagen secretion at its source through a “matrix silencing” approach [71], also offer conceptually promising design avenues for future “matrix modulator–PTX” intelligent co-delivery systems.

### 3.5. Carrier-Free Prodrug Self-Assembly Delivery Systems

#### 3.5.1. Concept and Fundamentals of Self-Assembly Mechanisms

Nanocarriers such as polymeric micelles, liposomes, and biomimetic membrane-coated systems all require the introduction of substantial quantities of inactive excipients (excipient burden) when delivering PTX. This results in actual drug loading that is typically below 10% (*w*/*w*) [72], and is accompanied by carrier-associated systemic toxicity and potential immunogenicity. In response to these limitations, carrier-free prodrug self-assembly nanoassemblies have attracted considerable attention as an alternative formulation paradigm. This strategy employs rationally designed chemical linkers to covalently conjugate PTX with other functional molecules. The resulting prodrug molecules, through supramolecular driving forces including hydrophilic–lipophilic balance (HLB), π–π stacking, and molecular shape complementarity, spontaneously organize into ordered nanoassemblies, thereby achieving high-efficiency drug loading while markedly reducing excipient-related toxicity. During the self-assembly process, the amphiphilic character of the prodrug molecules and the intermolecular non-covalent interactions jointly determine the particle size, morphology, and colloidal stability of the assemblies, which in turn constitute the structural basis for subsequent TME-responsive drug release.

#### 3.5.2. TME Stimuli-Responsive PTX Single-Drug Self-Assemblies

To achieve precision drug release within the TME, researchers have undertaken systematic exploration of linker chemistry design. The classical redox-responsive strategy relies on the specific cleavage of disulfide bonds in the high-GSH environment of tumor cells. A recent subcellular pharmacokinetic (PK) study conducted a parallel comparison of prodrug nanoparticles constructed from tetraphenylporphyrin–PTX (TPP–PTX) conjugates bearing three different linkers: a thioether bond, a disulfide bond, and an inert two-carbon linker. By combining organelle fractionation with PK modeling, the study found that the endosomal and lysosomal systems are the primary sites of nanoparticle metabolism, with metabolic rates 10- to 27-fold higher than those in the cytosol; moreover, the transmembrane transport rate of nanoparticles (k[tr,NP] = 0.098 h^−1^) was far lower than that of free PTX (k[tr,PTX] = 27.1 h^−1^) [73]. These results suggest that disulfide-bond-based prodrug systems that partly depend on cytosolic GSH cleavage may be affected by endosomal/lysosomal entrapment, thereby limiting cytosolic drug release efficiency. To overcome the limitations of single-stimulus responsiveness, dual-stimulus responsive strategies have been successively proposed. For instance, the reductive responsiveness of a disulfide bond and the lipase responsiveness of a triglyceride ester bond, via adipose triglyceride lipase (ATGL), have been integrated within a single PTX prodrug molecule, with the triglyceride moiety serving a dual function as both a self-assembly template and a TME-responsive regulatory unit. Compared with a single GSH-responsive system, this dual reductive–enzymatic triggering design markedly enhanced intratumoral PTX release efficiency and antitumor activity [74]. In parallel, with regard to the optimization of prodrug molecular geometry, a systematic comparison of fatty alcohol modifications with varying chain lengths (AC12 to AC24) on the self-assembly behavior of PTX prodrugs revealed a trade-off between assembly stability and oxidative response sensitivity: an excessively short AC12 chain led to overly rapid drug release in the blood and consequent systemic toxicity, whereas an overly long AC24 chain inhibited intracellular PTX dissociation through excessive hydrophobic packing; the AC20 chain length achieved the optimal balance between efficacy and safety [75]. The above work provides critical experimental foundations for the rational design of linker arms and modification modules in single-drug prodrug systems.

#### 3.5.3. Synergistic/Dual-Drug Carrier-Free Co-Assembled Prodrugs

Building on single-drug prodrug platforms, multi-component carrier-free prodrugs covalently integrate different active components to maintain a fixed stoichiometric ratio at the molecular level, thereby reducing the loss of synergy that can arise from inconsistent component release rates in conventional physical co-encapsulation systems. Such systems are principally applied in two scenarios: first, the co-delivery of PTX with resistance-modulating agents, in which the simultaneous release of a chemotherapeutic and an efflux pump inhibitor, a mitochondrial modulator, or a DNA repair inhibitor enhances the killing of drug-resistant cells; and second, the integration of PTX with immunomodulatory or ferroptosis-inducing modules to simultaneously elicit cytotoxic effects and microenvironmental remodeling. For example, a three-component prodrug nanoassembly containing both disulfide and thioketal bonds can release PTX and a poly(ADP-ribose) polymerase (PARP) inhibitor under reductive and ROS-related conditions, respectively, and enhance synergistic antitumor efficacy in a drug-resistant non-small cell lung cancer (NSCLC) model through mitochondrial intervention [76]. Another class of PTX prodrugs exploits intracellular cysteine as a trigger for drug release while simultaneously depleting GSH reserves and promoting lipid peroxidation accumulation, thereby coupling chemotherapy with ferroptosis and immunomodulation. Overall, multi-component carrier-free prodrugs offer the advantages of high drug loading and fixed stoichiometric ratios; however, their structural complexity, release controllability, and batch-to-batch consistency remain problems that must be addressed for subsequent translation [77].

#### 3.5.4. Clinical Translation Challenges and Prospects

Carrier-free prodrug self-assembly systems still face several pharmaceutics-related obstacles in clinical translation. First, truly complete carrier-free formulations are difficult to realize in practice. Most systems require the introduction of agents such as distearoylphosphatidylethanolamine–PEG (DSPE–PEG) to maintain colloidal stability, which to some degree undercuts the conceptual advantage of being carrier-free and reintroduces excipient-associated risks [75,77]. Second, the assemblies encounter a blood dilution effect upon entering the systemic circulation: lacking the structural support of a polymer scaffold or lipid bilayer, they may fall below the critical aggregation concentration (CAC) upon extensive dilution and undergo irreversible disassembly, leading to premature drug leakage. Third, complex multi-step covalent syntheses and relatively broad polydispersity indices (PDI) constrain batch-to-batch reproducibility; for instance, three-component prodrugs involve multiple reaction steps, and β-cyclodextrin-grafted systems exhibit high PDI values [78]. Fourth, there exists an inherent tension between the high stability conferred by covalent conjugation and early-stage cytotoxic performance: the in vitro apoptosis rate induced by prodrug systems is lower than that of free drug combinations, suggesting that delayed drug release may attenuate the initial antitumor effect. Future research should seek a new structural design equilibrium between subcellular precision responsiveness and blood stability, and should develop simplified synthetic routes and self-stabilizing formulations.

## 4. Multi-Drug Co-Delivery Strategies for Reversing PTX Resistance

Unlike Section 3, which focused on how individual carriers respond to the tumor microenvironment (TME), this section addresses how multiple therapeutic components are synergistically organized within a single delivery system. For strategies that combine paclitaxel (PTX) with chemotherapeutic agents, efflux pump inhibitors, gene-silencing modules, or physical therapeutic units, efficacy depends not only on whether the drug reaches the tumor tissue, but also on whether the different components can maintain appropriate ratios in vivo, reach their respective intracellular compartments, and be released at the correct time. Accordingly, this section discusses the application logic and translational constraints of multi-drug co-delivery systems in mitigating PTX resistance across four dimensions: pharmacokinetic synchronization, subcellular spatial allocation, sequential release, and exogenous physical field triggering.

### 4.1. Fixed-Ratio Co-Delivery and Pharmacokinetic Synchronization

When PTX is used in combination with other chemotherapeutic agents or resistance sensitizers, therapeutic outcome depends not only on whether each drug reaches the tumor, but also on whether the different components can maintain ratios close to those identified as optimal during in vitro screening. Conventional physical mixing is prone to pharmacokinetic decoupling, driven by differences in molecular weight, lipophilicity, plasma protein binding, and clearance rate, which causes the actual drug ratio at the tumor site to deviate from the optimal synergistic ratio. The core objective of fixed-ratio co-delivery is therefore to minimize ratio drift among the different components during circulation, tissue distribution, and release, thereby improving the reproducibility of combination therapy.

The first category of strategy is the construction of heterodimers or dual-drug prodrugs through covalent linkage. Compared with free-drug mixtures, covalent conjugation maintains a fixed stoichiometric ratio at the molecular level and, to a meaningful degree, unifies the circulatory and tissue distribution profiles of the two agents. For example, PTX can be conjugated to the resistance sensitizer tetramethylpyrazine (TMP) via a reduction-responsive linker; the resulting dual-drug prodrug can self-assemble into nanoparticles and release both active components upon cleavage in the intracellular reducing environment [14]. Similarly, a dual-drug prodrug constructed from PTX and the vascular disrupting agent combretastatin A-4 (CA4) has demonstrated enhanced drug loading, reduced dependence on inert carrier materials, and preserved component ratios [79]. Covalent conjugation does, however, carry potential drawbacks, including synthetic complexity, uncertain metabolite safety, and the difficulty of balancing release kinetics.

The second category uses non-covalent structural confinement to maintain drug combination ratios. For example, Zou et al. reported a PTX/disulfiram (DSF) cocrystal nanosystem in which ordered molecular packing within the crystal lattice maintains a fixed ratio between the two drugs, with albumin anchoring and hyaluronic acid (HA) modification further enhancing tumor delivery. Exploiting the regulatory effect of DSF on *ABCB1*/*P-glycoprotein* (*P-gp*) expression, this system simultaneously preserves the PTX exposure ratio and sensitizes drug-resistant cells to chemotherapy [80].

**Figure 6:**
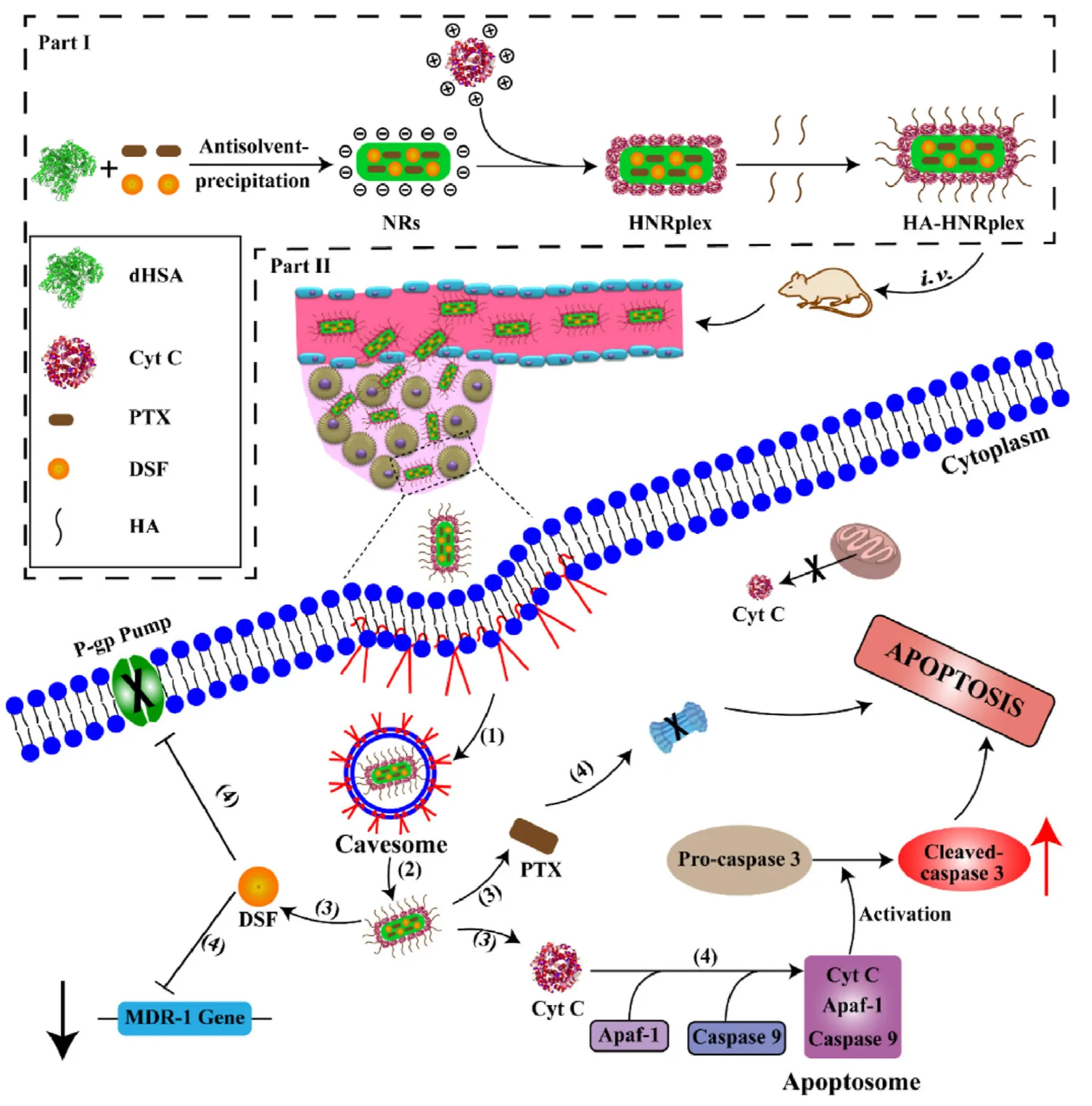
Schematic of the construction process and synergistic resistance-reversal mechanism of the HA-HNRplex three-component co-delivery system. This figure illustrates how hyaluronic acid (HA)-coated HNRplex (HA-HNRplex) integrates paclitaxel (PTX), disulfiram (DSF), and cytochrome c (Cyt C) within a single nanocomplex, and enhances effective drug exposure in drug-resistant tumor cells through a cascade process of “tumor enrichment → receptor-mediated endocytosis → cytosolic release → efflux inhibition and apoptosis amplification.” Part I shows the preparation process of HA-HNRplex: PTX and DSF first form PTX/DSF cocrystal nanorods (NRs) through anti-solvent precipitation; denatured human serum albumin (dHSA) is then anchored onto the NR surface and further combined with Cyt C to form HNRplex; finally, HA is coated onto the HNRplex surface to yield HA-HNRplex with cluster of differentiation 44 (CD44)-targeting -targeting potential. Part II shows the in vivo delivery fate and intracellular synergistic mechanism of HA-HNRplex: following intravenous (i.v.) injection, HA-HNRplex enriches in tumor tissue via the enhanced permeability and retention (EPR) effect, and HA–CD44 receptor interaction promotes tumor cell uptake with the formation of caveolae-associated vesicles (cavesomes); subsequently, cavesomes release HA-HNRplex into the tumor cell cytosol. Within the cytosol, HA-HNRplex releases PTX, DSF, and Cyt C, thereby exerting a “three-component synergistic” antitumor effect: DSF downregulates ATP-binding cassette subfamily B member 1 (*ABCB1*), historically termed multidrug resistance gene 1 (*MDR1*), and reduces *P-glycoprotein* (*P-gp*) expression, thereby inhibiting PTX efflux and increasing intracellular PTX exposure; PTX promotes tumor cell apoptosis by inhibiting microtubule depolymerization; Cyt C assembles with apoptotic protease activating factor 1 (Apaf-1) and caspase-9 to form the apoptosome, and promotes the cleavage of pro-caspase-3 to cleaved caspase-3, further amplifying the apoptosis execution process. Numbers (1)–(4) indicate the sequential events shown in Part II: (1) HA–CD44-mediated tumor-cell uptake and cavesome formation; (2) release of HA-HNRplex from cavesomes into the cytoplasm; (3) release of DSF, PTX, and Cyt C from HA-HNRplex; and (4) the downstream synergistic actions of the three components, including inhibition of *P*-gp-mediated drug efflux, PTX-induced microtubule stabilization, and Cyt C-mediated apoptosome/caspase-3 activation. The red Y-shaped membrane-associated symbols represent CD44 receptors involved in HA-mediated recognition and uptake. The red upward arrow adjacent to cleaved caspase-3 indicates increased caspase-3 cleavage/activation and enhanced apoptotic signaling. This system embodies the design logic of using co-delivery strategies to reverse PTX resistance through simultaneous efflux pump inhibition, enhanced intracellular PTX exposure, and activation of the mitochondrial apoptotic cascade. Reproduced from Ref. [80] under a CC BY-NC-ND 4.0 License.

As illustrated in Figure 6, cocrystal and other structural confinement approaches can reduce decoupling between different drugs during circulation and release compared with conventional physical co-encapsulation; however, their in vivo stability, polymorph consistency, and scalable manufacturing still require further validation.

The third category achieves ratiometric co-delivery through carrier-mediated spatial confinement. A lipidic lyotropic liquid crystal system, for example, exploits a three-dimensional lipid bilayer channel architecture to coordinate the release of hydrophilic and hydrophobic drugs, thereby reducing burst release and maintaining combined exposure [81]. In addition, prodrug modification to enhance the remote loading capacity of PTX in liposomes allows PTX and a second therapeutic agent to be distributed within the same vesicle at a preset ratio, providing an engineering solution for ratiometric liposomal co-delivery [82]. Overall, fixed-ratio co-delivery provides a more controllable pharmacokinetic foundation for PTX combination therapy than simple physical mixing; its clinical translation, however, depends on whether ratio maintenance can be verified in humans and whether complex formulations can be manufactured with scalable, quality-controllable processes.

Importantly, a fixed loading or stoichiometric ratio in the administered formulation does not by itself establish preservation of the same pharmacologically relevant ratio at the tumor site. Even when a carrier is designed for ratiometric co-delivery [79,80,81,82], differential release, carrier disassembly, linker cleavage, tissue transport, cellular uptake, and intracellular trafficking may alter the relative exposure of individual components after administration [83]. The resulting ratio may differ across plasma, the tumor interstitium, and different tumor-cell populations and therefore cannot be inferred solely from the initial formulation ratio. Translational evaluation should, where feasible, quantify time-resolved concentrations of carrier-associated and released components in plasma, tumor tissue, and relevant target cells.

### 4.2. Subcellular Compartment Targeting and Spatially Differentiated Co-Delivery

#### 4.2.1. Carrier Architecture-Driven Physical Spatial Partitioning

Building on the ratio-maintenance principles discussed in Section 4.1, co-delivery systems face an additional challenge upon cell entry: whether different therapeutic components can remain relatively segregated within the same carrier and subsequently enter their appropriate release pathways. For combinations of hydrophobic PTX with hydrophilic nucleic acids, proteins, or small-molecule sensitizers, physical spatial partitioning within the carrier can reduce inter-component interference and provide a structural foundation for downstream cytosolic release, membrane-region intervention, or gene silencing [83].

The core–shell structure is the prototypical form of physical spatial partitioning. Typically, hydrophobic PTX can be enriched within a polymeric or lipid core, while hydrophilic nucleic acid therapeutics or protein-based agents can be distributed in the outer layer or surface-modified regions, thereby achieving relative segregation within a single nanoparticle. Xu et al. used a glucosylceramide synthase (GCS) inhibitor as the active hydrophobic block to construct prodrug copolymer micelles, combining anticancer drug delivery with resistance-related pathway modulation [84]. Guo et al. introduced HA with CD44 receptor-targeting capability into the carrier outer layer, providing protection for the hydrophobic core while improving targeting recognition [85]. These studies suggest that core–shell partitioning serves not only to enhance colloidal stability but also to provide a structural basis for the functional allocation of multiple components.

As the precision of nanostructural design has improved, spatial partitioning has further extended from the core–shell level to molecular-scale positional assembly. Mesoporous silica nanoparticles (MSNs) and nucleic acid nanoframeworks, with their programmable modification sites, can be used to arrange different drugs at defined positions within a single carrier. Tarannum et al. exploited the differential functionalization of the internal and external surfaces of MSNs to achieve ratiometric spatial segregation of gemcitabine/cisplatin prodrugs [86]. Although this study did not employ a PTX model, its “internal–external surface partitioning” engineering logic can serve as a design reference for PTX co-delivery with nucleic acids or sensitizers. By contrast, Wang et al. site-specifically assembled PTX and miR-122 on multivalent RNA nanoparticles, achieving spatial co-localization of a chemotherapeutic agent and a gene-regulatory element, which is a more direct demonstration of the application value of PTX–nucleic acid co-delivery [87].

Thus, the significance of physical spatial partitioning should not be understood merely as co-loading or synchronous transport, but more substantively as the intracellular functional organization of multiple components. Wang et al., using framework-induced self-assembly to construct gene/PTX co-delivery nanocarriers, enabled *MDR1*-siRNA and PTX to act jointly in a drug-resistant tumor model, thereby enhancing the intervention against efflux pump-related resistance pathways [88].

#### 4.2.2. Spatial Intervention at the Plasma Membrane and Efflux Pump Network

In conventional multi-drug co-delivery system design, most nanocarriers first undergo endocytosis upon cell entry and are trafficked to vesicular compartments such as endosomes or lysosomes. However, drug efflux pumps such as *P-gp* are predominantly localized to the plasma membrane and its associated lipid microdomains; drugs or sensitizers released post-endocytosis may not achieve sufficient exposure in efflux pump-enriched regions. Wang et al. reported pH-sensitive polymeric micelles co-delivering PTX and honokiol that concurrently suppressed multidrug resistance (MDR) and metastasis in breast cancer [89]. Studies of this kind highlight the need, when designing PTX co-delivery systems, to consider the spatial match between the intracellular release site of the drug and the plasma membrane localization of *P-gp*.

Membrane anchoring strategies represent one engineering approach for improving local exposure at the plasma membrane. By introducing hydrophobic protrusions or lipophilic ligands onto the nanoparticle surface, the interfacial interaction between the carrier and the phospholipid bilayer can be enhanced, thereby prolonging the residence time in the vicinity of the cell membrane. The study by Xia et al. on hydrophobic protrusion-enhanced membrane anchoring and cellular internalization is not a PTX co-delivery model, but it demonstrates that surface topology can influence the local interaction between nanoparticles and the plasma membrane [90]. If this concept is combined with PTX/*P-gp* inhibitor or PTX/photosensitizer co-delivery, it may help improve membrane-region intervention efficiency.

Local plasma membrane intervention can also be combined with photodynamic therapy (PDT). The reactive oxygen species (ROS) generated by a photosensitizer at a specific location have a limited radius of action; photosensitizer subcellular localization therefore directly affects therapeutic outcome. Fan et al. constructed a plasma membrane-targeted PDT system demonstrating that membrane-region ROS can induce localized oxidative stress and influence the therapeutic response of hypoxic tumors [91]. In PTX-related systems, Chang et al. used porphyrin-lipid-stabilized PTX nanoemulsion to integrate PDT and chemotherapy within a single nanoplatform, achieving enhanced tumor cell killing upon light irradiation [92]. Another PTX-potentiated photodynamic theranostic system also showed synergistic chemo-PDT potential [93]. However, the direct effects of such systems on *P-gp* function or MDR reversal should be stated cautiously unless efflux pump expression, drug efflux, or resistance indices were explicitly measured in the original studies.

#### 4.2.3. Mitochondrial, Lysosomal, and Autophagy Network Intervention

Beyond efflux pump intervention at the plasma membrane level, another category of spatially differentiated strategies focuses on mitochondria, lysosomes, and the autophagy network. These strategies do not necessarily block *P-gp* membrane localization directly; rather, they diminish the metabolic foundation that drug-resistant cells require to sustain an efflux-competent phenotype and evade apoptosis by depleting adenosine triphosphate (ATP) supply, disrupting redox buffering, or suppressing protective autophagy. Lin et al. constructed folate-modified, redox-responsive mixed micelles for PTX delivery; in PTX-resistant breast cancer cells, these micelles reduced ATP-binding cassette (ABC) transporter-mediated drug efflux by interfering with mitochondrial function and ATP synthesis, thereby increasing intracellular PTX accumulation [94].

Building on efflux restriction, the mitochondrion also serves as the key node at which PTX combination therapy transitions from “drug accumulation” to “apoptosis execution.” Drug-resistant tumor cells often buffer chemotherapy-induced death signals by maintaining mitochondrial membrane potential, enhancing antioxidant capacity, and upregulating anti-apoptotic proteins. A recent study targeting PTX-resistant non-small cell lung cancer (NSCLC) constructed a glutathione (GSH)/ROS-responsive multi-component prodrug nanosystem integrating PTX, PARP inhibition, and mitochondrial metabolic intervention within a single platform; through the synergistic effects of microtubule disruption, DNA repair inhibition, and mitochondrial dysfunction, this system enhanced apoptosis in drug-resistant cells [76].

The lysosomal and autophagy systems also influence whether a co-delivery system can convert cellular uptake into effective cytosolic exposure. Following endocytic entry, if nucleic acid therapeutics remain trapped for extended periods in acidic vesicles, siRNA or miRNA cannot reach the cytosol to accomplish target gene silencing; similarly, if PTX is retained for prolonged periods within vesicular compartments, its effective concentration at microtubule-related targets is reduced. Concurrently, protective autophagy can help drug-resistant cells clear damaged mitochondria and protein aggregates, thereby attenuating chemotherapy-induced apoptotic responses. A study on PTX resistance in ovarian cancer showed that FDX1 can drive PTX resistance through copper metabolism and the ULK1/ATG13-mediated autophagy axis, while pH/ROS-responsive si-FDX1 nanomicelles can release siRNA, inhibit the relevant autophagy-mediated resistance pathway, and enhance PTX therapeutic sensitivity [95]. Another carrier-free drug–chemogene conjugate strategy suggests that reducing inert carrier material and increasing the effective payload ratio may help improve the intracellular release efficiency of PTX–nucleic acid synergistic systems [96].

Overall, mitochondria, lysosomes, and the autophagy system are not merely passive intracellular transport backgrounds but important regulatory nodes that determine whether a PTX co-delivery system can achieve drug accumulation, cytosolic release, and apoptosis execution. By disrupting energy supply, inducing mitochondrial stress, or relieving endosomal/lysosomal entrapment, subcellular organelle-targeting strategies can further convert the delivery advantages of PTX into therapeutic sensitization effects.

#### 4.2.4. Nuclear Targeting and Genomic-Level Synergy

A further spatial delivery concept is nuclear targeting. Compared with cytosolic or organellar delivery, intranuclear delivery is constrained by the size-exclusion barrier imposed by the nuclear envelope and nuclear pore complex; the key is therefore not simply to increase total intracellular drug quantity, but to enhance the probability of therapeutic molecules reaching the perinuclear or intranuclear region through cell-penetrating peptide exposure, perinuclear enrichment, or nuclear membrane perturbation. Cao et al. constructed an extracellular pH (pHe)/photo dual-sensitive polymeric nanocarrier that exposes the TAT cell-penetrating peptide in an acidic environment and generates ROS under light irradiation to promote intranuclear release [97]. In addition, double-imprinted nanoparticles have demonstrated the potential for continuous spatial transport from cell membrane recognition to intranuclear delivery [98].

Compared with plasma membrane and organelle targeting, nuclear-targeted delivery is relevant to resistance research because it may intervene in the long-term resistance phenotype of tumor cells at the transcriptional and epigenetic levels. PTX resistance is not determined solely by *P-gp* efflux or microtubule target alterations; non-coding RNA networks, promoter methylation, and aberrant transcription factor activation can also sustain anti-apoptotic states, cell-cycle escape, and drug insensitivity. A nuclear-targeted siRNA delivery study, in which gold nanoparticles were simultaneously modified with siRNA and a nuclear localization signal (NLS) peptide, directed siRNA to promoter regions and induced RNA-directed DNA methylation, achieving prolonged gene silencing [99]. Another study using NLS peptide-modified gold nanoparticles to deliver AS1411 and anti-miR-221 demonstrated that nuclear-targeted nanosystems can modulate malignant phenotypes through epigenetics-related pathways [100].

In genomic-level combination therapy, intranuclear delivery can further enhance the sensitivity of drug-resistant cells to chemotherapeutic stress by amplifying DNA damage or weakening the DNA damage response (DDR). The canonical action of PTX is to stabilize microtubules and induce G_2_/M phase arrest, but drug-resistant cells may reduce the killing effect through checkpoint adaptation, enhanced DNA repair, or aberrant mitotic exit. Thus, if PTX-induced cell-cycle stress is superimposed with intranuclear DNA damage, replication stress, or repair inhibition, it may, in principle, further compromise the ability of drug-resistant cells to maintain genomic stability. Cai et al. constructed a supramolecular “Trojan horse” system that enhanced intranuclear drug accumulation and DNA-related damage through nuclear delivery of dual anticancer agents, providing a model-based rationale for intranuclear synergistic therapy [101]. Existing evidence, however, primarily supports the feasibility of nuclear-targeted delivery; further validation in PTX-resistance models is needed to assess its genuine translational value.

### 4.3. Cascade-Responsive and Sequential Release Strategies

Whereas Section 3 focused on how individual carriers respond to the TME, this section addresses the issue of release sequencing in multi-component systems. For combinations of PTX with sensitizers, matrix modulators, or gene-silencing modules, efficacy depends not only on whether the components reach the tumor tissue, but also on whether the modulatory component can first relieve the corresponding barrier before PTX exerts its cytotoxic effect. The core of cascade delivery is therefore not the simple addition of pH-, enzyme-, or GSH-responsive elements, but the exploitation of these biochemical gradients to orchestrate a spatiotemporal sequence of “barrier removal first, main drug release second”.

#### 4.3.1. Stepwise Penetration of Multiple TME Barriers

In PTX co-delivery systems, common sequential release logic can be distilled into three layers: first, improving tumor uptake; second, enhancing tissue penetration; and third, relieving intracellular efflux or resistance pathways.

The first category of strategy addresses the conflict between circulatory stability and tumor uptake. Nanomedicines generally require a relatively stable or negatively charged surface in the bloodstream to reduce non-specific clearance; however, upon entering the mildly acidic TME, a negatively charged surface may restrict binding to the cell membrane. Acid-sensitive groups such as 2,3-dimethylmaleic anhydride (DMA) can mask positive charges at neutral pH, then hydrolyze and expose cationic amino groups in the mildly acidic tumor environment, thereby promoting cellular uptake. Chen et al. demonstrated that a pH shift from 7.4 to 6.5 could drive nanoparticle ζ-potential from negative to positive [102]. Building on a similar logic, Zheng et al. constructed dual-pH-sensitive charge-reversal micelles co-loaded with PTX and calcitriol, achieving a cascade of circulatory stability, tumor uptake, and intracellular drug release in a 4T1 triple-negative breast cancer (TNBC) model; calcitriol-mediated modulation of MMP-9, BCL-2, and E-cadherin further mitigated the pro-metastatic effects potentially induced by PTX monotherapy [103].

The second category targets the insufficient deep penetration caused by dense extracellular matrix (ECM) and elevated interstitial fluid pressure. Cancer-associated fibroblast (CAF) activation promotes collagen and HA deposition, restricting nanocarrier diffusion into deep tumor regions [104]. Multi-component systems can therefore adopt a “matrix modulation first, PTX release second” design. Yin et al. developed a hierarchical nanosystem co-loaded with PTX and pH-activated hyaluronidase (PEGPH20), in which the enzyme module degrades HA in the mildly acidic tumor environment to improve penetration, after which PTX is released under the synergistic action of the intracellular acidic environment and high GSH levels [105]. Fang et al. used MMP-2-triggered telmisartan release to reduce ECM production through modulation of transforming growth factor-β (TGF-β)-related signaling, subsequently promoting PTX arrival in deep tumor regions [106]. These two approaches, respectively, represent the pathways of “degrading existing matrix” and “suppressing de novo matrix production”.

**Figure 7:**
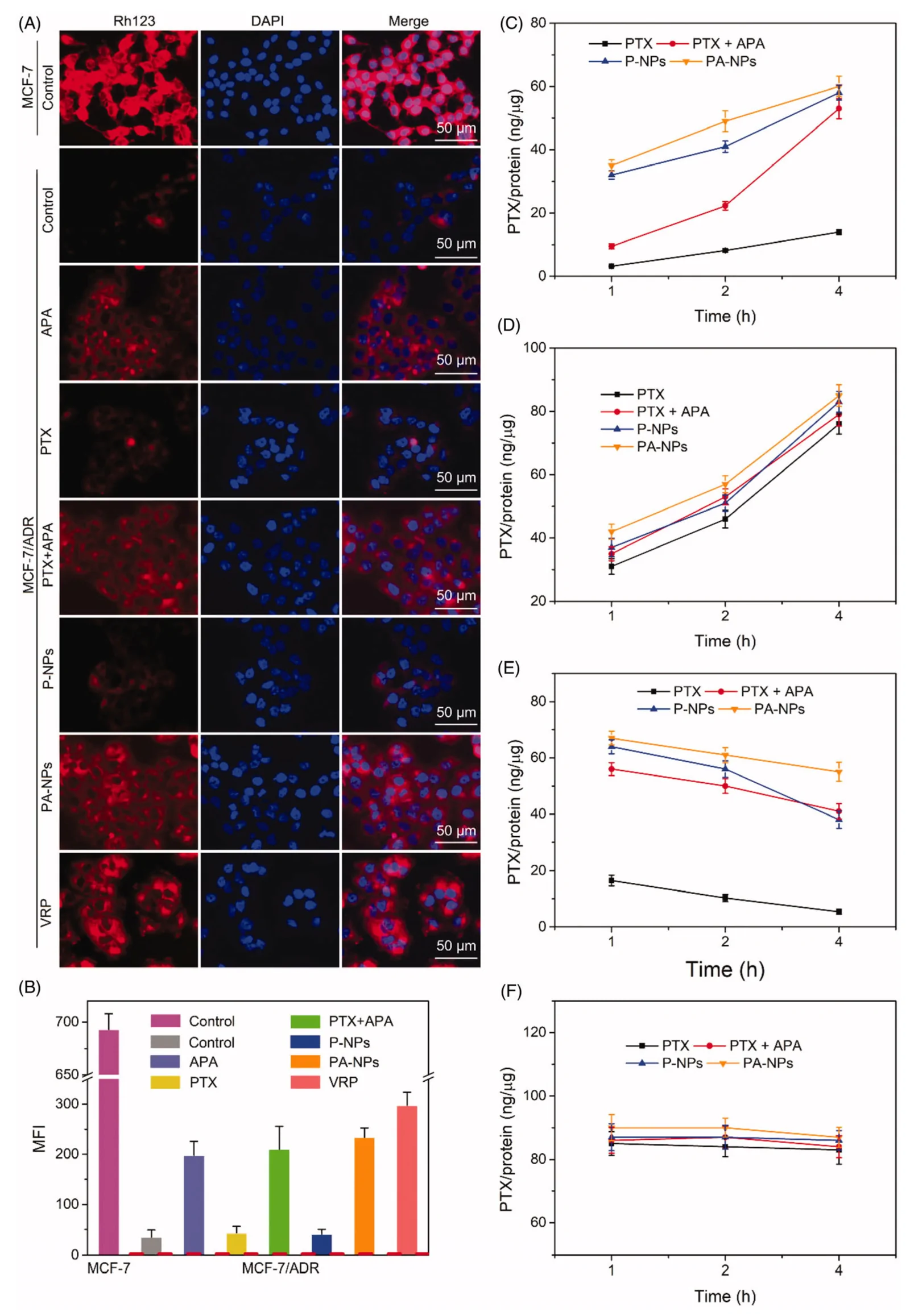
Experimental evidence for apatinib (APA) co-delivery-enhanced drug accumulation and efflux inhibition in drug-resistant cells. This figure illustrates the effect of apatinib co-delivery with paclitaxel (PTX) on enhancing intracellular drug exposure and reversing efflux-related resistance in drug-resistant cells with high *P-glycoprotein* (*P-gp*) expression. Rhodamine 123 (Rh123) is a classical fluorescent *P-gp* substrate; its intracellular fluorescence intensity reflects *P-gp*-mediated drug efflux function. (**A**) Rh123 uptake/retention in MCF-7 sensitive cells and MCF-7/ADR drug-resistant cells under different treatment conditions. Cells were pre-treated with phosphate-buffered saline (PBS, control), APA, PTX, PTX + APA, PTX-loaded polymeric micelles (P-NPs), PTX/APA co-loaded polymeric micelles (PA-NPs), or verapamil (VRP, *P-gp* inhibitor positive control) for 3 h, followed by Rh123 incubation for 4 h; 4′,6-diamidino-2-phenylindole (DAPI) was used for nuclear staining, and Merge represents the overlay of Rh123 and DAPI signals. (**B**) Corresponding flow cytometry quantification, with mean fluorescence intensity (MFI) representing intracellular Rh123 retention (*n* = 6). The red dashed horizontal line along the baseline denotes the zero-MFI reference level and does not represent an additional experimental group or statistical comparison. (**C**,**D**) PTX accumulation assay: MCF-7/ADR cells (**C**) and MCF-7 cells (**D**) were treated with PTX, PTX + APA, P-NPs, or PA-NPs for 1, 2, and 4 h; intracellular PTX content was determined by high-performance liquid chromatography (HPLC) and normalized to cellular protein content as ng PTX/μg protein (*n* = 6). (**E**,**F**) PTX efflux assay: cells were pre-incubated with the above formulations for 4 h, then transferred to drug-free medium, and residual intracellular PTX content was measured at 1, 2, and 4 h (ng PTX/μg protein) (*n* = 6). The results show that, compared with free PTX or PTX-only P-NPs, PA-NPs markedly enhanced the Rh123 fluorescence signal, increased intracellular PTX accumulation, and slowed PTX efflux in MCF-7/ADR cells; in MCF-7 sensitive cells, inter-group differences were relatively small. These findings demonstrate that APA co-delivery can increase effective PTX exposure in drug-resistant cells by inhibiting *P-gp*-related efflux function, thereby providing an experimental basis for reversing multidrug resistance (MDR). Reproduced from Ref. [107] under a CC BY 4.0 License.

The third category addresses the insufficient intracellular PTX exposure caused by *P-gp*-mediated efflux. Even if the carrier accomplishes tissue penetration, PTX entering the cytosol can still be effluxed by *P-gp* in an ATP-dependent manner. To raise the effective intracellular concentration, co-delivery systems can first release an efflux pump inhibitor or gene-silencing module, followed by PTX. Zhang et al. used hyaluronic acid–cystamine–polylysine micelles co-loaded with PTX and apatinib; upon intracellular GSH triggering, apatinib was released to inhibit *P-gp* ATPase activity, subsequently promoting PTX accumulation and enhancing cytotoxicity against MCF-7/ADR cells [107]. As illustrated in Figure 7, the intervention of this system in efflux-related processes can be reflected by enhanced rhodamine 123 (Rh123) uptake/retention and increased intracellular PTX accumulation accompanied by altered efflux kinetics. Jia et al. constructed a redox/pH-responsive nanosystem co-loaded with *P-gp* short hairpin RNA (shRNA) and PTX, which first downregulated *P-gp* expression and then promoted PTX release, demonstrating a substantial resistance-reversal capacity [108]. The framework-induced self-assembly system of Wang et al., co-loaded with *MDR1*-siRNA and PTX, further supports the feasibility of combining gene silencing with PTX chemotherapy [88].

A common feature of the above strategies is the exploitation of pH, enzyme, and GSH gradients between tumor and normal tissues to program the release sequence. It should be noted, however, that these gradients are not stably present across all tumors or patients, and their spatial distribution exhibits considerable heterogeneity. TME signals are thus more appropriately regarded as exploitable but not entirely reliable trigger conditions, rather than as absolutely precise “intrinsic signposts”.

#### 4.3.2. Evidence for Synergistic Enhancement by Sequential Release

The release sequence itself can directly alter the pharmacodynamic output of combination therapy. Gregory et al., using bicompartmental Janus nanoparticles, compared the release kinetics of PTX and lapatinib: simultaneous administration produced no significant synergy, whereas administering lapatinib approximately 4 h before PTX reduced the combination index (CI) to approximately 0.5, indicating that the same drug combination can exhibit qualitatively different levels of synergy depending on the release sequence [109]. In addition, Yu et al., by interfering with CAF metabolism to reduce ECM production before releasing the chemotherapeutic agent, provided evidence that prior alleviation of the matrix barrier can enhance drug penetration [110]. Guo et al., using quercetin to first downregulate *P-gp* expression before releasing PTX, likewise support the “resistance relief first, chemotherapy second” temporal logic [111]. The key to sequential release design therefore lies not in simply adding more response nodes, but in determining the minimum effective onset time of each modulatory module and defining the PTX release window accordingly.

#### 4.3.3. Clinical Translation Status and Optimization Directions

Although cascade and sequential release strategies are attractive in preclinical models, the clinical translation of nanomedicines remains constrained by delivery efficiency and in vivo predictability. A meta-analysis by Chen et al., encompassing 297 studies and 534 datasets, indicated that the median tumor delivery efficiency of intravenously injected nanoparticles in solid tumors is approximately 0.67% of the injected dose (%ID), with reticuloendothelial system organs such as the liver and spleen often showing higher accumulation levels; tumor delivery efficiency also declines over time [10]. Concurrently, the enhanced permeability and retention (EPR) effect exhibits significant inter-individual variability in clinical patients, and its performance in human tumors is generally weaker than that observed in standard mouse subcutaneous xenograft models. Pallares et al. have also noted that the clinical contribution of many approved nanomedicines is primarily reflected in improved tolerability rather than significantly enhanced efficacy [9]. For PTX co-delivery systems, this means that even if the in vitro release sequence is rationally designed, the actual drug exposure reaching the tumor may still fall below the threshold required to produce a synergistic effect.

Future PTX co-delivery systems should therefore not simply pursue the accretion of additional functionalities, but should instead seek a balance among complexity, manufacturability, and therapeutic benefit. First, the number of response modules should be minimized, with priority given to trigger nodes directly relevant to the therapeutic mechanism, such as pH/GSH or enzyme/GSH combinations. Second, TME imaging or biomarker detection may be integrated to stratify patients according to tumor acidity, enzyme expression, or matrix characteristics, thereby improving the rationality of trigger threshold and release window design. Third, carrier scaffolds should preferentially employ materials with an existing clinical application foundation, such as poly(lactic-co-glycolic acid) (PLGA), PEG, albumin, or lipid-based systems, to reduce the difficulty of manufacturing scale-up and quality control. For a drug such as PTX with a relatively narrow therapeutic window, preclinical evaluation should not be limited to reporting in vitro cytotoxicity or tumor volume inhibition rates, but should also include intratumoral drug ratios, release kinetics, resistance biomarker changes, and long-term safety. Representative experimental paclitaxel (PTX) nanodelivery platforms and their principal translational attributes are summarized in Table 1. Figure 4 provides a complementary schematic of the stage-dependent size–charge transition mechanism.

### 4.4. Exogenous Physical Field-Triggered PTX Multimodal Combination Delivery

Endogenous microenvironment-triggered strategies are susceptible to heterogeneity in tumor acidity, enzyme expression, redox status, and blood perfusion. By contrast, exogenous physical fields, such as near-infrared (NIR) light, ultrasound, and alternating magnetic fields (AMF), can, to a degree, provide more controllable spatiotemporal trigger signals and combine with PTX chemotherapy to form hyperthermia-, photodynamic-, sonodynamic-, or magnetothermal-based combination treatments. It is important to emphasize that the strategies described in this section are more accurately classified as PTX multimodal combination delivery; not all examples directly demonstrate MDR reversal. Their potential sensitization mechanisms primarily include promoting drug release, enhancing intracellular uptake, inducing ROS or thermal damage, and disrupting drug-resistant cell homeostasis.

In photothermal therapy (PTT) systems, photothermal conversion materials convert NIR light into localized thermal energy, thereby promoting PTX release and enhancing tumor cell damage. Lee et al. used MXene to construct an HA–PLGA nanoplatform; under 808 nm laser irradiation, the photothermal effect promoted polymeric matrix dissociation and PTX release, achieving synergistic chemo-PTT [112]. Liu et al. introduced the phase-change material tetradecanol into a mesoporous Prussian blue system: at physiological temperature, the solid phase-change material seals the mesopores to reduce PTX leakage, while NIR irradiation-induced temperature elevation triggers a phase transition that initiates PTX release [113]. More broadly, advances in semiconductor photonic and biointegrated device architectures have expanded the technological basis for externally controlled biomedical stimulation and drug release [114]; however, optical applicability remains constrained by light penetration depth, irradiation safety, and target accessibility.

Beyond thermal effects, PDT relies primarily on photosensitizer-generated ROS upon light irradiation. ROS can directly cause oxidative damage and can also cleave ROS-sensitive linkers to trigger PTX prodrug release. Zhang et al. designed a carrier-free prodrug nanoplatform in which a ROS-sensitive PTX prodrug and a porphyrin prodrug are co-assembled; upon light irradiation, the generated ROS simultaneously participate in PDT and PTX prodrug cleavage, yielding chemo-photodynamic synergy [115]. Zhou et al. used the oxygen depletion associated with PDT to activate an azobenzene-linked hypoxia-responsive PTX dimer prodrug, so that PDT-induced hypoxia promoted subsequent drug release [116]. Such designs exemplify the coupling of exogenous optical fields with endogenous chemical responsiveness, but are sensitive to oxygen availability, irradiation depth, and tumor perfusion conditions.

Because first-window near-infrared (NIR-I) light remains subject to scattering and absorption limitations in tissue, second-window near-infrared (NIR-II) light and ultrasound have been explored for triggering at greater depths. He et al. used apoferritin nanocages co-loaded with PTX and the NIR-II dye IR1061, achieving photothermally triggered PTX release and synergistic therapy under 1064 nm laser irradiation [117,118]. These strategies offer avenues for deep-seated tumor treatment, although sonication parameters, energy deposition, and normal tissue safety still require evaluation.

Alternating magnetic fields (AMFs) are less affected by tissue light scattering and absorption and can be used for magnetothermal–chemotherapy combination triggering, though their effectiveness remains subject to constraints imposed by field strength, frequency, material thermal conversion efficiency, and safety thresholds. Tavakoli et al. designed a platelet membrane-camouflaged superparamagnetic iron oxide nanoparticle (SPION) drug-loaded system that produced a localized magnetothermal effect under AMF and synergized with PTX chemotherapy [119]. The value of magnetically responsive systems should thus be understood as that of an exogenous field strategy relatively suited to deep-trigger applications, rather than being characterized in terms of “near-infinite penetration”.

Overall, exogenous physical fields endow PTX combination therapy with enhanced spatiotemporal controllability, and can promote drug release and tumor cell damage through localized hyperthermia, ROS generation, phase-transition triggering, or magnetothermal effects. The clinical translation of such systems, however, must still contend with issues of energy deposition safety, target-region accessibility, carrier batch-to-batch consistency, and insufficient real drug exposure in deep-seated tumors.

In conclusion, the design logic of PTX multi-drug co-delivery systems is transitioning from simple “co-loading” toward multi-dimensional coordination of ratio, space, and time. Fixed-ratio delivery helps to alleviate pharmacokinetic decoupling in combination regimens; subcellular spatial partitioning can enhance the efficiency of intervention against efflux pumps, metabolic homeostasis, and gene regulatory networks; sequential release and exogenous physical field triggering further enhance the programmability of the release process. Whether these advantages can be translated into clinical benefit, however, still depends on in vivo delivery efficiency, drug exposure thresholds, manufacturing reproducibility, and safety margins. Future research should move beyond relying primarily on in vitro cytotoxicity or short-term tumor volume inhibition rates as evidence, and should instead report intratumoral drug ratios, release kinetics, resistance biomarker changes, long-term safety, and survival benefit.

## 5. Clinical Translation Progress and Challenges of Novel PTX Delivery Systems

The preceding sections have systematically surveyed progress at the laboratory-level across stimuli-responsive delivery, biomimetic targeting, carrier-free prodrug self-assembly, and multi-drug co-delivery strategies. Between mechanistic investigation and clinical benefit, however, a translational gap persists. This section focuses on that gap. Section 5.1 reviews the current clinical application status and limitations of approved paclitaxel (PTX) nanoformulations as a benchmark reference; Section 5.2 surveys the clinical research progress of next-generation delivery systems; Section 5.3 systematically analyzes the deep-seated challenges constraining clinical translation; and Section 5.4 discusses the auxiliary role of computational methods and artificial intelligence (AI) in formulation optimization, translational evaluation, and patient stratification.

### 5.1. Clinical Application Status of Approved PTX Nanoformulations

Clinically established PTX nanoformulations can be grouped by carrier type: nab-paclitaxel (Abraxane), which uses human serum albumin as the carrier, and liposomal or synthetic-polymer formulations such as Lipusu and Genexol-PM. Paclical, marketed in the European Union (EU) as Apealea, also used a synthetic micellar excipient; however, its EU marketing authorization was withdrawn on 9 February 2024 at the holder’s request for commercial reasons and is therefore discussed here as a historical regulatory example [120]. The shared point of departure for these formulations is the elimination of the safety limitations imposed by the Cremophor EL (CrEL)/dehydrated ethanol co-solvent system used in the conventional Taxol formulation (see Section 2.2 for the mechanisms of CrEL-related toxicity). This section focuses on the clinical approval basis, real-world application status, and clinical limitations of these formulations, thereby providing a benchmark against which the progress of next-generation delivery systems can be assessed.

#### 5.1.1. Albumin-Bound PTX (Nab-Paclitaxel, Abraxane)

Nab-paclitaxel uses human serum albumin (HSA) as a natural carrier and is manufactured through a high-pressure homogenization process, yielding albumin-bound nanoparticles with a mean diameter of approximately 130 nm. This CrEL-free formulation not only circumvents co-solvent toxicity but also reduces the clinical infusion time from 3 h to 30 min and, in most clinical settings, eliminates the need for routine corticosteroid premedication.

As the first albumin-based nanoparticulate PTX formulation to achieve successful clinical translation and broad regulatory approval, nab-paclitaxel has been widely adopted in the treatment of solid tumors including breast cancer, non-small cell lung cancer (NSCLC), and pancreatic cancer. The formulation received U.S. Food and Drug Administration (FDA) approval in 2005 for second-line treatment of metastatic breast cancer; subsequent approvals followed in 2012 for first-line treatment of locally advanced or metastatic NSCLC in combination with carboplatin, and in 2013 for first-line treatment of metastatic pancreatic adenocarcinoma in combination with gemcitabine [121]. Overall, the successful translation of nab-paclitaxel derives primarily from its direct resolution of a tangible clinical problem, CrEL-related toxicity and administration constraints, rather than from reliance on complex molecular targeting functions. This provides an important industry lesson for the clinical translation of subsequent nanomedicines.

With respect to the tumor enrichment mechanism, nab-paclitaxel principally depends on gp60/caveolin-1-mediated transcytosis to facilitate active nanoparticle traversal of the endothelial barrier. Upon entry into the tumor microenvironment (TME), the interaction between albumin and secreted protein acidic and rich in cysteine (SPARC) was once hypothesized to promote local PTX retention; however, this hypothesis was not validated in large-scale phase III clinical trials, no significant correlation was found between high SPARC expression and nab-paclitaxel efficacy, and SPARC is currently not recommended as a predictive biomarker [122]. Nevertheless, the aforementioned enrichment mechanisms still enable nab-paclitaxel to maintain a relatively manageable safety profile at higher dose intensities, and head-to-head studies have shown superior objective response rates compared with conventional solvent-based PTX [123].

The elimination of CrEL also confers an important compatibility advantage for nab-paclitaxel in combination regimens with immune checkpoint inhibitors (ICIs). The dexamethasone premedication required for conventional solvent-based PTX may adversely affect the immune microenvironment and thereby interfere with the antitumor activity of ICIs. In triple-negative breast cancer (TNBC), the IMpassion130 trial demonstrated that atezolizumab combined with nab-paclitaxel significantly prolonged progression-free survival (PFS) in patients positive for programmed death-ligand 1 (PD-L1); by contrast, in the similarly designed IMpassion131 trial, substitution of nab-paclitaxel with conventional solvent-based PTX failed to replicate comparable survival benefit in the intention-to-treat (ITT) population and subgroups [124]. Although the discrepancy between the two trials cannot be fully attributed to corticosteroid premedication, it strongly highlights the impact of chemotherapy backbone choice on the clinical response to ICI combination therapy.

Against the backdrop of immunotherapy and multimodal combination therapy increasingly becoming mainstream strategies for solid tumors, nab-paclitaxel, by virtue of its dosing compatibility and non-immunosuppressive characteristics in the microenvironment, has become a commonly used taxane backbone in many combination regimens. Multiple phase III clinical studies are currently further evaluating the clinical value of nab-paclitaxel in combination with ICIs, tumor-treating fields (TTFields), and targeted therapy. For instance, the PANOVA-3 study is assessing the therapeutic potential of TTFields combined with gemcitabine/nab-paclitaxel in locally advanced pancreatic cancer; the ROSELLA trial is evaluating the combination strategy of nab-paclitaxel with the glucocorticoid receptor antagonist relacorilant in platinum-resistant ovarian cancer [125].

#### 5.1.2. Liposomal and Polymeric Micelle Formulations (Lipusu/Genexol-PM/Paclical)

Available clinical studies provide a degree of support for the safety and usability of these formulations. In a phase III study enrolling 540 patients with locally advanced or metastatic lung squamous cell carcinoma, Lipusu combined with cisplatin showed comparable efficacy to gemcitabine plus cisplatin, with lower rates of treatment discontinuation and reduced hematologic toxicity [126]. Paclical replaces CrEL with the retinoid derivative XR-17 as a micelle-forming excipient; in a phase III trial in recurrent platinum-sensitive ovarian cancer, it enabled higher-dose administration and achieved non-inferiority for both PFS and overall survival (OS), with a slightly lower incidence of peripheral neuropathy compared with conventional CrEL-PTX, while requiring no routine corticosteroid premedication [127]. These results suggest that CrEL elimination and excipient optimization can, to a meaningful degree, improve the clinical dosing convenience and safety of PTX.

The limitations of these formulations, however, are also relatively clear. Although Lipusu avoids CrEL, premedication may still be required in clinical administration, and its dosing and neurotoxicity profile have not fully transcended the constraints of conventional PTX formulations [128]. Polymeric micelle formulations such as Genexol-PM can increase the administered dose of PTX, but the efficacy gain is inconsistent, and in vivo micelle structural stability remains a key factor affecting their genuine delivery advantage. Some studies suggest that polymeric micelles may undergo varying degrees of dissociation upon plasma dilution and during in vivo circulation, rendering their clinical function closer to that of a CrEL-free solubilization system rather than a stable nanostructure-maintaining targeted delivery platform.

Taken together, the principal clinical contributions of Lipusu, Genexol-PM, and Paclical remain concentrated in improving solubilization, reducing solvent-related toxicity, and optimizing the administration workflow. The Paclical experience suggests that the pharmacological design of the excipient itself may further improve the therapeutic window; however, overall, these first-generation or early nanoformulations have not yet adequately demonstrated the capacity to achieve stable tumor-targeted delivery and substantial efficacy improvement through the nanostructure per se.

#### 5.1.3. Clinical Limitations of First-Generation Nanoformulations

In summary, the clinical value of first-generation PTX nanoformulations primarily resides in eliminating CrEL, improving dosing tolerability, and enhancing clinical operational convenience. Their efficacy gains, however, remain constrained by two core prerequisites: first, whether the nanoparticles can stably accumulate in tumor tissue via the enhanced permeability and retention (EPR) effect; and second, whether the carrier can maintain sufficient structural integrity during in vivo circulation. Existing evidence indicates considerable uncertainty regarding both of these prerequisites in real in vivo settings. An analysis encompassing 297 studies and 534 datasets showed that the median tumor delivery efficiency of intravenously injected nanoparticles in tumor-bearing mice is approximately 0.67% of the injected dose (%ID) [10]. Reviews of the EPR effect have further noted that vascular permeability, interstitial pressure, and matrix architecture in human tumors exhibit marked heterogeneity, making it difficult for passive accumulation to translate stably into therapeutic benefit across different patients and tumor types [9].

At the carrier level, the in vivo instability of physical encapsulation further amplifies the above efficiency bottleneck. Nab-paclitaxel may undergo rapid dissociation of its nanostructure into albumin-bound or free forms following infusion, causing it to behave in vivo more as a CrEL-free solubilization alternative and making it difficult to sustain the tumor-selective delivery advantage expected of a nanostructured carrier [129]. Based on assessments of plasma dilution stability and in vivo structural retention, some studies have indicated that polymeric micelle formulations such as Genexol-PM may undergo varying degrees of disassembly following intravenous injection, such that their clinical role leans more toward solubilization than stable nano-targeted delivery. In addition, the EPR effect demonstrates significant inter- and intra-individual heterogeneity in human tumors, which is particularly pronounced in desmoplastic tumors such as pancreatic cancer [11].

Carrier structural stability further constrains the delivery advantage of first-generation formulations. For physically encapsulated systems, including albumin-bound, liposomal, and polymeric micelle formulations, carrier dissociation, protein exchange, or premature drug release may occur during blood circulation, thereby undermining the tumor-selective delivery that the nanoparticle structure is intended to achieve. Physiologically based pharmacokinetic (PBPK) modeling analysis indicates that nanoparticle tumor accumulation may gradually decline over time, suggesting that prolonged circulation time does not necessarily equate to increased effective intratumoral exposure [122]. The clinical performance of first-generation nanoformulations is also influenced by differences in tumor type and treatment context. In pancreatic ductal adenocarcinoma (PDAC) and other desmoplastic tumors, abnormally deposited extracellular matrix (ECM) and elevated interstitial fluid pressure (IFP) restrict nanoparticle convection and deep penetration, making it difficult for delivery strategies that rely solely on the EPR effect to adequately overcome these physical barriers. In immunotherapy combination settings such as TNBC, the advantage of nab-paclitaxel derives in part from its compatibility with ICI regimens, conferred by the absence of CrEL and reduced need for corticosteroid premedication, rather than necessarily representing true precision tumor targeting. This also underscores that the clinical value of first-generation nanoformulations should be understood in the context of specific treatment scenarios, rather than being simplistically attributed to “nanonization” itself.

Furthermore, first-generation nanoformulations are generally incapable of actively intervening in intracellular resistance networks. PTX is a prototypical substrate of *P-gp*/*ABCB1*; even if nanoparticles increase drug exposure in tumor tissue or cells, the released free PTX may still be subject to efflux pumps, microtubule alterations, anti-apoptotic signaling, and TME adaptation, among other resistance mechanisms. Merely increasing drug delivery quantity through physical encapsulation is therefore typically insufficient to stably reverse acquired resistance. The subsequent development of delivery systems needs to further transition from “solubilization and toxicity reduction” toward “mechanism-driven delivery”, that is, establishing a more verifiable link between drug exposure and clinical benefit through improved in vivo stability, enhanced tissue penetration, controlled release kinetics, and the integration of resistance-intervention modules.

### 5.2. Clinical Research Progress of Next-Generation PTX Delivery Systems

#### 5.2.1. Clinical Progress of Next-Generation Polymeric Micelle Formulations

NK105 is among the more representative clinical cases of next-generation PTX micelle formulations. This formulation optimizes the polymer structure to reduce premature PTX release in the circulation. In a phase III randomized controlled trial, 436 patients with metastatic or recurrent breast cancer were randomized, of whom 422 were included in the efficacy analysis. NK105 did not meet the primary endpoint of PFS non-inferiority compared with conventional PTX; however, the incidence of grade ≥3 peripheral sensory neuropathy was markedly lower in the NK105 arm [130]. Subsequent studies observed a persistently low incidence of severe neurotoxicity even at higher dose levels [131]. These findings indicate that micelle stability optimization can, to a degree, improve the safety and therapeutic window of PTX, but that passive enrichment or prolonged circulation alone may still be insufficient to overcome the efficacy uncertainty posed by EPR effect heterogeneity in human tumors.

Polymeric micelle PTX formulations developed in China have also shown a degree of clinical advancement potential. In a phase I study of ZSYY001, the dose was escalated stepwise from 175 mg/m^2^ to 390 mg/m^2^ without reaching the maximum tolerated dose; no acute hypersensitivity reactions were observed, and objective responses occurred in a subset of evaluable patients [132]. In addition, a retrospective study by Wang et al. comparing nanoscale polymeric micelle PTX (NPMP) combined with fluorouracil versus conventional PTX combination regimens in advanced gastric cancer showed a higher objective response rate in the NPMP group, along with lower incidences of certain hematologic and gastrointestinal adverse events [133]. This study was, however, retrospective in design with a limited sample size, and may have been influenced by factors including treatment line, baseline characteristics, and regimen selection; its efficacy advantage therefore still requires validation through prospective randomized studies.

In the immunotherapy combination direction, polymeric micelle PTX has also begun to enter early-phase clinical exploration. A conference abstract reported phase II results for pm-Pac combined with sintilimab as first-line treatment for non-squamous NSCLC, showing high objective response rates and 12-month PFS rates [134]. This finding suggests that CrEL-free micelle formulations with reduced corticosteroid premedication requirements may be better suited as the chemotherapy backbone in immunotherapy combination regimens. However, given that the evidence derives from early-phase studies and a conference abstract, its clinical significance requires further confirmation through fully published data and randomized controlled trials.

Overall, the clinical progress of next-generation PTX micelle formulations indicates that their more clearly established advantages primarily reside in safety improvement, dose-escalation potential, and combination therapy compatibility. By contrast, definitive efficacy gains derived solely from passive tumor enrichment still lack robust evidence. This landscape also provides a reference for the translational evaluation of subsequent co-delivery and active targeting systems: novel delivery systems need to demonstrate not only that they “load PTX better,” but also that they can stably alter drug exposure, toxicity profiles, or therapeutic response in humans.

#### 5.2.2. Co-Delivery and Active Targeting: Preclinical Activity and the Clinical Evidence Gap

In contrast to polymeric micelle formulations, which have progressively entered clinical investigation, PTX co-delivery systems and active-targeting ligand-modified nanoformulations remain sparsely represented in published randomized clinical trials. The available clinical evidence is predominantly concentrated around mature platforms such as nab-paclitaxel, whereas PTX nanosystems with genuine multi-drug synchronous delivery, programmed release, or ligand-mediated active targeting characteristics largely remain at the preclinical research stage.

Based on the composition of published clinical evidence, randomized studies of PTX nanoformulations remain heavily concentrated on a small number of mature carriers. A meta-analysis by Deng et al., encompassing 15 randomized trials and 4925 patients, did not include typical co-delivery or ligand-targeted systems [135]. Another systematic review published in 2024, covering 31 randomized clinical trials of nanomedicines, the majority of which involved mature platforms such as nab-paclitaxel, likewise found no evidence that active-targeting ligand-modified carriers have yet generated a substantial body of clinical data [136]. This indicates that the PTX nanoformulations genuinely validated in the clinic to date remain predominantly characterized by “CrEL elimination” and “improved dosing characteristics,” rather than by complex targeting or multi-drug co-delivery systems.

In contrast, co-delivery strategies are highly active in preclinical research. For example, PTX co-loaded with tetrandrine in methoxy poly(ethylene glycol)–poly(lactic-co-glycolic acid) (mPEG–PLGA) nanoparticles can achieve sequential release and resistance reversal through a redox-responsive mechanism in a HeLa multidrug resistance (MDR) model [137]; another study co-loaded PTX with the colony-stimulating factor 1 receptor (CSF1R) inhibitor PLX3397 in polymeric micelles, demonstrating synergistic effects of chemotherapy and immune microenvironment modulation in a TNBC model [138]. These results indicate that co-delivery strategies hold strong mechanistic appeal; their clinical translation, however, remains constrained by uncertainties in in vivo efficacy, drug ratio control, release kinetics verification, and the complexity of manufacturing and quality control.

The clinical translation of active-targeting nanoformulations faces similar challenges. BIND-014 is a prostate-specific membrane antigen (PSMA)-targeted docetaxel polymeric nanoparticle. Although the loaded drug is not PTX, docetaxel belongs to the same taxane class, and the carrier strategy is likewise based on ligand-mediated active targeting; BIND-014 can therefore serve as an analogous case illustrating the translational difficulties of taxane-based active-targeting nanomedicines. This formulation demonstrated a tolerable safety profile and some antitumor activity in phase II studies [139], but subsequent development did not yield a clear efficacy advantage. This case highlights that ligand–receptor recognition strategies validated in vitro or in animal models may, upon entering the human body, be affected by factors including protein corona shielding, target heterogeneity, distance from tumor vasculature, and tissue penetration limitations, and may consequently fail to translate stably into clinical benefit.

Thus, the clinical evidence gap for co-delivery and active targeting systems does not imply that these strategies lack scientific value; rather, it indicates that their translational threshold is higher than that of first-generation solubilization-type or micelle-type formulations. Compared with single-drug micelles, co-delivery systems need to simultaneously demonstrate that multi-drug ratios can be maintained in vivo, that the release sequence is verifiable, that combined toxicity is manageable, and that the manufacturing process meets Good Manufacturing Practice (GMP) scale-up requirements. Active targeting systems, for their part, need to demonstrate that target expression, ligand exposure, and tissue penetration remain reproducible in humans. Only when these critical issues are resolved can preclinical “synergy-oriented” designs realistically translate into randomized clinical evidence.

In summary, the evidence in Section 5.2 is stratified rather than uniformly positive. Polymeric micelle formulations have progressed furthest clinically, but the type and strength of evidence differ by platform. NK105 showed a lower incidence of severe peripheral sensory neuropathy but did not meet the phase III PFS non-inferiority endpoint [130,131]. ZSYY001 is supported by early-phase clinical evidence [132], NPMP by retrospective data [133], and pm-Pac-based combinations by a phase II conference abstract [134]. These studies report platform-specific advantages, including CrEL-free administration, dose-escalation potential, or compatibility with combination regimens, but they do not establish class-wide clinical superiority. Co-delivery and active-targeting systems remain dominated by preclinical evidence. Together, these candidates illustrate both the progress achieved and the evidentiary threshold that continues to separate technical formulation advantages from reproducible clinical benefit. The regulatory status, clinical indications, therapeutic advantages, and remaining limitations of representative approved and investigational PTX nanoformulations are compared in Table 2.

### 5.3. Clinical Translation Challenges for Novel PTX Delivery Systems

#### 5.3.1. In Vivo Delivery Barriers and Limitations of Preclinical Model Extrapolation

Although novel PTX nanodelivery systems exhibit considerable design flexibility in preclinical studies, they must first confront systemic barriers upon entering the body, including mononuclear phagocyte system (MPS) clearance, plasma protein adsorption, and insufficient tumor vascular extravasation efficiency. Upon entering the blood circulation, plasma proteins can rapidly adsorb onto the nanoparticle surface to form a protein corona, altering the original surface properties and potentially masking targeting ligands or promoting opsonin-mediated phagocytic clearance. Consequently, the stability, targeting recognition capacity, and release behavior exhibited by a carrier in vitro may not be fully preserved in the complex in vivo environment.

Even if nanoparticles succeed in reaching the tumor vascular bed, the ECM, aberrant vascular architecture, and elevated IFP within solid tumors still restrict their diffusion into deep tumor regions. This problem is particularly pronounced in matrix-rich tumors such as PDAC. Compared with the subcutaneous xenograft models commonly used in preclinical research, human solid tumors typically possess more complex vascular distributions, higher matrix density, and greater spatial heterogeneity; the tumor accumulation and efficacy advantages observed in animal models therefore do not necessarily extrapolate linearly to clinical patients.

**Figure 8:**
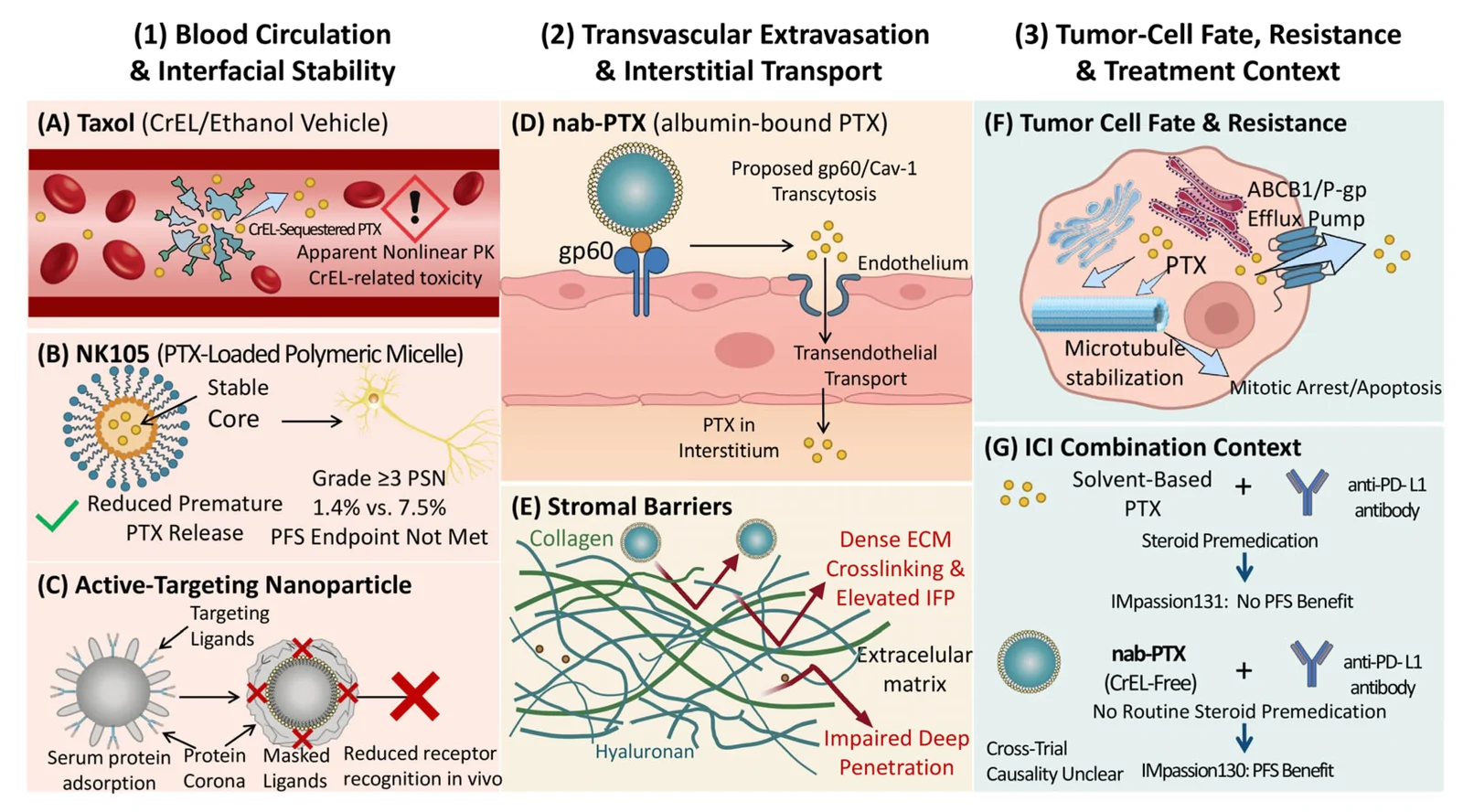
Formulation-dependent in vivo fate and translational bottlenecks of paclitaxel (PTX) nanomedicines. The schematic organizes formulation behavior and translational barriers across three levels: (**1**) blood circulation and interfacial stability; (**2**) transvascular extravasation and interstitial transport; and (**3**) tumor-cell fate, resistance, and treatment context. (**A**) Conventional Taxol uses a Cremophor EL (CrEL)/ethanol vehicle. CrEL-associated sequestration of PTX contributes to apparent nonlinear pharmacokinetics (PK) and formulation-related toxicity. (**B**) NK105 is a PTX-loaded polymeric micelle designed to improve micellar-core stability and reduce premature PTX release. In a phase III trial, the incidence of grade ≥ 3 peripheral sensory neuropathy (PSN) was 1.4% with NK105 versus 7.5% with conventional PTX, although the progression-free survival (PFS) non-inferiority endpoint was not met [130]. (**C**) Serum–protein adsorption and protein–corona formation can mask targeting ligands and reduce receptor recognition in vivo. (**D**) Nanoparticle albumin-bound paclitaxel (nab-PTX) is proposed to undergo endothelial transport involving the albumin receptor gp60 (albondin) and caveolin-1 (Cav-1)-associated transcytosis. (**E**) A collagen- and hyaluronan-rich extracellular matrix (ECM), ECM crosslinking, and elevated interstitial fluid pressure (IFP) restrict interstitial transport and deep tumor penetration. (**F**) Intracellular PTX stabilizes microtubules and promotes mitotic arrest and apoptosis, whereas ATP-binding cassette subfamily B member 1 (*ABCB1*)/*P-glycoprotein* (*P-gp*)-mediated efflux reduces intracellular PTX exposure and contributes to multidrug resistance. (**G**) The clinical context of immune checkpoint inhibitor (ICI) combinations is illustrated by the IMpassion130 and IMpassion131 trials. IMpassion130 reported a PFS benefit with nab-PTX plus atezolizumab, an antibody against programmed death-ligand 1 (anti-PD-L1), whereas IMpassion131 did not demonstrate a PFS benefit with solvent-based PTX plus atezolizumab [124,143]. Solvent-based PTX requires steroid premedication, whereas nab-PTX is CrEL-free and generally does not require routine steroid premedication. Nevertheless, the differences between these trials are likely multifactorial, and cross-trial comparison does not establish causality. The overall composition was created by the authors using Microsoft PowerPoint 2024 and is not reproduced or adapted from any previously published figure. Selected graphical elements were sourced from SciDraw and BioIcons and were used or adapted under their respective Creative Commons or public-domain licenses, while all remaining graphical elements were drawn by the authors.

In anatomically privileged sites such as central nervous system tumors, the blood–brain barrier (BBB) further restricts the entry of PTX and its nanoformulations into the brain parenchyma. Techniques such as low-intensity pulsed ultrasound combined with microbubbles can transiently increase BBB permeability, but barrier opening does not automatically equate to improved clinical efficacy. Recent studies in glioblastoma suggest that biomarker changes following BBB opening may correlate with patient response to nab-paclitaxel, indicating that the establishment of a delivery conduit is only the first step; the individual patient’s microenvironment and drug response status still substantially influence the ultimate benefit [142]. Future delivery strategy evaluation should therefore not only examine whether nanoparticles can enter the tumor, but should further investigate their spatial distribution within the tumor, effective drug exposure, and inter-patient variability. These formulation-dependent processes and translational constraints across circulation, tumor entry, interstitial transport, intracellular fate, and treatment context are summarized in Figure 8.

#### 5.3.2. Pharmaceutics and Formulation Bottlenecks: Stability, Scale-Up Manufacturing, and Quality Control

Beyond in vivo barriers, the pharmaceutical manufacturability of complex nanoformulations is also a critical factor limiting clinical translation. Multifunctional PTX nanoplatforms typically incorporate polymers, lipids, targeting ligands, responsive linkers, or multiple therapeutic components. Although they can achieve favorable particle size, encapsulation efficiency, and release profiles at the laboratory scale, whether batch-to-batch consistency can be maintained upon scale-up remains a central concern. For nanoformulations, particle size distribution, composition, purity, surface properties, drug loading, and release kinetics all constitute critical quality attributes (CQAs); any minor variation may affect in vivo distribution, drug release, and safety.

Furthermore, stability testing in conventional buffers does not adequately represent the systemic circulatory environment. Upon entry into the blood, serum protein adsorption, lipoprotein exchange, and dilution effects can all disrupt micelles or other self-assembled structures, leading to premature drug release or carrier disintegration. Relevant studies have shown that PTX release behavior in complex biological media can differ markedly from that in simplified in vitro release systems, underscoring the need to evaluate the in vivo stability of nanoformulations using detection methods that more closely approximate the physiological environment. Relying solely on aqueous-phase particle size stability or buffer-based release curves is therefore insufficient to reliably predict real behavior following clinical administration.

To improve translational feasibility, future formulation design needs to strike a balance between functional complexity and manufacturing controllability. On the one hand, manufacturing technologies such as continuous-flow micromixing, in-line purification, and standardized lyophilization processes can help improve the reproducibility of particle size, polydispersity index (PDI), drug loading, and release behavior. On the other hand, carrier-free or low-carrier designs can reduce the proportion of inert excipients, decrease material heterogeneity, and enhance the operational feasibility of quality control [144,145,146]. Overcoming chemistry, manufacturing, and controls (CMC) bottlenecks therefore does not mean continuing to stack additional functional modules; rather, it requires that the delivery system achieves verifiable consistency across in vitro performance, manufacturing scale-up, quality control, and in vivo stability.

#### 5.3.3. Clinical Resistance Networks and Patient Heterogeneity

The clinical translation of PTX nanodelivery systems is also constrained by the complexity of tumor resistance networks. *P-gp*/*ABCB1*-mediated drug efflux is one important mechanism of PTX resistance, but clinical resistance is not determined by efflux pumps alone. Factors including tubulin alterations, epithelial–mesenchymal transition (EMT), tumor stem cell enrichment, hypoxia, immunosuppressive microenvironment, and metabolic reprogramming can all influence the cell-killing efficacy of PTX. These mechanisms are not uniformly operative across different tumor types, different patients, or even different regions of the same tumor, resulting in substantial inter-patient variability in response to a given nanoformulation.

This also suggests that merely increasing the total quantity of drug delivered to tumor tissue may be insufficient to overcome clinical resistance. Certain studies, although not directly focused on PTX, provide important conceptual insights: altering the route by which a drug enters the cell may circumvent some traditional resistance mechanisms. For instance, a cisplatin nanoformulation that enters via the endocytic pathway bypassed a specific drug uptake channel deficiency and restored cytotoxicity against drug-resistant cells [147]. For PTX nanodelivery, this concept suggests that future design should not focus solely on “how much drug reaches the tumor,” but also on “in what form the drug enters the cell, where it is released, and whether it can bypass intracellular resistance nodes”.

Patient heterogeneity likewise affects the clinical benefit derived from nanoformulations. For example, in a study of gastric cancer with peritoneal dissemination, a nab-paclitaxel combination regimen did not show a significant OS advantage in the overall population, but patients with high stromal Caveolin-1 (Cav-1) expression may have exhibited a more pronounced trend toward benefit [148]. In the neoadjuvant treatment of TNBC, immune-related gene signatures have been shown to predict pathological complete response (pCR) to nab-paclitaxel-based regimens in a subset of patients [149]. These findings indicate that the efficacy of nanoformulations may depend on specific vascular transport, stromal status, or immune microenvironment characteristics. Without patient stratification, potentially benefiting subpopulations may be diluted by an overall negative result.

The biomarker findings above should be interpreted according to their level of evidence. Stromal Cav-1 and immune-related gene signatures have shown associations with differential response in specific clinical cohorts, but neither has been established as a clinically validated patient-selection biomarker for PTX nanoformulations [148,149]. These findings require independent validation before they can be used prospectively for treatment selection. Circulating tumor DNA (ctDNA) is discussed here only as a potential future monitoring or stratification tool and has not been established for patient selection in PTX nanomedicine. Accordingly, these markers should currently be described as candidate or exploratory biomarkers rather than established clinical tools.

The clinical translation of novel PTX nanodelivery systems should therefore not take increased tumor accumulation or reduced systemic toxicity as the sole evaluation endpoints, but should simultaneously attend to patient microenvironment characteristics, resistance mechanism composition, and detectable biomarkers. Only by establishing clearer correspondences among delivery system design, formulation quality control, and patient stratification strategies can subsequent clinical studies more accurately identify the populations that truly benefit. Computational approaches, including AI, may further support formulation optimization, translational evaluation, and patient stratification.

### 5.4. Applications of Computational Methods and AI in PTX Nanomedicine R&D

The translational bottlenecks described above indicate that the clinical development of novel PTX nanodelivery systems is not solely a matter of materials innovation, but also involves multidimensional variables including formulation parameters, in vivo stability, manufacturing scale-up, quality control, and patient heterogeneity. The traditional R&D model, reliant on empirical screening and iterative trial-and-error, has limited efficiency in high-dimensional formulation spaces and complex clinical scenarios. Computational simulation, machine learning (ML), and AI are therefore emerging as important digital tools for shortening the development cycle, reducing the cost of unguided trial-and-error at the CMC stage, and supporting precision translation.

The applications discussed below should therefore be interpreted as proof-of-concept or early-stage decision-support approaches rather than clinically validated tools. Their apparent performance depends strongly on dataset size and representativeness, descriptor quality, endpoint definition, and validation design.

#### 5.4.1. Molecular Simulation in PTX Mechanism and Carrier Design

Computational simulation first provides a molecular-level explanatory foundation for the mechanism of action of PTX and for carrier construction. All-atom molecular dynamics (MD) simulations have shown that PTX binding to β-tubulin reduces the flexibility of the M-loop region, thereby stabilizing microtubule structure [150]. Further free energy decomposition studies have revealed that the hydrogen-bond network formed by key residues such as Thr276 and Arg278, together with van der Waals interactions, plays an important role in maintaining the high affinity of PTX; point mutations in β-tubulin can weaken binding stability and are associated with the resistance phenotype [151]. These investigations not only contribute to understanding the structural basis of PTX resistance, but also provide a reference for the structural optimization of novel taxane derivatives.

At the delivery system level, MD simulations can be used to analyze the spatial distribution and stabilization mechanisms of PTX within nanocarriers. For instance, coarse-grained or all-atom simulations have shown that PTX tends to embed within the hydrophobic core region of lipid bilayers, which helps to enhance the structural stability of liposomes or biomimetic nanovesicles and reduce drug leakage during circulation [152,153]. Similar approaches can also be applied to polymeric micelles, peptide assemblies, and certain inorganic carrier systems to evaluate drug–carrier interactions, self-assembly stability, and potential release behavior. Overall, MD simulations provide molecular-level structural input for PTX nanoformulations, but the reliability of their predictions still depends on force-field accuracy, model boundary conditions, and subsequent experimental validation.

#### 5.4.2. AI in Formulation Optimization and Material Screening

In contrast to the mechanism-elucidation focus of MD, AI and ML are better suited to handling multivariate formulation optimization and large-scale candidate system screening. For active-targeting delivery, Jang et al. constructed a CXCR4-targeted core–shell nanocarrier for co-delivery of PTX and a sensitizer [15]. This study integrated AlphaFold structural prediction, CABS-dock flexible docking, the NeuroSNAP-AI scoring platform, and MD-Syn drug synergy prediction to screen for peptide ligands with high CXCR4 affinity and to identify berberine (BBR) as having potential synergistic activity with PTX. This case exemplifies a complete computational workflow spanning receptor structure prediction, ligand screening, and combination therapy optimization, illustrating the application value of AI in the early-stage design of targeted nanoformulations.

In formulation optimization, ML can establish mapping relationships between formulation parameters and CQAs. Models such as artificial neural networks (ANNs) and random forests can take lipid ratios, polymer compositions, and process conditions as input variables to predict encapsulation efficiency, drug loading capacity, particle size, and zeta potential [154]. Furthermore, regression models or inverse optimization algorithms can back-calculate preferred formulation windows based on preset targets for particle size, charge, or drug loading [155]. Such methods are directly relevant to the CMC challenges discussed in Section 5.3, because their potential value lies not merely in improving the success rate of individual experiments, but in helping developers identify, at an early stage, formulation regions that are more manufacturable, scalable, and quality-controllable.

AI can also be applied to the high-throughput screening of novel materials and co-assembly systems. For example, ML combined with grand canonical Monte Carlo (GCMC) simulations has been used to screen over 86,000 metal–organic framework (MOF) structures for candidates suitable for PTX delivery, identifying PCN-222 as a potential carrier [156]. In addition, logistic regression and support vector machine (SVM) models based on small-molecule physicochemical descriptors can predict the potential of small molecules to co-assemble with PTX into nanoparticles, achieving an accuracy of 91.89% [157]. These results indicate that AI is driving a transition in PTX nanoformulation R&D from empirical to data-driven approaches, with the potential to reduce unproductive iterations in early-stage screening and formulation development.

#### 5.4.3. Data-Driven Models in Precision Therapy and Clinical Decision-Making

Beyond formulation R&D, AI is beginning to enter the domains of individualized PTX therapy and clinical benefit prediction. The in vivo clearance rate of PTX exhibits inter-individual variability and may be influenced by genetic background. Chen et al. used the progressive deep learning algorithm GEP-CSI to analyze interaction networks among genes including *FLT1*, *EGF*, and *MUC16*, predicting individualized PTX clearance in NSCLC patients and providing a framework for dose adjustment and individualized pharmacokinetic modeling [158]. Although such models still require larger-scale clinical validation, their significance lies in advancing conventional population pharmacokinetic parameters toward patient-level risk prediction.

In efficacy prediction and patient stratification, ML models have likewise shown potential. A multivariate model constructed using random forest algorithms can predict the likelihood of disease-free survival benefit from PTX adjuvant chemotherapy in gastric cancer patients [159]. Of greater translational relevance, the EXEMPLAR model integrates liquid biopsy-derived exosomal miRNA data to predict objective response and OS in patients with advanced gastric cancer receiving PTX plus ramucirumab, achieving an area under the curve (AUC) of 0.87 [160]. This indicates that the combination of circulating biomarkers and ML may provide non-invasive tools for predicting the efficacy of PTX-based regimens.

In the context of complex drug resistance, deep learning models can also integrate multi-dimensional omics and cellular state information. For instance, the Beyondcell-based single-cell resistance signature identification and KANSurv survival prediction framework can be used to analyze PTX resistance-associated subtypes in ovarian cancer and to improve prognostic prediction under complex resistance conditions [161]. These models directly address the patient heterogeneity bottleneck raised in Section 5.3: if novel PTX nanoformulations are to demonstrate stable benefit in clinical trials, they cannot rely solely on overall population evaluation, but must incorporate biomarker, resistance signature, and microenvironment status-based patient selection. Data-driven stratification strategies have the potential to optimize enrollment design and reduce the risk of potentially benefiting subpopulations being diluted by an overall negative result.

#### 5.4.4. Current Limitations and Future Directions

Despite their potential, AI and machine-learning (ML) approaches in PTX nanomedicine remain constrained by data scope and transportability. Available datasets differ in formulation descriptors, material-characterization methods, biological models, dose schedules, and clinical endpoints, which limits comparability across studies [154,155]. Validation rigor is also uneven. Some studies have used external validation cohorts [159,161], and a recent liquid-biopsy study was conducted in a prospective clinical cohort [160]; however, these models remain tied to particular material families, disease settings, populations, or assay platforms. Missing variables and restricted sample diversity may therefore reduce reproducibility and encourage study-specific rather than broadly generalizable relationships. Reported predictive performance should not be equated with clinical transferability until models are independently reproduced across laboratories and populations, prospectively evaluated where appropriate, and linked to standardized formulation and clinically relevant endpoints.

In the future, if AI-driven high-throughput virtual screening can be integrated with automated synthesis and experimental validation platforms, a “predict–synthesize–validate” closed-loop R&D model may emerge. Digital twin technology may also, through the construction of patient-individualized TME models, provide a simulation platform for PTX delivery strategy selection and dose optimization. Concurrently, the fusion of multimodal data, genomics, radiomics, liquid biopsy, and clinical outcomes will help to establish PTX treatment response prediction systems and support more precise patient stratification. Overall, the value of computational methods lies not in replacing experimental and clinical research, but in improving the efficiency of candidate system screening, explaining complex structure–performance relationships, and facilitating the progression of PTX nanodelivery systems from preclinical design toward genuine patient benefit.

## 6. Conclusions and Future Perspectives

PTX, as a classical microtubule-stabilizing agent, remains an important chemotherapeutic drug in the treatment of multiple solid tumor types. Its clinical application, however, has long been constrained by poor aqueous solubility, solvent-related toxicity, systemic adverse effects, and multidrug resistance (MDR). Over the past several decades, PTX delivery systems have progressed from conventional solvent-based formulations through albumin-bound formulations, liposomes, and polymeric micelles to multifunctional nanoplatforms. Viewed in aggregate, the principal clinical contribution of first-generation nanoformulations has been to improve solubilization, reduce Cremophor EL (CrEL)-related toxicity, and enhance dosing convenience. These improvements have not, however, adequately resolved the problems of insufficient deep tumor delivery, intracellular drug efflux, microenvironmental barriers, and inter-patient variability in therapeutic response. The developmental objective of PTX delivery systems has therefore progressively shifted from merely “enhancing solubility and reducing toxicity” toward “mechanism-driven precision delivery”.

In response to the complex tumor microenvironment (TME), strategies including stimuli-responsive delivery, dynamic size/charge transition, biomimetic membrane coating, matrix remodeling, and carrier-free prodrug self-assembly have furnished a diverse array of approaches for improving PTX delivery efficiency. These systems exploit features such as local tumor pH, glutathione (GSH), enzymatic activity, hypoxia, matrix abnormalities, and immune evasion to achieve staged regulation of drug release, tissue penetration, and cellular uptake. Multi-drug co-delivery strategies further integrate PTX with sensitizers, nucleic acid therapeutics, immunomodulatory agents, or physical therapeutic units within a single system, seeking to intervene in resistance networks through ratio control, subcellular spatial allocation, and sequential release. Overall, novel delivery systems have evolved beyond serving merely as drug “packaging materials” and are progressively becoming engineered therapeutic platforms capable of modulating drug exposure, the TME, and cell fate.

Despite rapid progress in preclinical research, the clinical translation of novel PTX delivery systems continues to face significant challenges. The pronounced heterogeneity of the enhanced permeability and retention (EPR) effect in human tumors, mononuclear phagocyte system (MPS) clearance, protein corona formation, dense extracellular matrix (ECM), and elevated interstitial fluid pressure (IFP) all compromise the effective delivery of nanoparticles. Available quantitative analyses indicate that the proportion of intravenously injected nanoparticles reaching solid tumors is, overall, limited, with a median value of approximately 0.67% of the injected dose [11]; at the same time, the contribution of approved nanomedicines to clinical outcomes still, to a substantial degree, manifests as improved tolerability or dosing convenience rather than as a robust survival benefit [112]. In addition, the complex material composition and multi-step fabrication procedures of multifunctional carriers increase the difficulty of scalable manufacturing, stability evaluation, and quality control. Clinical drug resistance, moreover, is not determined solely by *P-glycoprotein* (*P-gp*)-mediated efflux, but rather constitutes a dynamic network jointly shaped by drug efflux, microtubule alterations, hypoxia, immunosuppression, tumor stem cell enrichment, and metabolic reprogramming. The association between Caveolin-1 (Cav-1) expression status and differential nab-paclitaxel efficacy observed in the P-SELECT trial, together with the predictive value of immune gene signatures for pathological complete response (pCR) rates in the WSG-ADAPT-TN trial, both indicate that the biological background of the patient may significantly influence the therapeutic benefit derived from PTX formulations. Consequently, in the absence of patient stratification and biomarker guidance, even delivery systems that perform impressively in preclinical models may have their signal diluted within a heterogeneous patient population, making it difficult to demonstrate stable benefit.

As delivery system complexity continues to increase, computational methods and artificial intelligence (AI) are emerging as important auxiliary tools connecting formulation design, translational evaluation, and patient stratification. Molecular dynamics (MD) simulations can help to elucidate PTX–carrier interactions, self-assembly stability, and potential release behavior; machine learning (ML) and AI methods can be applied to targeting ligand screening, formulation parameter prediction, drug combination optimization, pharmacokinetic (PK) modeling, and efficacy prediction. The value of these methods lies not in replacing experimental validation or clinical investigation, but in improving the efficiency of candidate system screening, reducing uninformed trial-and-error, and explaining structure–performance relationships in complex delivery systems. In the future, computation-assisted R&D will need to be more closely integrated with in vivo PK, tumor distribution, quality control, and clinical response data before it can transition from a descriptive analytical tool into a design platform that genuinely supports the translational development of PTX nanomedicines.

The future development of PTX delivery systems should place greater emphasis on three directions. First, the starting point for delivery system design should shift from “how to load PTX” toward “what exploitable biological features characterize the tumor in the target patient population,” with priority given to validation in patient subgroups possessing relatively well-defined characteristics, such as matrix density, receptor expression, degree of immune infiltration, or resistance mechanisms. Second, candidate-biomarker assessment should be incorporated into early-phase clinical trial design, for instance Cav-1 status, immune gene signatures, circulating tumor DNA (ctDNA), or tumor stromal characteristics, rather than being relegated to post hoc exploratory analyses; the question of “for whom are we delivering?” should be addressed before deciding “how to deliver.” Third, formulation development should move from the mere accumulation of additional functionalities toward translational designs that are manufacturable, quality-controllable, and verifiable, with priority given to carrier scaffolds that already have a clinical application foundation, using a limited but well-chosen set of response nodes to achieve the necessary spatiotemporal control, and incorporating stability in physiologically complex media, protein corona effects, and actual intratumoral drug exposure as essential evaluation metrics.

PTX nanodelivery research has entered a new phase characterized by intelligent, multifunctional, and translation-oriented design. The research of the past several decades has demonstrated that nanotechnology can, to a meaningful degree, improve the PK, safety, and administration of PTX; however, the real clinical benefit of complex delivery systems has yet to fully match the rapid growth in their design complexity. Although increasingly sophisticated delivery systems continue to emerge, convincing clinical evidence demonstrating that rational carrier design can improve drug exposure, modulate resistance, and ultimately translate into meaningful clinical benefit remains limited. The key to future breakthroughs lies not in the continued accretion of additional functional modules, but in adopting a clearly defined clinical question as the guiding principle and striking a balance among delivery efficiency, safety, manufacturing feasibility, and patient selection. Only when novel delivery systems can simultaneously satisfy criteria of mechanistic plausibility, formulation controllability, patient stratification, and clinical verifiability will this classical drug, PTX, be positioned to realize new therapeutic value in the era of precision nanomedicine.

## Figures and Tables

**Table 1:** Representative experimental paclitaxel (PTX) nanodelivery platforms and their principal translational attributes.

Delivery Strategy/Carrier	Trigger/Transition Mechanism	Particle Size/Surface-Charge Properties	Drug Loading	
Hypoxia-activated PEGylated PTX prodrug nanoparticles [43]	Hypoxia-induced cleavage of an azo linker releases PTX	110.3 ± 1.4 nm; negatively charged	PTX content, 42.6 ± 1.3 wt%	
Excipient-free disulfide-linked PTX–maleimide prodrug nanoparticles [48]	Albumin binding induces size reduction; intracellular reduction releases PTX	185.5 ± 10.3 nm initially; approximately 10 nm after exposure to albumin	Pure prodrug nanoparticle; carrier-based loading is not applicable	
pH-responsive charge-switchable PTX/DSF polymeric micelles [50]	Protonation of the polymer corona under mildly acidic conditions reverses surface charge	138 ± 8.5 nm at pH 7.4 after 24 h; negative at pH 7.4 and positive at pH 6.5	PTX, 10.73 ± 0.50%; DSF, 1.97 ± 0.80%	
ROS-sensitive PTX-prodrug/DOX liposomes [82]	ROS-sensitive PTX-prodrug activation with remotely loaded DOX at a 5:1 ratio	129.20 ± 2.80 nm; zeta potential, −24.80 ± 0.78 mV	PTX prodrug, 14.94 ± 0.01%; DOX, 3.00 ± 0.15%	
HA-coated PTX/DSF cocrystal nanorods with anchored Cyt C [80]	HA-mediated uptake with intracellular delivery of PTX, DSF, and Cyt C	186.12 ± 1.04 nm; zeta potential, −10.00 ± 0.68 mV	PTX, 43.22 ± 0.38%; DSF, 8.53 ± 0.23%; Cyt C, 2.75 ± 0.13%; total, 54.5% (*w*/*w*)	
Disulfide-linked PTX–CA4 prodrug nanoparticles [79]	Intracellular GSH cleaves disulfide bonds and promotes release of PTX and CA4	Approximately 124 nm (30% DLC formulation)	DLC, 30% in subsequent studies; maximum, 99% at a 1:0.01 drug/carrier feed ratio	
**Delivery Strategy/Carrier**	**Experimental Model(s)**	**Major Advantage**	**Principal Limitation**	**Development/Clinical Status**
Hypoxia-activated PEGylated PTX prodrug nanoparticles [43]	HeLa, A549, and 4T1 cells; subcutaneous 4T1 tumor-bearing mice	High PTX content with hypoxia-activated release	Preclinical cell and mouse evaluation only	Preclinical
Excipient-free disulfide-linked PTX–maleimide prodrug nanoparticles [48]	4T1, KB, and NIH/3T3 cells; 4T1 xenograft-bearing BALB/c mice	Excipient-free assembly with albumin-induced size reduction	Preclinical cell and mouse evaluation only	Preclinical
pH-responsive charge-switchable PTX/DSF polymeric micelles [50]	MCF-7 and MCF-7/ADR cells	Combines charge reversal with PTX/DSF co-delivery in a resistant cell model	Only cell-based in vitro evidence was reported	Preclinical (in vitro)
ROS-sensitive PTX-prodrug/DOX liposomes [82]	4T1 and HeLa cells; 4T1 tumor-bearing BALB/c mice	High loading efficiency and retention of a synergistic ratio in the 4T1 model	No clinical assessment of intratumoral ratio stability	Preclinical
HA-coated PTX/DSF cocrystal nanorods with anchored Cyt C [80]	A549 and A549/Taxol cells; A549/Taxol-resistant tumor-bearing mice	High total loading with simultaneous delivery of three therapeutic components	Preclinical evaluation only	Preclinical
Disulfide-linked PTX–CA4 prodrug nanoparticles [79]	CT26 and 4T1 cells; CT26 tumor-bearing mice	Synchronous PTX/CA4 delivery with tunable, exceptionally high DLC	The 99% maximum-DLC formulation was not used for biological studies	Preclinical

Notes: Abbreviations: CA4, combretastatin A4; Cyt C, cytochrome c; DLC, drug loading capacity; DOX, doxorubicin; DSF, disulfiram; GSH, glutathione; HA, hyaluronic acid; PEG, polyethylene glycol; PTX, paclitaxel; ROS, reactive oxygen species; wt%, weight percentage. Values are mean ± SD where reported. Loading metrics follow each primary source and are not necessarily directly comparable. Cross-row efficacy comparisons require caution because models, doses, schedules, and outcomes differ.

**Table 2:** Comparative clinical profiles of approved and clinically investigated paclitaxel (PTX) nanoformulations.

Formulation	Carrier and Approximate Size	Current Regulatory/Development Status	Approved or Evaluated Indication	
nab-Paclitaxel (Abraxane)	Albumin-bound PTX particles; approximately 130 nm	Approved in the United States since 2005 and in multiple other regions	US label: metastatic breast cancer; first-line locally advanced/metastatic NSCLC with carboplatin; first-line metastatic pancreatic adenocarcinoma with gemcitabine	
Paclitaxel liposome (Lipusu)	Lecithin/cholesterol liposome; approximately 400 nm	Marketed in China; regulatory indications are jurisdiction-specific	Breast, ovarian, and non-small cell lung cancers are reported clinical uses in China; gastric cancer and locally advanced/metastatic lung squamous cell carcinoma have been evaluated in clinical studies	
Genexol-PM	mPEG–PDLLA polymeric micelles; 20–50 nm	Marketed in South Korea and several Asian countries; phase III trial completed	Regional indications include metastatic breast cancer, NSCLC, and ovarian cancer; phase III evaluation in recurrent/metastatic HER2-negative breast cancer	
Paclitaxel micellar (Paclical/Apealea)	XR17 surfactant micelles; approximately 20–30 nm	EU authorization issued in 2018; withdrawn on 9 February 2024 at the holder’s request for commercial reasons	Former EU indication with carboplatin: first relapse of platinum-sensitive epithelial ovarian, primary peritoneal, or fallopian tube cancer	
NK105	PEG–poly(aspartate) polymeric micelles; approximately 85 nm	Investigational; multinational phase III and subsequent phase II studies completed; not approved	Metastatic or recurrent breast cancer	
ZSYY001	Methoxy-PEG/lactide polymeric micelles; particle size NR in the phase I article	Investigational; phase I dose-escalation study	Advanced solid tumors	
**Formulation**	**Main Therapeutic or Administration Advantage**	**Key Toxicity or Premedication Issue**	**Current Limitation**	**Reference(s)**
nab-Paclitaxel (Abraxane)	CrEL-free; 30-min infusion for breast cancer and NSCLC; routine corticosteroid/antihistamine premedication is not specified	Severe myelosuppression/neutropenia and sensory neuropathy; consider premedication after a prior hypersensitivity reaction	Benefits and toxicity remain regimen- and indication-dependent; approval does not establish universal active tumor targeting	FDA label [140]; [121,122,123,124,125]
Paclitaxel liposome (Lipusu)	CrEL-free; phase III efficacy was similar to gemcitabine/cisplatin with fewer treatment interruptions and terminations	Taxane-related hematologic and neurologic toxicity persists; corticosteroid/antihistamine premedication has been used in some clinical protocols	Limited global regulatory and head-to-head evidence; no established general tumor-targeting superiority	Zhang et al. [126]; Ye et al. [128]
Genexol-PM	CrEL-free; permits higher PTX doses; phase III objective response rate was higher than with conventional PTX	Grade ≥ 3 neutropenia was more frequent in the phase III comparison; taxane neurotoxicity remains relevant	No significant PFS or OS improvement was demonstrated; approvals and indications differ by jurisdiction	[128,141]
Paclitaxel micellar (Paclical/Apealea)	CrEL-free; no routine premedication; phase III non-inferior PFS at a higher PTX dose	Neutropenia and peripheral neuropathy remained clinically important; hypersensitivity was not eliminated	EU authorization is no longer valid; no clear PFS or OS superiority over solvent-based PTX	EMA EPAR[120]; Vergote et al. [127]
NK105	Lower incidence of severe peripheral sensory neuropathy than conventional PTX in phase III	Overall safety remained broadly similar; conventional taxane toxicities were not abolished	Phase III PFS non-inferiority endpoint was not met (8.4 vs. 8.5 months)	Fujiwara et al. [130]; Kosaka et al. [131]
ZSYY001	Administered over 3 h without premedication; no acute hypersensitivity or dose-limiting toxicity observed up to 390 mg/m^2^	Anemia and alopecia were common; nonlinear pharmacokinetics and the maximum tolerated dose were not resolved	Small early-phase cohort; optimal tumor type and comparative efficacy remain unknown	Gao et al. [132]

Notes: CrEL, Cremophor EL; EMA, European Medicines Agency; EU, European Union; FDA, U.S. Food and Drug Administration; HER2, human epidermal growth factor receptor 2; mPEG–PDLLA, methoxy poly(ethylene glycol)-block-poly(D,L-lactide); NR, not reported; NSCLC, non-small cell lung cancer; OS, overall survival; PFS, progression-free survival; PTX, paclitaxel; XR17, proprietary retinoid-derived surfactant mixture used in paclitaxel micellar. Regulatory status reflects information available on 22 August 2026 and may differ across jurisdictions.

## Data Availability

No new data were created or analyzed in this study. Data sharing is not applicable to this article.

## Abbreviations

The following abbreviations are used in this manuscript:

PTX	Paclitaxel
MDR	Multidrug resistance
TME	Tumor microenvironment
MD	Molecular dynamics
ML	Machine learning
AI	Artificial intelligence
CrEL	Cremophor EL
NDDS	Nanodrug delivery systems
EPR	Enhanced permeability and retention
MPS	Mononuclear phagocyte system
ECM	Extracellular matrix
IFP	Interstitial fluid pressure
GSH	Glutathione
PK	Pharmacokinetic
DEHP	Di (2-ethylhexyl) phthalate
PVC	Polyvinyl chloride
ncRNA	Non-coding RNA
lncRNA	Long non-coding RNA
CIPN	Chemotherapy-induced peripheral neuropathy
PK-PD	Pharmacokinetic-pharmacodynamic
C5aR1	C5a receptor 1
TLR4	Toll-like receptor 4
S1PR1	Sphingosine-1-phosphate receptor 1
DRG	Dorsal root ganglion
BNB	Blood-nerve barrier
TNF-α	Tumor necrosis factor-α
CXCR4	C-X-C motif chemokine receptor 4
DKK1	Dickkopf-1
MMPs	Matrix metalloproteinases
ROS	Reactive oxygen species
HA	Hyaluronic acid
PEG	Polyethylene glycol
Ce6	Chlorin e6
PDT	Photodynamic therapy
DMA	2,3-dimethylmaleic anhydride
siRNA	Small interfering RNA
RBC	Red blood cell
CCM	Cancer cell membrane
EpCAM	Epithelial cell adhesion molecule
MOF	Metal–organic framework
NS	Nanosuspension
TUNEL	Terminal deoxynucleotidyl transferase-mediated dUTP nick-end labeling
H&E	Hematoxylin and eosin
MRI	Magnetic resonance imaging
BBB	Blood–brain barrier
cRGD	Cyclic arginine-glycine-aspartic acid
TNBC	Triple-negative breast cancer
HSP70	Heat shock protein 70
NIR	Near-infrared
VEGF	Vascular endothelial growth factor
HLB	Hydrophilic-lipophilic balance
TPP	Tetraphenylporphyrin
ATGL	Adipose triglyceride lipase
PARP	Poly (ADP-ribose) polymerase
NSCLC	Non-small cell lung cancer
DSPE-PEG	Distearoylphosphatidylethanolamine-polyethylene glycol
CAC	Critical aggregation concentration
PDI	Polydispersity index
TMP	Tetramethylpyrazine
CA4	Combretastatin A-4
DSF	Disulfiram
GCS	Glucosylceramide synthase
MSNs	Mesoporous silica nanoparticles
ATP	Adenosine triphosphate
ABC	ATP-binding cassette
pHe	Extracellular pH
NLS	Nuclear localization signal
DDR	DNA damage response
CAF	Cancer-associated fibroblast
TGF-β	Transforming growth factor-β
shRNA	Short hairpin RNA
Rh123	Rhodamine 123
CI	Combination index
%ID	Percent of the injected dose
PLGA	Poly(lactic-co-glycolic acid)
AMF	Alternating magnetic fields
PTT	Photothermal therapy
SPION	Superparamagnetic iron oxide nanoparticle
HSA	Human serum albumin
FDA	Food and Drug Administration
SPARC	Secreted protein acidic and rich in cysteine
ICIs	Immune checkpoint inhibitors
PFS	Progression-free survival
PD-L1	Programmed death-ligand 1
ITT	Intention-to-treat
TTFields	Tumor-treating fields
OS	Overall survival
PBPK	Physiologically based pharmacokinetic
PDAC	Pancreatic ductal adenocarcinoma
NPMP	Nanoscale polymeric micelle PTX
mPEG-PLGA	Methoxy poly(ethylene glycol)-poly(lactic-co-glycolic acid)
CSF1R	Colony-stimulating factor 1 receptor
PSMA	Prostate-specific membrane antigen
GMP	Good Manufacturing Practice
CQAs	Critical quality attributes
CMC	Chemistry, manufacturing, and controls
EMT	Epithelial–mesenchymal transition
Cav-1	Caveolin-1
pCR	Pathological complete response
ANNs	Artificial neural networks
GCMC	Grand canonical Monte Carlo
SVM	Support vector machine
AUC	Area under the curve
ctDNA	Circulating tumor DNA
CXCL12/SDF-1	C-X-C motif chemokine ligand 12/stromal cell-derived factor-1
Cmax	maximum plasma concentration
PEGPH20	PEGylated recombinant human hyaluronidase

## References

[1] Bray F., Laversanne M., Sung H., Ferlay J., Siegel R.L., Soerjomataram I., Jemal A. (2024). Global cancer statistics 2022: GLOBOCAN estimates of incidence and mortality worldwide for 36 cancers in 185 countries. CA Cancer J. Clin..

[2] Naghavi M., Ong K.L., Aali A., Ababneh H.S., Abate Y.H., Abbafati C., Abbasgholizadeh R., Abbasian M., Abbasi-Kangevari M., Abbastabar H. (2024). Global burden of 288 causes of death and life expectancy decomposition in 204 countries and territories and 811 subnational locations, 1990–2021: A systematic analysis for the Global Burden of Disease Study 2021. Lancet.

[3] DeVita V.T., Chu E. (2008). A history of cancer chemotherapy. Cancer Res..

[4] Wani M.C., Taylor H.L., Wall M.E., Coggon P., McPhail A.T. (1971). Plant antitumor agents. VI. Isolation and structure of taxol, a novel antileukemic and antitumor agent from Taxus brevifolia. J. Am. Chem. Soc..

[5] Bernabeu E., Cagel M., Lagomarsino E., Moretton M., Chiappetta D.A. (2017). Paclitaxel: What has been done and the challenges remain ahead. Int. J. Pharm..

[6] Al-Mahayri Z.N., AlAhmad M.M., Ali B.R. (2021). Current opinion on the pharmacogenomics of paclitaxel-induced toxicity. Expert Opin. Drug Metab. Toxicol..

[7] Seyam S., Alallam B., Harif Fadzilah N., Abd Kadir E. (2025). Advances in paclitaxel nanoformulations: A systematic review of in vivo therapeutic efficacy and safety enhancements. J. Control. Release.

[8] Coley H.M. (2008). Mechanisms and strategies to overcome chemotherapy resistance in metastatic breast cancer. Cancer Treat. Rev..

[9] Pallares R.M., Barmin R.A., Wang A., Kiessling F., Lammers T. (2025). Clinical cancer nanomedicines. J. Control. Release.

[10] Chen Q., Yuan L., Chou W.C., Cheng Y.H., He C., Monteiro-Riviere N.A., Riviere J.E., Lin Z. (2023). Meta-Analysis of Nanoparticle Distribution in Tumors and Major Organs in Tumor-Bearing Mice. ACS Nano.

[11] Sun R., Xiang J., Zhou Q., Piao Y., Tang J., Shao S., Zhou Z., Bae Y.H., Shen Y. (2022). The tumor EPR effect for cancer drug delivery: Current status, limitations, and alternatives. Adv. Drug Deliv. Rev..

[12] Xu X., Liu A., Liu S., Ma Y., Zhang X., Zhang M., Zhao J., Sun S., Sun X. (2023). Application of molecular dynamics simulation in self-assembled cancer nanomedicine. Biomater. Res..

[13] Meng X., Shen Y., Zhao H., Lu X., Wang Z., Zhao Y. (2024). Redox-manipulating nanocarriers for anticancer drug delivery: A systematic review. J. Nanobiotechnol..

[14] Zou L., Liu X., Li J., Li W., Zhang L., Fu C., Zhang J., Gu Z. (2021). Redox-sensitive carrier-free nanoparticles self-assembled by disulfide-linked paclitaxel-tetramethylpyrazine conjugate for combination cancer chemotherapy. Theranostics.

[15] Jang Y., Babu A., Chahal S., Vasukutty A., Moon J.J., Park I.-K., Park H. (2025). AI-guided design of a CXCR4-targeted core-shell nanocarrier for co-delivery of berberine/paclitaxel in cancer therapy. J. Nanobiotechnol..

[16] Liu Y., Zhao F., Wang Q., Zhao Q., Hou G., Meng Q. (2023). Current Perspectives on Paclitaxel: Focus on Its Production, Delivery and Combination Therapy. Mini Rev. Med. Chem..

[17] McClune C.J., Liu J.C.-T., Wick C., De La Peña R., Lange B.M., Fordyce P.M., Sattely E.S. (2025). Discovery of FoTO1 and Taxol genes enables biosynthesis of baccatin III. Nature.

[18] Li C., Qi Y., Sun Z., Jiang M., Li C. (2023). Way to efficient microbial paclitaxel mass production. Synth. Syst. Biotechnol..

[19] Wang L., Zhao X., Yang F., Wu W., Wu M., Li Y., Zhang X. (2019). Loading paclitaxel into porous starch in the form of nanoparticles to improve its dissolution and bioavailability. Int. J. Biol. Macromol..

[20] Zhu S., Sun C., Cai Z., Li Y., Liu W., Luan Y., Wang C. (2024). Effective therapy of advanced breast cancer through synergistic anticancer by paclitaxel and P-glycoprotein inhibitor. Mater. Today Bio.

[21] Bergonzini C., Gregori A., Hagens T.M.S., van der Noord V.E., van de Water B., Zweemer A.J.M., Coban B., Capula M., Mantini G., Botto A. (2024). ABCB1 overexpression through locus amplification represents an actionable target to combat paclitaxel resistance in pancreatic cancer cells. J. Exp. Clin. Cancer Res..

[22] Feng X., Zhang D., Wang G., Lu L., Feng F., Wang X., Yu C., Chai Y., Zhang J., Li W. (2025). Mechanisms and Therapeutic Strategies for Minority Cell-Induced Paclitaxel Resistance and Tumor Progression Mediated by Mechanical Forces. Adv. Sci..

[23] Ghafouri-Fard S., Shoorei H., Abak A., Abbas Raza S.H., Pichler M., Taheri M. (2021). Role of non-coding RNAs in modulating the response of cancer cells to paclitaxel treatment. Biomed. Pharmacother..

[24] Wang W., Wang J., Liu S., Ren Y., Wang J., Liu S., Cui W., Jia L., Tang X., Yang J. (2022). An EHMT2/NFYA-ALDH2 signaling axis modulates the RAF pathway to regulate paclitaxel resistance in lung cancer. Mol. Cancer.

[25] Pao Y.-S., Liao K.-J., Shiau Y.-C., Chao M.-H., Li M.-C., Lin L.-M., Chang H.-H., Yeh H.-W., Chen Y.-J., Chiu Y.-T. (2025). KIF2C promotes paclitaxel resistance by depolymerizing polyglutamylated microtubules. Dev. Cell.

[26] Sun Y., Pai M.P., Henry N.L., Hertz D.L. (2025). Pharmacokinetic-Pharmacodynamic Model of Paclitaxel-Induced Peripheral Neuropathy. Clin. Transl. Sci..

[27] Shin G.J.-E. (2023). Towards a mechanistic understanding of axon transport and endocytic changes underlying paclitaxel-induced peripheral neuropathy. Exp. Neurol..

[28] Brandolini L., d’Angelo M., Novelli R., Castelli V., Giorgio C., Sirico A., Cocchiaro P., D’Egidio F., Benedetti E., Cristiano C. (2022). Paclitaxel binds and activates C5aR1: A new potential therapeutic target for the prevention of chemotherapy-induced peripheral neuropathy and hypersensitivity reactions. Cell Death Dis..

[29] Lötsch J., Gasimli K., Malkusch S., Hahnefeld L., Angioni C., Schreiber Y., Trautmann S., Wedel S., Thomas D., Ferreiros Bouzas N. (2024). Machine learning and biological validation identify sphingolipids as potential mediators of paclitaxel-induced neuropathy in cancer patients. eLife.

[30] Liu X., Tonello R., Ling Y., Gao Y.-J., Berta T. (2019). Paclitaxel-activated astrocytes produce mechanical allodynia in mice by releasing tumor necrosis factor-α and stromal-derived cell factor 1. J. Neuroinflammation.

[31] Xu Y., Jiang Z., Chen X. (2022). Mechanisms underlying paclitaxel-induced neuropathic pain: Channels, inflammation and immune regulations. Eur. J. Pharmacol..

[32] Shi H.-X., Tao H.-T., He J.-J., Zhu F.-Y., Xie C.-Q., Cheng Y.-N., Hou L.-L., Sun H., Qin C.-J., Fang D. (2024). Targeting DKK1 enhances the antitumor activity of paclitaxel and alleviates chemotherapy-induced peripheral neuropathy in breast cancer. Mol. Cancer.

[33] Zhang J., Huang Y., Sun X., Chen X., Zhao X., Ran C., Liu B., Hao Y. (2024). Activating autophagy improves paclitaxel-induced peripheral neuropathy in chemotherapy. Acta Pharm. Sin. B.

[34] Uthaman S., Huh K.M., Park I.K. (2018). Tumor microenvironment-responsive nanoparticles for cancer theragnostic applications. Biomater. Res..

[35] Zhang Y., Zhang Z., Zhang B., Zhang D., Zang W., Zheng S., Lu Y., Sun J., Sun B., He Z. (2025). Branched fatty acids in prodrug nanoassemblies: Molecular gating for governing stability and activation efficiency to amplify therapeutic benefits. Chem. Eng. J..

[36] Du Y., Wang Z., Wang T., He W., Zhou W., Li M., Yao C., Li X. (2020). Improved Antitumor Activity of Novel Redox-Responsive Paclitaxel-Encapsulated Liposomes Based on Disulfide Phosphatidylcholine. Mol. Pharm..

[37] Sun B., Luo C., Yu H., Zhang X., Chen Q., Yang W., Wang M., Kan Q., Zhang H., Wang Y. (2018). Disulfide Bond-Driven Oxidation- and Reduction-Responsive Prodrug Nanoassemblies for Cancer Therapy. Nano Lett..

[38] Cui S., Yu L., Liu H., Liu W., Shi D. (2025). Tumor redox heterogeneity-responsive nanoparticles for enhanced antitumor efficacy through combining chemo/chemodynamic therapy. Int. J. Pharm. X.

[39] Yao Q., Kou L., Tu Y., Zhu L. (2018). MMP-Responsive ‘Smart’ Drug Delivery and Tumor Targeting. Trends Pharmacol. Sci..

[40] Kapalatiya H., Madav Y., Tambe V.S., Wairkar S. (2022). Enzyme-responsive smart nanocarriers for targeted chemotherapy: An overview. Drug Deliv. Transl. Res..

[41] Callmann C.E., Barback C.V., Thompson M.P., Hall D.J., Mattrey R.F., Gianneschi N.C. (2015). Therapeutic Enzyme-Responsive Nanoparticles for Targeted Delivery and Accumulation in Tumors. Adv. Mater..

[42] Guo F., Du Y., Wang Y., Wang M., Wang L., Yu N., Luo S., Wu F., Yang G. (2024). Targeted drug delivery systems for matrix metalloproteinase-responsive anoparticles in tumor cells: A review. Int. J. Biol. Macromol..

[43] Hao D., Meng Q., Jiang B., Lu S., Xiang X., Pei Q., Yu H., Jing X., Xie Z. (2022). Hypoxia-Activated PEGylated Paclitaxel Prodrug Nanoparticles for Potentiated Chemotherapy. ACS Nano.

[44] Lee H., Dey D.K., Kim K., Kim S., Kim E., Kang S.C., Bajpai V.K., Huh Y.S. (2022). Hypoxia-responsive nanomedicine to overcome tumor microenvironment-mediated resistance to chemo-photodynamic therapy. Mater. Today Adv..

[45] Zhang P., Chen D., Li L., Sun K. (2022). Charge reversal nano-systems for tumor therapy. J. Nanobiotechnol..

[46] Wong C., Stylianopoulos T., Cui J., Martin J., Chauhan V.P., Jiang W., Popovic Z., Jain R.K., Bawendi M.G., Fukumura D. (2011). Multistage nanoparticle delivery system for deep penetration into tumor tissue. Proc. Natl. Acad. Sci. USA.

[47] Sheng J., Yuan W., Zhang M., Chen Z., Qi Y., Zhao Y., Zhang S. (2026). Overcoming tumor microenvironment barriers: Transformable and bioinspired nanomedicine strategies for deep tumor penetration. J. Nanobiotechnol..

[48] Lou X., Zhang D., Ling H., He Z., Sun J., Sun M., Liu D. (2021). Pure redox-sensitive paclitaxel-maleimide prodrug nanoparticles: Endogenous albumin-induced size switching and improved antitumor efficiency. Acta Pharm. Sin. B.

[49] Sun X., Zhang J., Yang C., Huang Z., Shi M., Pan S., Hu H., Qiao M., Chen D., Zhao X. (2019). Dual-Responsive Size-Shrinking Nanocluster with Hierarchical Disassembly Capability for Improved Tumor Penetration and Therapeutic Efficacy. ACS Appl. Mater. Interfaces.

[50] Huo Q., Zhu J., Niu Y., Shi H., Gong Y., Li Y., Song H., Liu Y. (2017). PH-triggered surface charge-switchable polymer micelles for the co-delivery of paclitaxel/disulfiram and overcoming multidrug resistance in cancer. Int. J. Nanomed..

[51] Badparvar F., Marjani A.P., Salehi R., Ramezani F. (2024). Dual pH/redox-responsive hyperbranched polymeric nanocarriers with TME-trigger size shrinkage and charge reversible ability for amplified chemotherapy of breast cancer. Sci. Rep..

[52] Jia W., Liu R., Wang Y., Hu C., Yu W., Zhou Y., Wang L., Zhang M., Gao H., Gao X. (2022). Dual-responsive nanoparticles with transformable shape and reversible charge for amplified chemo-photodynamic therapy of breast cancer. Acta Pharm. Sin. B.

[53] Wang H., Li J., Xu J., Hu Y., Zuo Y., Li J. (2023). Enzyme/pH Dual-Responsive Size-Shrinkable Nanocomposites for Tumor Penetration and Enhanced Multimodal Cancer Therapy. ACS Appl. Nano Mater..

[54] Oldenborg P.A., Zheleznyak A., Fang Y.F., Lagenaur C.F., Gresham H.D., Lindberg F.P. (2000). Role of CD47 as a marker of self on red blood cells. Science.

[55] Song M., Dong S., An X., Zhang W., Shen N., Li Y., Guo C., Liu C., Li X., Chen S. (2022). Erythrocyte-biomimetic nanosystems to improve antitumor effects of paclitaxel on epithelial cancers. J. Control. Release.

[56] Guo Q., Wang S., Xu R., Tang Y., Xia X. (2024). Cancer cell membrane-coated nanoparticles: A promising anti-tumor bionic platform. RSC Adv..

[57] Wu Q., Chon J.S.D., Feng Y., Liang K., Li S., Zhang C.Y., Ge J. (2026). Biomimetic Cell Membrane-Coated MOFs System for Targeted Cancer Therapy. Adv. Sci..

[58] Fan Y., Cui Y., Hao W., Chen M., Liu Q., Wang Y., Yang M., Li Z., Gong W., Song S. (2021). Carrier-free highly drug-loaded biomimetic nanosuspensions encapsulated by cancer cell membrane based on homology and active targeting for the treatment of glioma. Bioact. Mater..

[59] Liu X., He J., Ying H., Chen C., Zheng C., Luo P., Zhu W., Wei T., Tang B., Zhang J. (2025). Targeting PFKFB4 Biomimetic Codelivery System Synergistically Enhances Ferroptosis to Suppress Small Cell Lung Cancer and Augments the Efficacy of Anti-PD-L1 Immunotherapy. Adv. Sci..

[60] Yan Y., Li X.Q., Duan J.L., Bao C.J., Cui Y.N., Su Z.B., Xu J.R., Luo Q., Chen M., Xie Y. (2019). Nanosized functional miRNA liposomes and application in the treatment of TNBC by silencing Slug gene. Int. J. Nanomed..

[61] Sun C., Xie F., Zhang H., Feng L., Wang Y., Huang C., Cui Z., Luo C., Zhang L., Wang Q. (2025). Paclitaxel/Luteolin Coloaded Dual-Functional Liposomes for Esophageal Cancer Therapy. Adv. Sci..

[62] Hu Q., Sun W., Qian C., Bomba H.N., Xin H., Gu Z. (2017). Relay Drug Delivery for Amplifying Targeting Signal and Enhancing Anticancer Efficacy. Adv. Mater..

[63] Aili M., Lin F., Chen Y., Alifu N., Ma R., Chu C., Xiong J., Cui Y., Du Z., Ma C. (2025). Overcoming chemoresistance via NO-mediated HSP inhibition using hybrid membrane-coated multifunctional nanoplatforms. Mater. Today Bio.

[64] Zhou M., Wu Y., Sun M., Qin Y., Zhao J., Qiu Z., Li C., Zhang Y., Xiong Y., Shen Y. (2024). Spatiotemporally sequential delivery of biomimetic liposomes potentiates glioma chemotherapy. J. Control. Release.

[65] Li Z., Li M., Lu J. (2026). Biomimetic liposomes in drug delivery: From design mechanisms to applications. Chem. Soc. Rev..

[66] Ding Y., Xu Q., Chai Z., Wu S., Xu W., Wang J., Zhou J., Luo Z., Liu Y., Xie C. (2024). All-stage targeted red blood cell membrane-coated docetaxel nanocrystals for glioma treatment. J. Control. Release.

[67] Wang S., Bager C.L., Karsdal M.A., Chondros D., Taverna D., Willumsen N. (2021). Blood-based extracellular matrix biomarkers as predictors of survival in patients with metastatic pancreatic ductal adenocarcinoma receiving pegvorhyaluronidase alfa. J. Transl. Med..

[68] Sonabend A.M., Gould A., Amidei C., Ward R., Schmidt K.A., Zhang D.Y., Gomez C., Bebawy J.F., Liu B.P., Bouchoux G. (2023). Repeated blood–brain barrier opening with an implantable ultrasound device for delivery of albumin-bound paclitaxel in patients with recurrent glioblastoma: A phase 1 trial. Lancet Oncol..

[69] Zhang D.Y., Dmello C., Chen L., Arrieta V.A., Gonzalez-Buendia E., Kane J.R., Magnusson L.P., Baran A., James C.D., Horbinski C. (2020). Ultrasound-mediated Delivery of Paclitaxel for Glioma: A Comparative Study of Distribution, Toxicity, and Efficacy of Albumin-bound Versus Cremophor Formulations. Clin. Cancer Res..

[70] Kalogera E., Nevala W.K., Finnes H.D., Suman V.J., Schimke J.M., Strand C.A., Kottschade L.A., Kudgus R.A., Buhrow S.A., Becher L.R. (2024). A Phase I Trial of Nab-Paclitaxel/Bevacizumab (AB160) Nano-Immunoconjugate Therapy for Gynecologic Malignancies. Clin. Cancer Res..

[71] Fountzilas C., Javle M., Tan W., Ma Y., Fetterly G., Iyer R., Johnson C. (2018). A phase 1, open-label, dose escalation study of intravenous paricalcitol in combination with gemcitabine in patients with advanced malignancies. Cancer.

[72] Tian B., Xu H., Wang H., Li K., Zheng S., Hu S., Wang Y., Lv Q. (2023). GSH-Responsive Prodrug Nanoassembly as a Carrier-Free Nanoplatform for Tumor-Targeting Delivery and Chemo-Photothermal Therapy. Mol. Pharm..

[73] Yang C., Zhao Y., Jiao Y., Yang L., He B., Dai W., Zhang H., Zhang Q., Wang X. (2024). Organelle-Level Trafficking and Metabolism Kinetics for Redox-Responsive Paclitaxel Prodrug Nanoparticles Characterized by Experimental and Modeling Analysis. ACS Nano.

[74] Zhong W., Xu Y., Wang Z., Wang X., Li Y., Liu J., Zhao C., Shi X., He Z., Sun B. (2025). Dual role of triglyceride structures facilitates anti-tumor drug delivery: Both as a self-assembling module and a responsive module. J. Colloid Interface Sci..

[75] Wang D., Huang Y., Yuan J., Wang S., Sheng J., Zhao Y., Zhang H., Wang X., Yu Y., Shi X. (2024). Exploring the optimal chain length of modification module in disulfide bond bridged paclitaxel prodrug nanoassemblies for breast tumor treatment. J. Control. Release.

[76] Zhang C., Chen Y., Shi X., Liu Y., Zhou J., Leng L., Zhang T., Liu S., Liu J., Wang T. (2026). Self-assembled multicomponent prodrugs with GSH/ROS site-responsiveness enable spatiotemporally controlled release for treating resistant NSCLC. J. Control. Release.

[77] Zhang B., Zhou S., Lu S., Xiang X., Yao X., Lei W., Pei Q., Xie Z., Chen X. (2024). Paclitaxel Prodrug Enables Glutathione Depletion to Boost Cancer Treatment. ACS Nano.

[78] Chen H., Xing C., Lei H., Yan B., Zhang H., Tong T., Guan Y., Kang Y., Pang J. (2024). ROS-driven supramolecular nanoparticles exhibiting efficient drug delivery for chemo/Chemodynamic combination therapy for Cancer treatment. J. Control. Release.

[79] Zhou H., Yang Z., Jin G., Wang L., Su Y., Liu H., Sun H., Xue L., Mi L., Veselova I.A. (2025). Prodrug-designed nanocarrier co-delivering chemotherapeutic and vascular disrupting agents with exceptionally high drug loading capacity. J. Control. Release.

[80] Zou J., Xing X., Teng C., Zhao Q., He W., Wu X., Xia Y. (2024). Cocrystal@protein-anchoring nanococktail for combinatorially treating multidrug-resistant cancer. Acta Pharm. Sin. B.

[81] Saklani R., Yadav P.K., Tiwari A.K., Gawali S.L., Hassan P.A., Yadav K., Mugale M.N., Kalleti N., Rath S.K., Mishra D.P. (2024). Synchronized Codelivery of Combination Chemotherapies Intratumorally Using a Lipidic Lyotropic Liquid Crystal System. ACS Appl. Mater. Interfaces.

[82] Wang Y., Chen L., Zhang Z., Liu W., Li L. (2022). Ratiometric co-delivery of doxorubicin and paclitaxel prodrug by remote-loading liposomes for the treatment of triple-negative breast cancer. Drug Deliv. Transl. Res..

[83] Miao L., Guo S., Lin C.M., Liu Q., Huang L. (2017). Nanoformulations for combination or cascade anticancer therapy. Adv. Drug Deliv. Rev..

[84] Xu J., Zhao W., Sun J., Huang Y., Wang P., Venkataramanan R., Yang D., Ma X., Rana A., Li S. (2018). Novel glucosylceramide synthase inhibitor based prodrug copolymer micelles for delivery of anticancer agents. J. Control. Release.

[85] Guo Y., Ding L., Lang X., You J., Wu D., Wang X., Zhang Y., Liu W., Dong Z. (2025). Dual-targeting chemoimmunotherapy co-delivery system based on self-assembly and core-shell structure. Biomed. Pharmacother..

[86] Tarannum M., Hossain M.A., Holmes B., Yan S., Mukherjee P., Vivero-Escoto J.L. (2022). Advanced Nanoengineering Approach for Target-Specific, Spatiotemporal, and Ratiometric Delivery of Gemcitabine-Cisplatin Combination for Improved Therapeutic Outcome in Pancreatic Cancer. Small.

[87] Wang H., Ellipilli S., Lee W.J., Li X., Vieweger M., Ho Y.S., Guo P. (2021). Multivalent rubber-like RNA nanoparticles for targeted co-delivery of paclitaxel and MiRNA to silence the drug efflux transporter and liver cancer drug resistance. J. Control. Release.

[88] Wang C., Guan W., Peng J., Chen Y., Xu G., Dou H. (2020). Gene/paclitaxel co-delivering nanocarriers prepared by framework-induced self-assembly for the inhibition of highly drug-resistant tumors. Acta Biomater..

[89] Wang Z., Li X., Wang D., Zou Y., Qu X., He C., Deng Y., Jin Y., Zhou Y., Zhou Y. (2017). Concurrently suppressing multidrug resistance and metastasis of breast cancer by co-delivery of paclitaxel and honokiol with pH-sensitive polymeric micelles. Acta Biomater..

[90] Xia H., Zhou W., Li D., Peng F., Yu L., Sang Y., Liu H., Hao A., Qiu J. (2024). Generation of a Hydrophobic Protrusion on Nanoparticles to Improve the Membrane-Anchoring Ability and Cellular Internalization. Angew. Chem. Int. Ed. Engl..

[91] Fan G.L., Deng F.A., Zhou X., Liu L.S., Huang J.Q., Zhang D.W., Sun Y.X., Chen A.L., Cheng H., Li S.Y. (2021). Plasma membrane targeted photodynamic O_2_ economizer for hypoxic tumor therapy. Biomaterials.

[92] Chang E., Bu J., Ding L., Lou J.W.H., Valic M.S., Cheng M.H.Y., Rosilio V., Chen J., Zheng G. (2021). Porphyrin-lipid stabilized paclitaxel nanoemulsion for combined photodynamic therapy and chemotherapy. J. Nanobiotechnol..

[93] Wang X., Tong J., He Z., Yang X., Meng F., Liang H., Zhang X., Luo L. (2020). Paclitaxel-Potentiated Photodynamic Theranostics for Synergistic Tumor Ablation and Precise Anticancer Efficacy Monitoring. ACS Appl. Mater. Interfaces.

[94] Lin J., Zhao C., Liu C., Fu S., Han L., Lu X., Yang C. (2018). Redox-responsive F127-folate/F127-disulfide bond-d-α-tocopheryl polyethylene glycol 1000 succinate/P123 mixed micelles loaded with paclitaxel for the reversal of multidrug resistance in tumors. Int. J. Nanomed..

[95] Gong Y., Deng Z., Wu J., Hu Y. (2026). Ferrodoxin 1 (FDX1) drives paclitaxel resistance in ovarian cancer via copper metabolism and ULK1/ATG13-mediated autophagy: Overcome by pH/ROS-responsive PPD/PDP@si-FDX1 nanomicelles. J. Exp. Clin. Cancer Res..

[96] Zhu L., Guo Y., Qian Q., Yan D., Li Y., Zhu X., Zhang C. (2020). Carrier-Free Delivery of Precise Drug-Chemogene Conjugates for Synergistic Treatment of Drug-Resistant Cancer. Angew. Chem. Int. Ed. Engl..

[97] Cao Z., Li D., Wang J., Xiong M., Yang X. (2019). Direct Nucleus-Targeted Drug Delivery Using Cascade pH_e_/Photo Dual-Sensitive Polymeric Nanocarrier for Cancer Therapy. Small.

[98] Singla P., Broughton T., Sullivan M.V., Garg S., Berlinguer-Palmini R., Gupta P., Smith K.J., Gardner B., Canfarotta F., Turner N.W. (2024). Double Imprinted Nanoparticles for Sequential Membrane-to-Nuclear Drug Delivery. Adv. Sci..

[99] Li N., Yang H., Yu Z., Li Y., Pan W., Wang H., Tang B. (2017). Nuclear-targeted siRNA delivery for long-term gene silencing. Chem. Sci..

[100] Deng R., Shen N., Yang Y., Yu H., Xu S., Yang Y.W., Liu S., Meguellati K., Yan F. (2018). Targeting epigenetic pathway with gold nanoparticles for acute myeloid leukemia therapy. Biomaterials.

[101] Cai Y., Shen H., Zhan J., Lin M., Dai L., Ren C., Shi Y., Liu J., Gao J., Yang Z. (2017). Supramolecular “Trojan Horse” for Nuclear Delivery of Dual Anticancer Drugs. J. Am. Chem. Soc..

[102] Chen Q., Jia C., Xu Y., Jiang Z., Hu T., Li C., Cheng X. (2022). Dual-pH responsive chitosan nanoparticles for improving in vivo drugs delivery and chemoresistance in breast cancer. Carbohydr. Polym..

[103] Zheng Z., Lang T., Huang X., Wang G., Lee R.J., Teng L., Yin Q., Li Y. (2020). Calcitriol-Loaded Dual-pH-Sensitive Micelle Counteracts Pro-Metastasis Effect of Paclitaxel in Triple-Negative Breast Cancer Therapy. Adv. Healthc. Mater..

[104] Zhang M., Zhang B. (2025). Extracellular matrix stiffness: Mechanisms in tumor progression and therapeutic potential in cancer. Exp. Hematol. Oncol..

[105] Yin S., Gao Y., Zhang Y., Xu J., Zhu J., Zhou F., Gu X., Wang G., Li J. (2020). Reduction/Oxidation-Responsive Hierarchical Nanoparticles with Self-Driven Degradability for Enhanced Tumor Penetration and Precise Chemotherapy. ACS Appl. Mater. Interfaces.

[106] Fang T., Zhang J., Zuo T., Wu G., Xu Y., Yang Y., Yang J., Shen Q. (2020). Chemo-Photothermal Combination Cancer Therapy with ROS Scavenging, Extracellular Matrix Depletion, and Tumor Immune Activation by Telmisartan and Diselenide-Paclitaxel Prodrug Loaded Nanoparticles. ACS Appl. Mater. Interfaces.

[107] Zhang X., Ren X., Tang J., Wang J., Zhang X., He P., Yao C., Bian W., Sun L. (2020). Hyaluronic acid reduction-sensitive polymeric micelles achieving co-delivery of tumor-targeting paclitaxel/apatinib effectively reverse cancer multidrug resistance. Drug Deliv..

[108] Jia L., Li Z., Zheng D., Li Z., Zhao Z. (2021). A targeted and redox/pH-responsive chitosan oligosaccharide derivatives based nanohybrids for overcoming multidrug resistance of breast cancer cells. Carbohydr. Polym..

[109] Gregory J.V., Vogus D.R., Barajas A., Cadena M.A., Mitragotri S., Lahann J. (2020). Programmable Delivery of Synergistic Cancer Drug Combinations Using Bicompartmental Nanoparticles. Adv. Healthc. Mater..

[110] Yu L., Zhou X., Liu Z., Liu H., Zhang X.Z., Luo G.F., Shang Z. (2024). Carrier-Free Nanoagent Interfering with Cancer-Associated Fibroblasts’ Metabolism to Promote Tumor Penetration for Boosted Chemotherapy. Nano Lett..

[111] Guo Y., Liu S., Luo F., Tang D., Yang T., Yang X., Xie Y. (2022). A Nanosized Codelivery System Based on Intracellular Stimuli-Triggered Dual-Drug Release for Multilevel Chemotherapy Amplification in Drug-Resistant Breast Cancer. Pharmaceutics.

[112] Lee S.B., Park J.M., Park R., Choi H.E., Hong S.W., Kim K.S. (2025). Synergistic chemo-photothermal treatment via MXene-encapsulated nanoparticles for targeted melanoma therapy. J. Control. Release.

[113] Liu R., Sang L., Wang T., Liu Y., Wang Z., Li J., Wang D. (2021). Phase-change mesoporous Prussian blue nanoparticles for loading paclitaxel and chemo-photothermal therapy of cancer. Colloids Surf. B Biointerfaces.

[114] Hussain B., Ullah A., Ali I., Haider S., Han H.J., Jin G. (2026). Advances in semiconductor materials and device architectures for biomedical systems: A mini review. Biomed. Eng. Lett..

[115] Zhang Y., Pei Q., Yue Y., Xie Z. (2022). Binary dimeric prodrug nanoparticles for self-boosted drug release and synergistic chemo-photodynamic therapy. J. Mater. Chem. B.

[116] Zhou S., Hu X., Xia R., Liu S., Pei Q., Chen G., Xie Z., Jing X. (2020). A Paclitaxel Prodrug Activatable by Irradiation in a Hypoxic Microenvironment. Angew. Chem. Int. Ed. Engl..

[117] He L., Qing F., Li M., Lan D. (2020). Paclitaxel/IR1061-Co-Loaded Protein Nanoparticle for Tumor-Targeted and pH/NIR-II-Triggered Synergistic Photothermal-Chemotherapy. Int. J. Nanomed..

[118] Rong J., Wu X., Tang D., Lv F., Zhang Q., Xiao H., Hu X. (2026). Ultrasound-Triggered Dinuclear Iridium Sonosensitizer Activates Paclitaxel Prodrug for Synergistic Sonodynamic-Chemotherapy. Adv. Healthc. Mater..

[119] Tavakoli M., Maghsoudian S., Rezaei-Aderiani A., Hajiramezanali M., Fatahi Y., Amani M., Sharifikolouei E., Ghahremani M.H., Raoufi M., Motasadizadeh H. (2025). Synergistic effects of paclitaxel and platelet-superparamagnetic iron oxide nanoparticles for targeted chemo-hyperthermia therapy against breast cancer. Colloids Surf. B Biointerfaces.

[120] European Medicines Agency (2024). Apealea: European Public Assessment Report (EPAR).

[121] Tan H., Hu J., Liu S. (2019). Efficacy and safety of nanoparticle albumin-bound paclitaxel in non-small cell lung cancer: A systematic review and meta-analysis. Artif. Cells Nanomed. Biotechnol..

[122] Sharifi-Rad J., Quispe C., Patra J.K., Singh Y.D., Panda M.K., Das G., Adetunji C.O., Michael O.S., Sytar O., Polito L. (2021). Paclitaxel: Application in Modern Oncology and Nanomedicine-Based Cancer Therapy. Oxidative Med. Cell. Longev..

[123] Cho H., Jeon S.I., Ahn C.H., Shim M.K., Kim K. (2022). Emerging Albumin-Binding Anticancer Drugs for Tumor-Targeted Drug Delivery: Current Understandings and Clinical Translation. Pharmaceutics.

[124] Schmid P., Adams S., Rugo H.S., Schneeweiss A., Barrios C.H., Iwata H., Diéras V., Hegg R., Im S.A., Shaw Wright G. (2018). Atezolizumab and Nab-Paclitaxel in Advanced Triple-Negative Breast Cancer. N. Engl. J. Med..

[125] Olawaiye A.B., Gladieff L., O’Malley D.M., Kim J.W., Garbaos G., Salutari V., Gilbert L., Mileshkin L., Devaux A., Hopp E. (2025). Relacorilant and nab-paclitaxel in patients with platinum-resistant ovarian cancer (ROSELLA): An open-label, randomised, controlled, phase 3 trial. Lancet.

[126] Zhang J., Pan Y., Shi Q., Zhang G., Jiang L., Dong X., Gu K., Wang H., Zhang X., Yang N. (2022). Paclitaxel liposome for injection (Lipusu) plus cisplatin versus gemcitabine plus cisplatin in the first-line treatment of locally advanced or metastatic lung squamous cell carcinoma: A multicenter, randomized, open-label, parallel controlled clinical study. Cancer Commun..

[127] Vergote I., Bergfeldt K., Franquet A., Lisyanskaya A.S., Bjermo H., Heldring N., Buyse M., Brize A. (2020). A randomized phase III trial in patients with recurrent platinum sensitive ovarian cancer comparing efficacy and safety of paclitaxel micellar and Cremophor EL-paclitaxel. Gynecol. Oncol..

[128] Ye J., Li R., Yang Y., Dong W., Wang Y., Wang H., Sun T., Li L., Shen Q., Qin C. (2021). Comparative colloidal stability, antitumor efficacy, and immunosuppressive effect of commercial paclitaxel nanoformulations. J. Nanobiotechnol..

[129] Zhang L., Liu Z., Kong C., Liu C., Yang K., Chen H., Huang J., Qian F. (2018). Improving Drug Delivery of Micellar Paclitaxel against Non-Small Cell Lung Cancer by Coloading Itraconazole as a Micelle Stabilizer and a Tumor Vascular Manipulator. Small.

[130] Fujiwara Y., Mukai H., Saeki T., Ro J., Lin Y.-C., Nagai S.E., Lee K.S., Watanabe J., Ohtani S., Kim S.B. (2019). A multi-national, randomised, open-label, parallel, phase III non-inferiority study comparing NK105 and paclitaxel in metastatic or recurrent breast cancer patients. Br. J. Cancer.

[131] Kosaka Y., Saeki T., Takano T., Aruga T., Yamashita T., Masuda N., Koibuchi Y., Osaki A., Watanabe J., Suzuki R. (2022). Multicenter Randomized Open-Label Phase II Clinical Study Comparing Outcomes of NK105 and Paclitaxel in Advanced or Recurrent Breast Cancer. Int. J. Nanomed..

[132] Gao G., Shu P., Tan Y., Zheng T., Fan W., Lu L., Zhou H., Wang Z., Liu L., Liu Z. (2025). Preclinical Development and Phase I Study of ZSYY001, a Polymeric Micellar Paclitaxel for Advanced Solid Tumor. Cancer Med..

[133] Wang F.Y., Huang X.M., Cao Y.Q., Cao J., Ni J., Li K., Lu M., Huang X.E. (2024). Nanoparticle Polymeric Micellar Paclitaxel Versus Paclitaxel for Patients with Advanced Gastric Cancer. J. Gastrointest. Cancer.

[134] Zhou Y., Shi M., Shen B., Wang L., Zhu X., Li X., Peng W., Yan L., Tang Y. (2025). 34P: Polymeric micelles paclitaxel (pm-Pac), carboplatin combined with sintilimab in the first-line treatment of advanced non-squamous non-small cell lung cancer(nsq-NSCLC): Phase II study. J. Thorac. Oncol..

[135] Deng X., Huang X., Dong X., Mao G., Xing W. (2023). Efficacy and safety of nanopaclitaxel formulation for cancer treatment: Evidence from randomized clinical trials. Nanomedicine.

[136] Ma P., Wang G., Men K., Li C., Gao N., Li L. (2024). Advances in clinical application of nanoparticle-based therapy for cancer treatment: A systematic review. Nano TransMed.

[137] Li X., Qin Z., Yuan Q., Song Y., Xu Q., Yang J., Deng X. (2023). Controllable release of self-assembled reduction-sensitive paclitaxel dimer prodrug and tetrandrine nanoparticles promotes synergistic therapy against multidrug-resistant cancer. Biochim. Biophys. Acta Gen. Subj..

[138] Lim C., Hwang D., Yazdimamaghani M., Atkins H.M., Hyun H., Shin Y., Ramsey J.D., Rädler P.D., Mott K.R., Perou C.M. (2023). High-Dose Paclitaxel and its Combination with CSF1R Inhibitor in Polymeric Micelles for Chemoimmunotherapy of Triple Negative Breast Cancer. Nano Today.

[139] Autio K.A., Dreicer R., Anderson J., Garcia J.A., Alva A., Hart L.L., Milowsky M.I., Posadas E.M., Ryan C.J., Graf R.P. (2018). Safety and Efficacy of BIND-014, a Docetaxel Nanoparticle Targeting Prostate-Specific Membrane Antigen for Patients with Metastatic Castration-Resistant Prostate Cancer: A Phase 2 Clinical Trial. JAMA Oncol..

[140] U.S. Food and Drug Administration (2022). ABRAXANE for Injectable Suspension (Paclitaxel Protein-Bound Particles for Injectable Suspension) (Albumin-Bound): Full Prescribing Information.

[141] Park I.H., Sohn J.H., Kim S.B., Lee K.S., Chung J.S., Lee S.H., Kim T.Y., Jung K.H., Cho E.K., Kim Y.S. (2017). An Open-Label, Randomized, Parallel, Phase III Trial Evaluating the Efficacy and Safety of Polymeric Micelle-Formulated Paclitaxel Compared to Conventional Cremophor EL-Based Paclitaxel for Recurrent or Metastatic HER2-Negative Breast Cancer. Cancer Res. Treat..

[142] Youngblood M.W., Kumari A., Kang Y.-T., Gould A., Habashy K., Gomez M., Lingamarla H., Morey T., Chen L., Congivaram H. (2025). Dynamic release of extracellular particles after opening of the blood-brain barrier predicts glioblastoma susceptibility to paclitaxel. Nat. Commun..

[143] Miles D., Gligorov J., André F., Cameron D., Schneeweiss A., Barrios C., Xu B., Wardley A., Kaen D., Andrade L. (2021). Primary results from IMpassion131, a double-blind, placebo-controlled, randomised phase III trial of first-line paclitaxel with or without atezolizumab for unresectable locally advanced/metastatic triple-negative breast cancer. Ann. Oncol..

[144] Wang Y., Fens M.H., van Kronenburg N.C.H., Shi Y., Lammers T., Heger M., van Nostrum C.F., Hennink W.E. (2022). Magnetic beads for the evaluation of drug release from biotinylated polymeric micelles in biological media. J. Control. Release.

[145] Bauer T.A., Schramm J., Fenaroli F., Siemer S., Seidl C.I., Rosenauer C., Bleul R., Stauber R.H., Koynov K., Maskos M. (2023). Complex Structures Made Simple—Continuous Flow Production of Core Cross-Linked Polymeric Micelles for Paclitaxel Pro-Drug-Delivery. Adv. Mater..

[146] Yi Z., Ma X., Tong Q., Ma L., Tan Y., Liu D., Tan C., Chen J., Li X. (2025). A Library of Polyphenol-Amino Acid Condensates for High-Throughput Continuous Flow Production of Nanomedicines with Ultra-High Drug Loading. Adv. Mater..

[147] Siemer S., Bauer T.A., Scholz P., Breder C., Fenaroli F., Harms G., Dietrich D., Dietrich J., Rosenauer C., Barz M. (2021). Targeting Cancer Chemotherapy Resistance by Precision Medicine-Driven Nanoparticle-Formulated Cisplatin. ACS Nano.

[148] Hirata K., Hamamoto Y., Shoji H., Hara H., Kondoh C., Yasui H., Kajiwara T., Baba E., Ando T., Sugimoto N. (2026). Solvent-based or nab-paclitaxel plus ramucirumab for pretreated gastric cancer with peritoneal dissemination and prespecified biomarker analysis (P-SELECT/WJOG10617G): A randomised phase 2 trial in Japan. eClinicalMedicine.

[149] Korbie D., Stirzaker C., Gluz O., Zu Eulenburg C., Nitz U., Christgen M., Kuemmel S., Grischke E.M., Forstbauer H., Braun M. (2025). Immunomodulatory gene networks predict treatment response and survival to de-escalated, anthracycline-free neoadjuvant chemotherapy in triple-negative breast cancer in the WSG-ADAPT-TN trial. Mol. Cancer.

[150] Bozdaganyan M., Fedorov V., Kholina E., Kovalenko I., Gudimchuk N., Orekhov P. (2025). Exploring tubulin-paclitaxel binding modes through extensive molecular dynamics simulations. Sci. Rep..

[151] Natarajan K., Senapati S. (2012). Understanding the basis of drug resistance of the mutants of αβ-tubulin dimer via molecular dynamics simulations. PLoS ONE.

[152] Wang Z., Li W., Jiang Y., Park J., Gonzalez K.M., Wu X., Zhang Q.-Y., Lu J. (2024). Cholesterol-modified sphingomyelin chimeric lipid bilayer for improved therapeutic delivery. Nat. Commun..

[153] Lin C., Sun J., Yang Y., Pan X., Wang S., Li X., Zhang Y., Gao H., Gan C. (2025). Peptide-based nanoassembly enhances ferroptosis in cancer to overcome paclitaxel resistance. J. Control. Release.

[154] Kehrein J., Bunker A., Luxenhofer R. (2024). POxload: Machine Learning Estimates Drug Loadings of Polymeric Micelles. Mol. Pharm..

[155] Moore T.L., Pesce C., Greco A., Pisante C., Avancini G., Di Francesco V., Shamay Y., Decuzzi P. (2025). Unleashing the Power of Machine Learning in Nanomedicine Formulation Development. Adv. Funct. Mater..

[156] Melle F., Menon D., Conniot J., Ostolaza-Paraiso J., Mercado S., Oliveira J., Chen X., Mendes B.B., Conde J., Fairen-Jimenez D. (2025). Rational Design of Metal–Organic Frameworks for Pancreatic Cancer Therapy: From Machine Learning Screening to In Vivo Efficacy. Adv. Mater..

[157] Zhang C., Yuan Y., Xia Q., Wang J., Xu K., Gong Z., Lou J., Li G., Wang L., Zhou L. (2025). Machine Learning-Driven Prediction, Preparation, and Evaluation of Functional Nanomedicines Via Drug–Drug Self-Assembly. Adv. Sci..

[158] Chen W., Zhou H., Zhang M., Shi Y., Li T., Qian D., Yang J., Yu F., Li G. (2024). Novel progressive deep learning algorithm for uncovering multiple single nucleotide polymorphism interactions to predict paclitaxel clearance in patients with nonsmall cell lung cancer. Cancer Innov..

[159] Sundar R., Barr Kumarakulasinghe N., Huak Chan Y., Yoshida K., Yoshikawa T., Miyagi Y., Rino Y., Masuda M., Guan J., Sakamoto J. (2022). Machine-learning model derived gene signature predictive of paclitaxel survival benefit in gastric cancer: Results from the randomised phase III SAMIT trial. Gut.

[160] Shoda K., Xu C., Nagasaka T., Ichikawa D., Goel A. (2025). A machine-learning powered liquid biopsy predicts response to paclitaxel plus ramucirumab in advanced gastric cancer: Results from the prospective IVY trial. Mol. Cancer.

[161] Zhang C., Yang J., Chen S., Sun L., Li K., Lai G., Peng B., Zhong X., Xie B. (2024). Artificial intelligence in ovarian cancer drug resistance advanced 3PM approach: Subtype classification and prognostic modeling. EPMA J..

